# Multi-omic tumor data reveal diversity of molecular mechanisms that correlate with survival

**DOI:** 10.1038/s41467-018-06921-8

**Published:** 2018-10-26

**Authors:** Daniele Ramazzotti, Avantika Lal, Bo Wang, Serafim Batzoglou, Arend Sidow

**Affiliations:** 1 Department of Pathology, Stanford University, Stanford, CA 94305 USA; 2 Department of Computer Science, Stanford University, Stanford, CA 94305 USA; 3 Department of Genetics, Stanford University, Stanford, CA 94305 USA; 4 Present Address: Illumina Mission Bay, 499 Illinois Street, Suite 210, San Francisco, CA 94158-250 USA

## Abstract

Outcomes for cancer patients vary greatly even within the same tumor type, and characterization of molecular subtypes of cancer holds important promise for improving prognosis and personalized treatment. This promise has motivated recent efforts to produce large amounts of multidimensional genomic (multi-omic) data, but current algorithms still face challenges in the integrated analysis of such data. Here we present Cancer Integration via Multikernel Learning (CIMLR), a new cancer subtyping method that integrates multi-omic data to reveal molecular subtypes of cancer. We apply CIMLR to multi-omic data from 36 cancer types and show significant improvements in both computational efficiency and ability to extract biologically meaningful cancer subtypes. The discovered subtypes exhibit significant differences in patient survival for 27 of 36 cancer types. Our analysis reveals integrated patterns of gene expression, methylation, point mutations, and copy number changes in multiple cancers and highlights patterns specifically associated with poor patient outcomes.

## Abstract (web summary)

Identifying molecular subtypes of cancer can improve personalized treatment. Here the authors present CIMLR, an algorithm that integrates multi-omic data to reveal cancer subtypes; subtypes discovered by CIMLR differ in activity of cancer-associated pathways and are significantly predictive of patient outcomes.

## Introduction

Cancer is a heterogeneous disease that evolves through many pathways, involving changes in the activity of multiple oncogenes and tumor suppressor genes. The basis for such changes is the vast number and diversity of somatic alterations that produce complex molecular and cellular phenotypes, influencing each individual tumor’s behavior and response to treatment. Due to the diversity of mutations and molecular mechanisms, outcomes vary greatly. It is therefore important to identify cancer subtypes based on common molecular features, and correlate those with outcomes. This will lead to an improved understanding of the pathways by which cancer commonly evolves, as well as better prognosis and personalized treatment.

Efforts to distinguish subtypes are complicated by the many kinds of genomic changes that contribute to cancer. While gene expression clustering is often used to discover subtypes (e.g., the *PAM50* subtypes^1^ of breast cancer), analysis of a single data type does not typically capture the full complexity of a tumor genome and its molecular phenotypes. For example, a copy number change may be relevant only if it causes a gene expression change; gene expression data ignores point mutations that alter the function of the gene product; and point mutations in two different genes may have the same downstream effect, which may become apparent only when also considering methylation or gene expression. Therefore, comprehensive molecular subtyping requires integration of multiple data types.

In order to use multiple data types for subtyping, some approaches carry out separate clustering of each data type followed by manual integration of the clusters^2^. However, clusters based on different data may not be clearly correlated. More rigorous methods for integration include pathway analysis on multi-omic data, followed by clustering on the inferred pathway activities^3^, similarity network fusion (SNF)^4^, rank matrix factorization^5^, and Bayesian consensus clustering^6^. There are also several sparse clustering methods, such as iCluster+^7^, which assume that only a small fraction of features are relevant. These methods are either highly dependent on feature selection, or enforce sparsity, thus neglecting potentially useful information. A recent method, Perturbation clustering for data INtegration and disease Subtyping (PINS)^8^, introduces a novel strategy of identifying clusters that are stable in response to repeated perturbation of the data.

One drawback common to many of the more principled methods is that they are computationally too intensive to be routinely applied to large data sets, due to the need for parameter selection or repeated perturbations. Moreover, they treat all data types equally, which may not be biologically appropriate. As a result, the discovered clusters often show poor association with patient outcomes^9,10^. We therefore set out to develop a novel method that does not have these drawbacks.

Cancer Integration via Multikernel LeaRning (CIMLR) is based on Single-cell Interpretation via Multi-kernel LeaRning (SIMLR), an algorithm for analysis of single-cell RNA-Seq data^11^. CIMLR learns a measure of similarity between each pair of samples in a multi-omic dataset by combining multiple gaussian kernels per data type, corresponding to different, complementary representations of the data. It enforces a block structure in the resulting similarity matrix, which is then used for dimension reduction and *k*-means clustering. CIMLR is capable of incorporating complete genomes and scaling to many data types, and does not assume equal importance for each data type. As such, it is well suited to modeling the heterogeneity of cancer data.

Here, we apply CIMLR to discover integrative subtypes within 36 types of cancer. We recover known as well as novel subtypes, and show that our method outperforms current state-of-the-art tools in speed, accuracy, and prediction of patient survival. This systematic subtype analysis, the most comprehensive to date, provides valuable insights into the biology underlying tumor variability.

## Results

### Subtyping of 36 cancer types using CIMLR

**Fig. 1:**
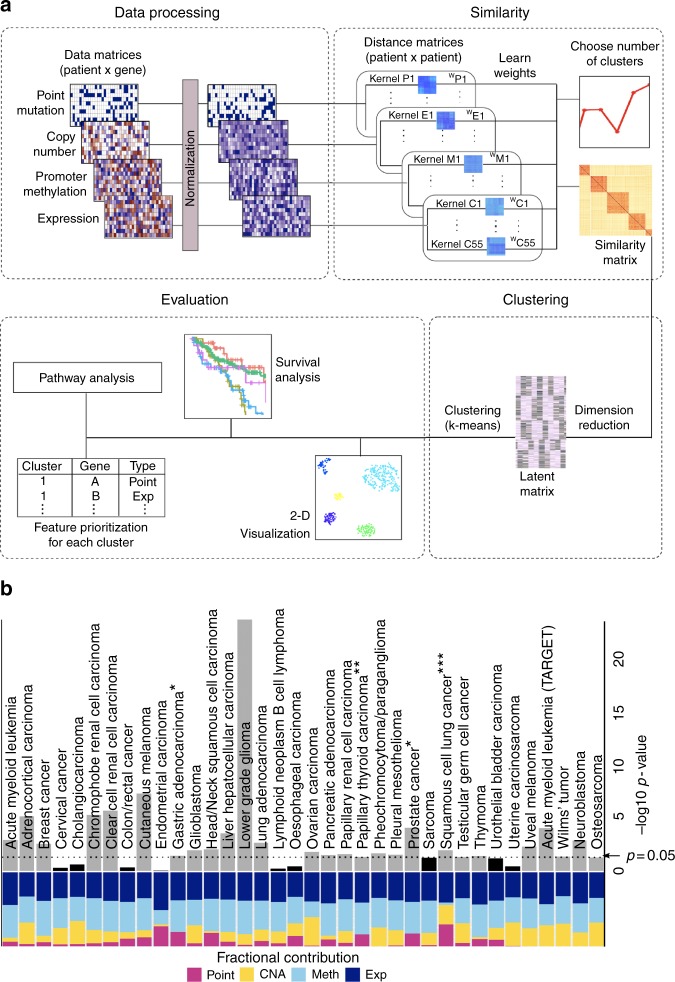
CIMLR overview and survival analysis. **a** CIMLR workflow. Each data type is arranged as a matrix where rows are patients and columns are genes. All matrices are then normalized so that values range from 0 to 1, so that all data types have the same range. For each data type, CIMLR learns weights for multiple kernels (each kernel is a measure of patient-to-patient distance). The number of clusters *C* is determined by a heuristic based on the gap statistic. The method then combines the multiple kernels into a symmetric similarity matrix with *C* blocks, where each block is a set of patients highly similar to each other. The learned similarity matrix is then used for dimension reduction and clustering into subtypes. The clusters are evaluated by visualization as a 2-D scatter plot and survival analysis. The molecular features significantly enriched in each cluster are listed, and finally, pathway activity is compared. **b** Left: Contributions (measured as fraction of total kernel weight) by each data type. Right: Results of survival analysis on the best clusters for 36 cancer types. Gray bars represent the 27 cancer types for which significant differences in patient survival were obtained between clusters; black bars represent the remaining cancers. *PFI; **DSS; ***DFI. Otherwise: overall survival

We carried out a systematic subtype analysis using CIMLR (Fig. 1a) across all 32 cancer types available from TCGA, on a total of 6645 patients. Four data types were considered: point mutations, copy number alterations, promoter CpG methylation, and gene expression.

We evaluated the clusters produced by CIMLR based on (1) survival analysis, (2) silhouette (a measure of cohesion and separation of clusters^12^), (3) stability of the clusters, and (4) significant differences in pathway activity between clusters (Table 1, Supplementary Table 1, Supplementary Table 2). To demonstrate the value of multi-omic subtyping, we compared the performance of CIMLR using all four data types against analysis using only methylation or expression (Table 1, Supplementary Data 1). We also compared CIMLR to four existing methods for integrative subtyping: iCluster+^7^, Bayesian consensus clustering^6^, PINS^8^, and SNF^4^. CIMLR outperformed all other methods on all tested metrics (Table 1, Supplementary Data 2). In particular, the clusters obtained using CIMLR show significant differences in patient survival in 23 of 32 cancer types from TCGA (Fig. 1b), exceeding the performance of all other approaches.

**Table 1:** Comparison of CIMLR with other integrative subtyping methods across 36 cancer types

	CIMLR (multi-omic)	CIMLR (methylation only)	CIMLR (expression only)	iCluster+^7^	BayesCC^6^	PINS^8^	SNF^4^
*TCGA (32 cancers)*							
Overall survival	19	12	12	14	10	12	12
All survival metrics	23	14	20	18	11	17	16
Silhouette	0.81 ± 0.22	0.81 ± 0.09	0.84 ± 0.09	−0.07 ± 0.54	0.09 ± 0.08	0.00 ± 0.07	0.00 ± 0.00
Stability	0.89 ± 0.08	0.91 ± 0.05	0.91 ± 0.04	0.25 ± 0.32	NA	NA	NA
Pathway activity	26	24	26	17	21	26	25
*TARGET* ^13^ *(4 cancers)*							
Overall survival	4	2	3	1	0	1	3
Silhouette	0.86 ± 0.05	0.83 ± 0.08	0.84 ± 0.09	0.26 +/- 0.16	0.09 ± 0.11	0.06 ± 0.10	−0.20 ± 0.03
Stability	0.94 ± 0.04	0.95 ± 0.06	0.93 ± 0.05	0.68 +/- 0.14	NA	NA	NA
*All (36 cancers)*							
All survival metrics	27	16	23	19	11	18	19

Overall survival: Number of cancer types for which a significant (log-rank *p* < 0.05) difference in overall survival was observed between clusters. All survival metrics: number of cancer types for which a significant (log-rank *p* < 0.05) difference in any available survival metric was observed between clusters. Stability: normalized mutual information (NMI) of the clusters discovered by repeated *k*-means clustering (for methods that use *k*-means clustering). Pathway activity: Number of cancer types for which a significant (Kruskal–Wallis *p* < 0.05) difference in the activity of any of 11 cancer-associated signaling pathways was observed between clusters. Data for pathway activity was available for 27 cancer types^16^. See Methods for details of calculations. Silhouette and stability are reported as mean ± standard deviation

Additionally, we applied CIMLR to four types of pediatric cancers using data from the TARGET initiative^13^. Remarkably, the clusters obtained by CIMLR present significant differences in overall survival for all four pediatric tumor types (Fig. 1b, Supplementary Table 3), exceeding the performance of other approaches (Table 1, Supplementary Data 3, Supplementary Data 4).

CIMLR learns weights for each data type instead of assigning equal importance to each. We note that the contributions of each data type, measured as the fraction of total kernel weight contributed by kernels based on that data type, are very different between cancers (Fig. 1b). While expression and methylation each contribute 30–50% of the kernel weight in almost all cancers, the contributions of point mutations and copy number are highly variable. We observe some association between these kernel weights and the C/M classification of cancers^14^, with M-type cancers, such as endometrial and colorectal cancers having high contributions from point mutations while copy number changes contribute more to subtyping of some C-type cancers, such as ovarian cancer. CIMLR can thus give us insight into which data types are most informative for subtyping in different cancers.

Finally, all other approaches except SNF proved impractically time-consuming and computationally intensive to run (on the order of days using 64 cores for a single configuration), while CIMLR takes minutes to run on a laptop for each cancer type. In summary, we find that multi-omic data integration using CIMLR is the most effective method for integrative subtyping based on technical performance, discovery of clinical and biological differences, and practical usability.

### Biological validation of CIMLR on lower-grade gliomas

Lower-grade (also called low-grade) gliomas are a well-studied example for genomic subtyping, which is why we chose it for validation of CIMLR via reproduction of known results. Three subtypes of lower-grade gliomas have been characterized^15^, based on *IDH1/2* point mutations and chromosome 1p/19q codeletion.

**Fig. 2:**
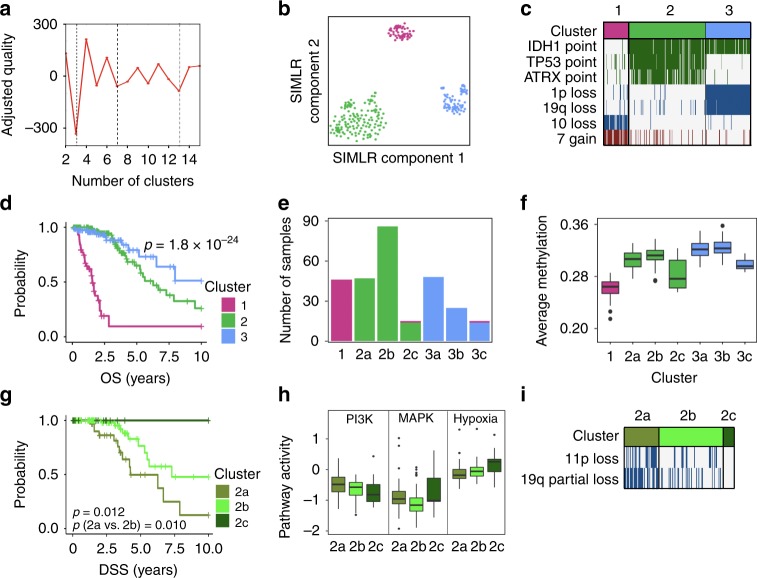
Validation of CIMLR on 282 lower-grade gliomas. **a** Plot of separation cost (*y*-axis) showing 3 as the best number of clusters and 7 and 13 as secondary peaks. **b** 2-D visualization of the three clusters. **c** Differences in molecular features between the three clusters. Each column represents a sample. Point mutations are represented by green bars, copy number losses by blue bars and gains by red bars. **d** Kaplan–Meier curves showing overall survival for the three clusters. **e** Bar plot showing the further separation of the three clusters into seven subclusters. **f** Boxplots showing average methylation beta value (*y*-axis) for patients belonging to each of the seven subclusters (*x*-axis). **g** Kaplan–Meier curves showing disease-specific survival for the three subclusters of cluster 2. **h** Boxplots showing the pathway activity^16^ for PI3K, MAPK and Hypoxia pathways, for patients belonging to the three subclusters of cluster 2. **i** Differences in molecular features between the three subclusters of cluster 2. Each column represents a patient and blue bars represent copy number losses. All *p*-values are calculated using the log-rank test. For all boxplots, the center line represents the median, box edges represent first and third quartiles, and whiskers represent ±1.5 IQR

CIMLR finds 3 to be the best number of clusters for lower-grade gliomas, with additional peaks at 7 and 13 (Fig. 2a). The three clusters show strong separation (Fig. 2b) and correspond to the known molecular subtypes. Cluster 1 is composed almost entirely of *IDH*-wild type samples with a loss of chromosome 10 and gain of chromosome 7. Cluster 2 (non-codel) is composed of mostly *IDH* mutant samples with additional point mutations in *TP53* and *ATRX*. Cluster 3 (codel) is composed of *IDH* mutant tumors with a chromosome 1p/19q codeletion (Fig. 2c). The *IDH*-wild type cluster has the worst overall and disease-specific survival, followed by the non-codel cluster (Fig. 2d).

A recent study^2^ comprising lower-grade gliomas and glioblastomas hinted at a finer classification of these tumors, finding a CIMP-low subgroup of *IDH* mutant non-codel tumors, with lower methylation and worse survival than the rest of the non-codel group. The codel group, on the other hand, was not divided further. To further characterize lower-grade gliomas, we investigated the results by CIMLR for seven clusters, which are near-perfect subsets of the three major clusters (Fig. 2e). We find that the codel and non-codel groups are divided into three subclusters each. In both groups, there are two CIMP-high subclusters and one CIMP-low subcluster (Fig. 2f).

We examined the subclusters of cluster 2. Subcluster 2c is characterized by reduced methylation (Fig. 2f), similar to the CIMP-low subgroup described previously^2^. The three subclusters have significantly different overall (log-rank *p* = 0.043) and disease-specific (log-rank *p* = 0.012) survival (Fig. 2g). Analysis of pathway activity scores^16^ showed that they have significantly different activity of the PI3K, MAPK, and hypoxia pathways (Fig. 2h). Further, subcluster 2a, which has the worst survival outcomes, is associated with more copy number changes than 2b or 2c; 68% of samples in 2a have a partial loss of 19q (19q13.31–13.43), unlike the complete-arm loss in the codel group. 57% have a loss of 11p (Fig. 2i), including the tumor suppressor *TRIM3*, which also showed reduced expression in the same samples. *TRIM3* loss has been associated with increased proliferation and stem cell-like properties of glioblastomas^17^.

Thus, CIMLR reproduces known molecular subtypes and also reveals novel subgroups within lower-grade gliomas. This provides empirical evidence that CIMLR can discover meaningful and robust biological subtypes using multi-omic data. We therefore evaluated the clusters found by CIMLR for all cancer types. To characterize the biological changes that lead to survival differences between clusters, we identified genetic alterations that were enriched in specific clusters, and used gene set enrichment analysis (GSEA) and PROGENy^16^ to identify cancer-related biological pathways that were activated differently between clusters. Below we present results for eight cancers where we obtain a significant difference in survival and improve over previous clustering studies.

### Liver hepatocellular carcinoma

**Fig. 3:**
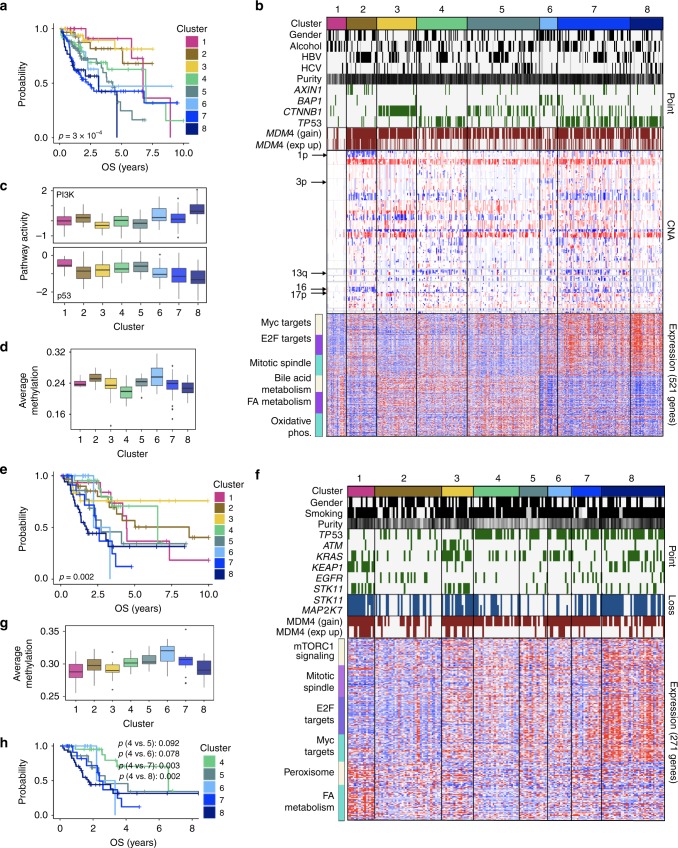
Liver hepatocellular carcinomas and lung adenocarcinomas. **a** Kaplan–Meier curves showing overall survival for eight clusters of liver hepatocellular carcinoma. **b** Selected clinical and molecular features that differentiate the eight clusters of liver hepatocellular carcinoma. Each column represents a patient. For gender, alcohol, HBV, and HCV, gray bars represent missing data. Black bars represent females, alcohol consumption, HBV or HCV infection. Tumor purity is shown along a white (low) to black (high) spectrum. Copy number alterations (CNA) and RNA expression are shown along a blue (low) to red (high) spectrum. **c** Boxplots showing pathway activities^16^ for PI3K and p53 pathways, for each of the eight clusters of liver hepatocellular carcinoma. **d** Boxplots showing average methylation beta value (*y*-axis) for patients belonging to each of the eight clusters of liver hepatocellular carcinoma (*x*-axis). **e** Kaplan–Meier curves showing overall survival for eight clusters of lung adenocarcinoma. **f** Selected clinical and molecular features that differentiate the eight clusters of lung adenocarcinoma. Each column represents a patient. For gender and smoking, gray bars represent missing data. Black bars represent females and smokers respectively. Tumor purity is shown along a white (low) to black (high) spectrum. RNA expression is shown along a blue (low) to red (high) spectrum. **g** Boxplots showing average methylation beta value (*y*-axis) for patients belonging to each of the eight clusters of lung adenocarcinoma. **h** Kaplan–Meier curves showing overall survival for the five clusters associated with *TP53* mutations in lung adenocarcinoma. All *p*-values are calculated using the log-rank test. For all boxplots, the center line represents the median, box edges represent first and third quartiles, and whiskers represent ±1.5 IQR

Hepatocellular carcinoma is associated with several risk factors including chronic hepatitis B virus (HBV) and hepatitis C virus (HCV) infection, and alcohol consumption. iCluster+ has been used to find three integrative subtypes^18^; however, there was no significant difference in patient outcomes, although some differences were seen in an external cohort that was tracked over a longer time. CIMLR separates 359 liver hepatocellular carcinomas into eight clusters, associated with significant differences in overall (Fig. 3a) and disease-specific survival within the cohort.

Clusters 1, 2, and 3 have relatively high overall and disease-specific survival. We do not observe any common point mutations or copy number changes in cluster 1 (Fig. 3b); however, this may be due to the low purity and higher immune infiltration of these tumors^19^. Cluster 2 is associated with HBV infection (60% samples) and Asian ethnicity. Although most of these tumors are wild-type for *TP53*, they show gain and increased expression of *MDM4*, which encodes a p53 repressor, and low p53 pathway activity^16^ (Fig. 3c). This cluster has a universal loss on chromosome 1p including the succinate dehydrogenase gene *SDHB*, accompanied by reduced *SDHB* expression. Reduced SDHB blocks respiration leading to a metabolic shift toward glycolysis; the accumulation of succinate also inhibits demethylases leading to a CIMP (high methylation) phenotype^20^, which we observe in this cluster (Fig. 3d). This cluster also displays losses on chromosome 16, including the tumor suppressors *CYLD* and *TSC2*, and the DNA repair gene *PALB2*, as well as reduced expression of all three. It is also enriched (28% samples) for mutations in *AXIN1*, a tumor suppressor gene that regulates the Wnt signaling pathway. GSEA shows that this cluster is enriched for tumors with reduced expression of genes for oxidative phosphorylation and the G1/S checkpoint.

Cluster 3 is enriched for mutations in *CTNNB1* (beta-catenin). While *CTNNB1* mutations are also common in other clusters, the tumors in cluster 3 also display high expression of *GLUL* (Glutamine synthase), a well-characterized target of beta-catenin^21^, suggesting that beta-catenin activation leads to glutamine synthesis and cellular proliferation in these tumors.

Patients in cluster 6 are more likely to be female (Fisher’s test *p* = 0.001), non-drinkers, and do not have HBV or HCV infection. This cluster is enriched for mutations in the tumor suppressor *BAP1*, which is involved in chromatin remodeling as well as double-strand break repair (42% samples). 63% of samples also share a loss of *BAP1* on 3p, along with reduced expression. These tumors have high DNA methylation, a phenotype previously associated with *BAP1* mutations in renal cancers^22^, and frequently lack the 8p loss/8q gain that is seen in other clusters. In addition, they show strongly reduced expression of genes for normal hepatocyte functions, such as bile acid metabolism, fatty acid metabolism, xenobiotic metabolism, and coagulation.

Clusters 4, 7, and 8 are associated with *TP53* point mutations, as well as losses on 13q (*RB1*) and 17p (*MAP2K4, TP53*). However, clusters 7 and 8 have significantly worse overall survival than cluster 4 (log-rank *p* = 0.045 and *p* = 0.036 respectively). Both show increased expression of Myc and E2F target genes, as well as genes involved in mTORC1 signaling and the mitotic spindle. In addition, cluster 8 shows reduced expression of genes involved in normal hepatocyte function (as seen in cluster 6), higher immune infiltration and macrovascular invasion. p53 and PI3K pathway activities^16^ are significantly associated with the clusters (*p* < 10^−12^ for both, Kruskal–Wallis test), with cluster 8 showing the lowest p53 activity and highest PI3K activity.

### Lung adenocarcinoma

Lung adenocarcinoma, often caused by smoking, is the leading cause of cancer death globally. Previous studies identified transcriptional^23^ and histological^24^ subtypes, as well as six integrated clusters^9^, which, however, showed no significant association with patient survival. CIMLR separates 188 lung adenocarcinomas into eight clusters, significantly associated with overall and disease-specific survival (Fig. 3e).

Clusters 1–3 are predominantly wild-type for *TP53*, whereas the remaining clusters (4–8) are associated with *TP53* mutations (Fig. 3f). In general, the *TP53* mutant clusters are associated with worse survival outcomes; the exception is cluster 4, which has significantly better overall and disease-specific survival outcomes than the other *TP53*-mutant clusters, comparable to clusters 1–3 (Fig. 3h).

Cluster 1 is characterized by loss of 19p, including the tumor suppressor *STK11*; this is associated with reduced *STK11* expression. It is enriched for point mutations in *STK11* and *KEAP1*, as well as high expression of *CCND3* (cyclin D3), the transcriptional regulator *MUC1*, the Wnt pathway activator *PYGO2* and the p53 inhibitor *MDM4*. In addition, it shows low DNA methylation (Fig. 3g), high expression of genes for fatty acid metabolism and peroxisome function, and low expression of genes involved in apoptosis and the G2/M checkpoint.

Cluster 3, like cluster 1, has low methylation, and is associated with *STK11* loss and point mutations. In addition, it is enriched for point mutations in *ATM* and *KRAS*. It has a gain on 14q and losses on 1p, 21q (*BTG3, PRMT2, HMGN1*), and 15q (*FAN1*), as well as reduced expression of those genes. This cluster is associated with high expression of the oncogene *KIT* and the chromatin modifiers *CHD7* and *SUDS3*, as well as high expression of genes involved in membrane fusion and budding, and the unfolded protein response.

Among the five *TP53*-mutated clusters, cluster 4, which has the best outcomes, has a loss on chromosome 15, as well as low expression of genes involved in DNA repair and oxidative phosphorylation. Interestingly, tumors in this cluster are enriched for splice-site mutations in *TP53* (20% of *TP53* mutations in this cluster), and mutations in exon 4 of *TP53* (25%), whereas the other clusters are dominated by missense and nonsense mutations in exons 5–10. However, neither exon 4 nor splice site mutations, nor both combined, were significantly associated with survival in this dataset.

Cluster 6 is a small cluster of 14 samples, associated with high DNA methylation, *KRAS* mutations and increased expression of the chromatin remodeling factor *SATB2*. Finally, cluster 8 shows the worst overall survival; it is associated with males, a high rate of point mutations, and low methylation. In addition to *TP53* point mutations, it has a loss of 19p (*MAP2K7, STK11*; >50% samples also have reduced expression of both these genes), high expression of the RNA methyltransferase *NSUN2*, and high expression of genes for the mitotic spindle, Myc targets, E2F targets, and mTORC1 signaling.

### Head and neck squamous cell carcinoma

Head and neck squamous cell carcinomas (HNSCCs) are very heterogeneous in etiology and phenotype. They are stratified by tumor site, stage and histology, and human papilloma virus (HPV) has been associated with better patient outcomes^25^.

**Fig. 4:**
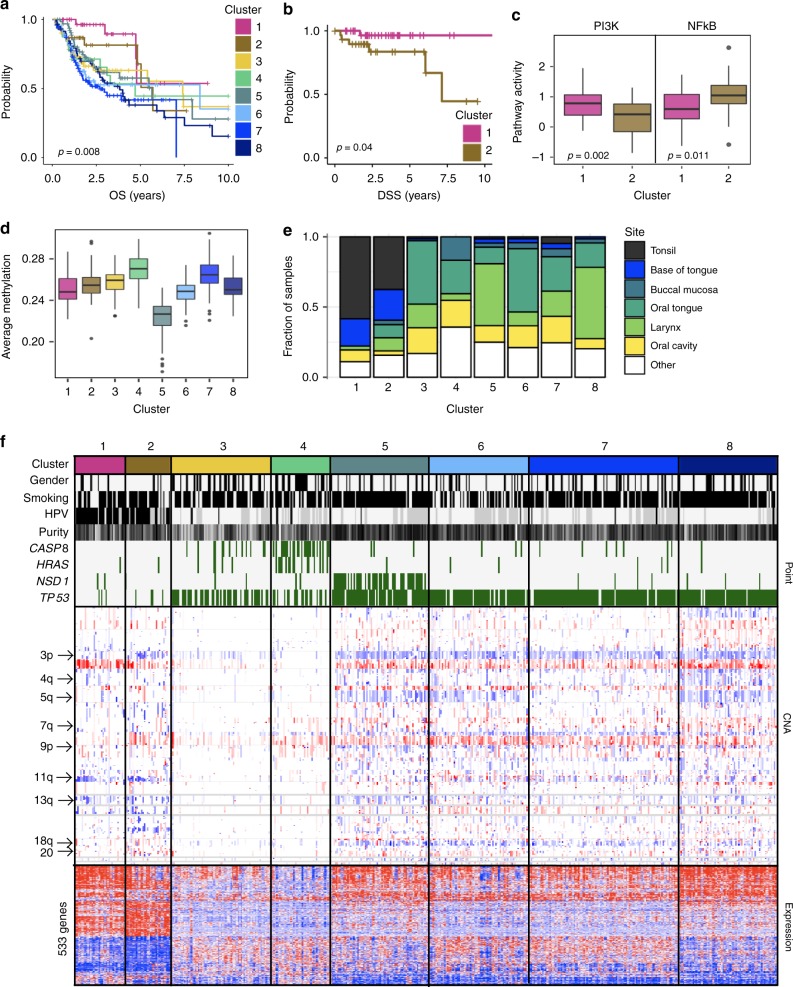
Head and neck squamous cell carcinomas. **a** Kaplan–Meier curves showing overall survival for the eight clusters of head and neck squamous cell carcinomas. *p*-Value was calculated by the log-rank test. **b** Kaplan–Meier curves showing disease-specific survival for clusters 1 and 2. *p*-Value was calculated by the log-rank test. **c** Boxplots showing pathway activities^16^ for PI3K and NFkB pathways in clusters 1 and 2. *p*-Values were calculated using the Wilcoxon test (one-sided). **d** Boxplot showing average methylation beta value (*y*-axis) for patients belonging to each of the eight clusters. **e** Bar chart showing the fraction of tumors (*y*-axis) in each cluster (*x*-axis) according to the primary site of the tumor. **f** Selected clinical and molecular features that differentiate the eight clusters. Each column represents a patient. For gender, smoking and HPV, gray bars represent missing data. Black bars represent females, smokers, and HPV infection. Tumor purity is shown along a white (low) to black (high) spectrum. Copy number alterations (CNA) and RNA expression are shown along a blue (low) to red (high) spectrum. For all boxplots, the center line represents the median, box edges represent first and third quartiles, and whiskers represent ± 1.5 IQR

We separate 495 HNSCCs into eight subtypes, which are significantly associated with overall (Fig. 4a) and disease-specific survival. Tumors in clusters 1 and 2 are predominantly HPV+ and *TP53* wild-type (Fig. 4f). They are found mostly in the tonsils and base of tongue (Fig. 4e), and share a loss on 11q. These HPV+ clusters have significantly higher overall (log-rank *p* = 6.1 × 10^−3^) and disease-specific (log-rank *p* = 2.1 × 10^−3^) survival than the remaining clusters.

However, cluster 2 has significantly worse disease-specific survival than cluster 1 (Fig. 4b), and differs in gene expression. While cluster 1 is associated with high expression of 59 genes including the oncogenes *DEK* and *PIK3CA*, cluster 2 shows elevated *NFKB2* expression, and reduced expression of *CDH1* and *MAP2K4*. GSEA shows that tumors in cluster 2 also show reduced expression of genes involved in PI3K/AKT/mTOR signaling. Consistent with these features, cluster 2 has significantly higher NFkB pathway activity than cluster 1, whereas cluster 1 has significantly higher activity of the PI3K pathway^16^ (Fig. 4c). Finally, 62% of the samples in cluster 2 have loss of chromosome 3p, compared to 27% of cluster 1. 3p loss, occurring jointly with HPV status, has been associated with worse prognosis^26^.

The remaining six clusters are HPV-negative and tend to have point mutations in *TP53*. We do not find significant survival differences within this group, although they differ in molecular features. Cluster 4 has high DNA methylation (Fig. 4d) and is enriched for females and nonsmokers. This cluster lacks the common 3q gain but is enriched for point mutations in *CASP8*, *FAT1*, *HRAS*, *HUWE1* and the histone methyltransferase *KMT2B*. Clusters 5, 6, 7 and 8 all have high genomic instability. However, cluster 5 is associated with loss of function of the histone methyltransferase NSD1; 68% of the samples have a point mutation in *NSD1* while an additional 6% have homozygous deletion of this gene. Tumors in this cluster are hypomethylated, a pattern previously associated with *NSD1* loss^27^, and have losses on 13q and 9p.

Cluster 8, which has the highest genomic instability, is enriched for a gain on 7q (including *SMURF1*; also increased in expression) and a loss on 4q, and high expression of 35 genes including *PIK3CA* and the transcriptional regulator *YEATS2*, as well as low expression of the ubiquitin-conjugating enzyme *UBE2D3*, a phenotype linked to cell cycle progression, reduced apoptosis, and telomere stability^28^.

### Clear cell renal cell carcinoma

Clear cell renal cell carcinomas are the most common kidney cancers. Common genetic alterations include mutations in *VHL* and *PBRM1*, 3p loss and 5q gain. Using 260 clear cell renal cell carcinoma samples, CIMLR finds two cluster number peaks, at 4 and 10.

**Fig. 5:**
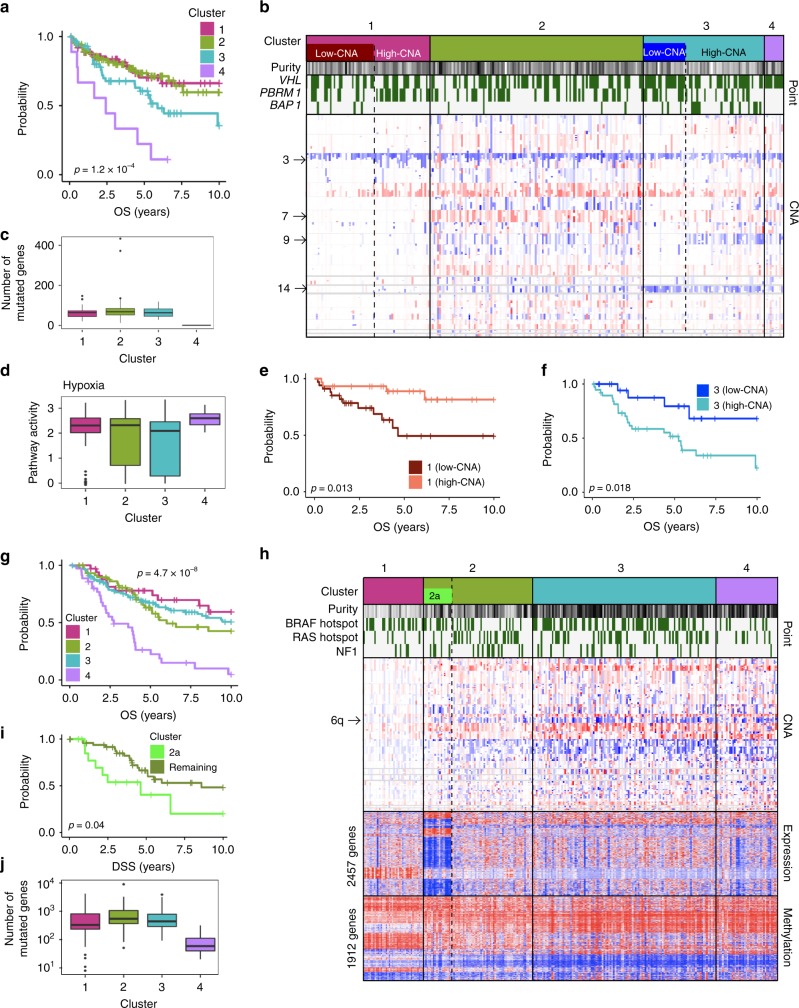
Clear cell renal cell carcinomas and cutaneous melanomas **a** Kaplan–Meier curves showing overall survival for four clusters of clear cell renal cell carcinoma. **b** Selected clinical and molecular features that differentiate the four clusters of clear cell renal cell carcinoma. Each column represents a patient. Tumor purity is shown along a white (low) to black (high) spectrum. Copy number alterations (CNA) are shown along a blue (low) to red (high) spectrum. **c** Boxplots showing the number of mutated genes in patients belonging to each of the four clusters of clear cell renal cell carcinoma. **d** Boxplots showing pathway activity^16^ for hypoxia in the four clusters of clear cell renal cell carcinoma. **e** Kaplan–Meier curves showing overall survival for subsets of cluster 1. **f** Kaplan–Meier curves showing overall survival for subsets of cluster 3. **g** Kaplan–Meier curves showing overall survival for the four clusters of cutaneous melanoma. **h** Selected clinical and molecular features that differentiate the four clusters of cutaneous melanoma. Each column represents a patient. Tumor purity is shown along a white (low) to black (high) spectrum. Copy number alterations (CNA), RNA expression and promoter methylation are shown along a blue (low) to red (high) spectrum. **i** Kaplan–Meier curves showing disease-specific survival for patients belonging to cluster 2a and the remaining patients belonging to cluster 2. **j** Boxplots showing the number of mutated genes in patients belonging to the four clusters of cutaneous melanoma. All *p*-values are calculated using the log-rank test. For all boxplots, the center line represents the median, box edges represent first and third quartiles, and whiskers represent ±1.5 IQR

The four major clusters show significant differences in overall (Fig. 5a) and disease-specific survival. Clusters 1 and 2 have the best survival outcomes; cluster 2 shows higher genomic instability, particularly a gain on chromosome 7 (Fig. 5b). Cluster 3 has significantly worse survival outcomes than clusters 1 and 2 (log-rank *p* = 0.022), and is characterized by a loss on chromosome 14, including the tumor suppressor *WDR20*, which also shows reduced expression; this gene suppresses growth and apoptosis in renal cancer cell lines^29^. Cluster 4 is a small cluster with significantly worse overall and disease-specific survival than the other three clusters. These tumors have only one point mutation each in coding regions (mostly in *VHL*) (Fig. 5c), high hypoxia pathway activity^16^ (Fig. 5d), low expression of the chromatin modifier *SETD2*, and high expression of the helicase *DDX11*, which is overexpressed in multiple cancers and associated with proliferation and survival in melanomas^30^.

On examining the split into 10 clusters, we found that several of these were subsets of the four major clusters. Interestingly, a subset of cluster 1, characterized by fewer copy number alterations, shows significantly worse overall (Fig. 5e) and disease-specific (log-rank *p* = 0.013) survival than the rest of cluster 1. We also identified a subcluster within cluster 3 which shows significantly better overall (Fig. 5f), and disease-specific (log-rank *p* = 0.004) survival than the rest of cluster 3. This low-CNA group lacks a loss on chromosome 9 (including *NOTCH1* and the tumor suppressor *TSC1*) which is present in the rest of the cluster. Instead, it has reduced expression of several genes involved in DNA repair (*CCNK, MLH3, MTA1, APEX1*).

### Cutaneous melanoma

Cutaneous melanoma is particularly difficult to subtype since it frequently has a high mutational burden. These tumors have been classified on the basis of common mutations; however, this classification is not predictive of patient outcomes^10^. Instead, CIMLR separates 262 cutaneous melanomas into four clusters significantly associated with overall and disease-specific survival, and a second-best split at 10. Clusters 1, 2, and 3 are not significantly different in terms of survival; however, cluster 4 has significantly worse overall and disease-specific survival than all other clusters (Fig. 5g).

Cluster 1 is characterized by relatively low purity and high immune cell infiltration (Fig. 5h). Cluster 2 has high expression of genes involved in mTORC1 signaling and DNA synthesis. While the outcomes for these patients are similar, on examining the split into 10 clusters, we identify a subcluster (2a) that has significantly worse disease-specific survival than the rest of cluster 2, and is in fact comparable to cluster 4 (Fig. 5i). This subcluster has a distinctive expression pattern, which does not appear to be driven by copy number. This includes high expression of genes for autophagy, organelle fusion and protein transport, and low expression of genes involved in the G2/M checkpoint, splicing, DNA repair, RNA metabolism, and chromatin remodeling.

Cluster 3 is differentiated by a loss of 47 genes on chromosome 6q and by reduced expression of genes involved in oxidative phosphorylation. Finally, cluster 4 is distinguished by a low point mutation burden (~80 coding mutations per tumor) (Fig. 5j), as well as high expression of three genes (*BTBD9, CDYL, TFAP2A*) and high methylation at 100 promoters.

### Breast cancer

**Fig. 6:**
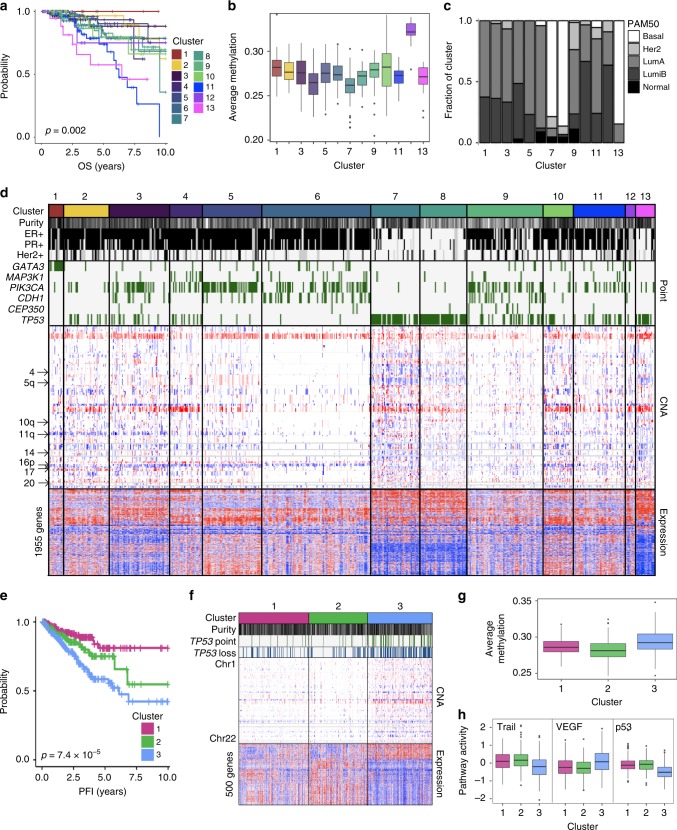
Breast and prostate cancers. **a** Kaplan–Meier curves showing overall survival for the 13 clusters of breast cancer. **b** Boxplots showing average methylation beta value (*y*-axis) for patients belonging to each of the 13 clusters of breast cancer. **c** Bar plot showing distribution of *PAM50* subtypes within each of the 13 clusters of breast cancer. LumA: Luminal A, LumB: Luminal B. **d** Selected clinical and molecular features differentiating the 13 clusters of breast cancer. Each column represents a patient. For ER+, PR+, and HER2+, gray bars represent missing data, black bars represent hormone receptor-positive tumors, and white bars represent hormone receptor-negative tumors. Tumor purity is shown along a white (low) to black (high) spectrum. Copy number alterations (CNA) and RNA expression are shown along a blue (low) to red (high) spectrum. **e** Kaplan–Meier curves showing progression-free interval for the three clusters of prostate cancer. **f** Selected clinical and molecular features that differ between the three clusters of prostate cancer. Each column represents a patient. Tumor purity is shown along a white (low) to black (high) spectrum. Copy number alterations (CNA) and RNA expression are shown along a blue (low) to red (high) spectrum. **g** Boxplots showing average methylation beta value (*y*-axis) for patients belonging to each of the three clusters of prostate cancer. **h** Boxplots showing pathway activities^16^ for Trail, VEGF, and p53 pathways in the three clusters of prostate cancer. All *p*-values are calculated using the log-rank test. For all boxplots, the center line represents the median, box edges represent first and third quartiles, and whiskers represent ±1.5 IQR

Breast cancers are frequently classified by intrinsic subtypes^1^ or by ER, PR, and HER2 receptors. Another classification, IntClust^31^, comprises 10 clusters based on copy number and expression. CIMLR separates 663 breast tumors into 13 clusters, which are significantly different in overall (Fig. 6a) and disease-specific survival. Ten of these are predominantly ER+ while three are predominantly triple-negative (Fig. 6c and d). There are significant differences in survival within each group and we examine them separately.

Among the predominantly ER+ clusters, Clusters 1, 2, and 3 share a loss on 11q that includes *SDHD, ATM, ARHGEF12*, and *EI24*. Cluster 1 has the best survival outcomes and is enriched for point mutations in *GATA3* (71% samples). On the other hand, clusters 2 and 3 are enriched for HER2+ tumors and have gains on 17q and 20, as well as a loss on 17p, including *RPA1*, which encodes an ssDNA-stabilizing protein. In addition, Cluster 3 has a gain on 16p, which is shared by clusters 4 and 5, and is enriched for patients with African ancestry. Cluster 12 is a small cluster of 11 patients that display global DNA hypermethylation (Fig. 6b) and high expression of genes for telomere maintenance.

Cluster 11 has significantly worse survival outcomes than the other predominantly ER+ clusters (except clusters 4 and 12 which have small sample size). This cluster is differentiated from the other ER+ clusters by methylation (it shows significant hypermethylation of 128 promoters and hypomethylation of 186 promoters) and by higher MAPK pathway activity^16^. It has low expression of *NEURL4*; this gene encodes a regulator of centrosome organization and its depletion results in mitotic abnormalities in human cell lines^32^. This cluster also has high expression of *TAF2*, encoding a transcriptional regulator associated with dedifferentiation and proliferation in ovarian cancer^33^.

Three clusters—7, 8, and 13—are dominated by triple-negative tumors. All three are characterized by *TP53* mutations and similar patterns of copy number changes, expression and methylation, and clusters 8 and 13 are enriched for patients with African ancestry. However, cluster 13 has significantly worse survival outcomes than clusters 7 and 8 (log-rank *p* = 0.031 and *p* = 0.045, respectively, for disease-specific survival). This cluster is differentiated from clusters 7 and 8 by elevated expression of 230 genes and reduced expression of 442 genes including the tumor suppressors *APC, CREB1, NCOR1*, and *NUP98*. In addition, it has significantly higher VEGF activity than clusters 7 and 8^16^, suggesting higher angiogenesis. It is notable that the six ER+ tumors in this cluster share the expression changes described above.

### Prostate cancer

CIMLR finds three clusters in a dataset of 490 primary prostate tumors. For this cancer, we do not consider overall survival as very few patients died during the 10-year follow-up period; instead, we observe significant differences in progression-free interval (Fig. 6e) and disease-free interval.

Clusters 1 and 2 differ primarily in expression, as well as methylation of 13 promoters. Cluster 3 has significantly worse outcomes than both clusters 1 and 2. It is characterized by high genomic instability (Fig. 6f), including loss of the tumor suppressor *TRIM35* on chromosome 8, reduced expression of the tumor suppressor *RHOBTB2*, and high promoter methylation (Fig. 6g). It also has higher activity of the VEGF pathway and lower activity of the apoptotic Trail pathway (Fig. 6h). 63% of the samples in this cluster have p53 mutation and/or loss, and the cluster has lower p53 pathway activity than the others^16^.

Apart from the detailed analysis for eight cancers presented above, we provide clustering results for all cancers (Supplementary Data 7, Supplementary Data 8), and summarize the main features of the clusters for each cancer (Supplementary Note 2, Supplementary Figs. 1–36).

### CIMLR validation on unseen data

For five cancers, we were able to find sufficient additional multi-omic data to validate our biological findings. For lower-grade glioma, clear cell renal cell carcinoma, cutaneous melanoma and breast cancer, we obtained new unseen samples recently released by TCGA^34^. For prostate cancer, we used a non-TCGA dataset^35^. For each cancer, we classified the tumors in the new dataset into high-risk and low-risk groups based on the original clusters, using genomic features that differed significantly between clusters. We then assessed whether the survival differences discovered in the original dataset were reproduced in the test data (Table 2).

**Table 2:** Validation of CIMLR findings on unseen data

Cancer	Comparison	Samples (discovery)	*p*-value (discovery)	CI (discovery)	Samples (validation)	*p*-value (validation)	CI (validation)
Lower-grade glioma	Cluster 1 vs. rest	282	2.0 × 10^−25^	0.76	226	1.8 × 10^−12^	0.75
Lower-grade glioma	Cluster 2a vs. rest of cluster 2	147	0.035	0.63	108	0.028	0.69
Clear cell renal cell carcinoma	Cluster 3 vs. clusters 1 + 2	260	0.022	0.58	138	0.019	0.63
Cutaneous melanoma	Cluster 4 vs. rest	262	2.3 × 10^−9^	0.59	103	0.036	0.59
Breast cancer (excluding triple-negative)	Cluster 11 vs. rest	536	3.2 × 10^−6^	0.59	347	1.6 × 10^−5^	0.59
Prostate cancer	Cluster 3 vs. rest	490	1.2 × 10^−3^	0.65	118	0.011	0.62

*p*-values and CI were calculated for overall survival, except for prostate cancer, in which case disease-free interval (DFI) was used. *p*-values were calculated using the log-rank test

For example, for lower-grade gliomas, we classified 226 tumors into the three major clusters found by CIMLR and validated that Cluster 1 has lower survival than the rest of the population. We then selected the tumors predicted to belong to cluster 2 and classified them into high-risk (subcluster 2a) and low-risk (subclusters 2b + 2c) groups. Our novel finding that tumors of subcluster 2a have worse overall survival outcomes than the rest of this cluster was validated in this dataset. For clear cell renal cell carcinoma, we classified 138 samples as high (cluster 4), intermediate (cluster 3) or low-risk (cluster 1 + cluster 2). Only two samples in the validation set were classified into the high-risk group (cluster 4). However, samples classified as intermediate risk (cluster 3) had significantly worse overall survival than samples classified as low-risk. Similarly, we validated worse survival outcomes for cluster 4 in cutaneous melanoma, cluster 11 for non-triple negative breast cancers, and cluster 3 for prostate cancer, in their respective external datasets. This analysis demonstrates that the survival differences discovered by CIMLR are reproducible and potentially clinically useful.

Further, in order to ask whether multi-omic subtyping results in prognostic value beyond clinical variables commonly employed to predict survival, we also evaluated the prognostic value of the CIMLR clusters using Cox proportional hazard regression in both the discovery and validation sets. We found that CIMLR clusters were associated with significant hazard ratios and high concordance index (CI)^36^ values. We also note that CI values were similar in each of the matched discovery and validation sets. Moreover, in 11 cancers in the discovery sets, as well as 3 of the external validation sets, CIMLR clusters were associated with significant hazard even after adjusting for common clinical variables (Supplementary Note 1, Supplementary Data 5, Supplementary Data 6). These results provide strong evidence that multi-omic subtyping using CIMLR offers significant prognostic value beyond that of commonly used clinical features.

## Discussion

The importance of integrative cancer subtyping has been recognized for several years, and multiple algorithms have been developed to exploit the growing amount of available multidimensional data^4–8^. CIMLR addresses many of the weaknesses of current integrative subtyping algorithms, outperforming all tested methods in terms of cluster separation and stability. Furthermore, most of the alternative algorithms proved impractically time-consuming and computationally intensive to run on the considerable volume of data analyzed in this study. As the amount of genomic data is growing rapidly and more types of data are becoming available (such as gene fusions, structural variants, proteomes, miRNA, and ATAC-Seq), efficient methods are essential. Of the available methods, CIMLR is not only superior in terms of performance but is also capable of practically scaling to large-scale analyses with many more data types. We therefore anticipate significant use of this method in the future.

The subtyping achieved by CIMLR demonstrates both biological and clinical relevance. The discovered clusters exhibit significant differences in the activity of oncogenic and tumor suppressor pathways, and show significant differences in patient survival in 27 of 36 cancer types. The discovered subtypes provide valuable biological insights and are more predictive of survival than other commonly used classifications. For example, for thymomas the CIMLR subtypes perform better at predicting survival than histological classification (Supplementary Note 2), while the CIMLR subtypes of cutaneous melanoma are much better at predicting survival than classification based on BRAF, RAS, and NF1 mutations.

For head and neck squamous cell carcinomas, we separate HPV+ tumors into two groups with significantly different survival outcomes and pathway activity; a previous subtyping attempt using gene expression did not predict survival^37^. Similarly, in clear cell renal carcinomas, where chromosome 14 loss has been associated with poor prognosis^38^, we not only find a cluster enriched for chromosome 14 loss but show that this is divided into two subclusters only one of which is associated with poor prognosis. In breast cancer, we separate triple-negative cancers for the first time into three clusters, one of which is considerably more aggressive than the others and is associated with reduced expression of several tumor suppressor genes. Finally, we validate several of the survival differences discovered by CIMLR in external datasets, showing that CIMLR discovers molecular subtypes associated with robust, reproducible clinical outcomes.

Our results demonstrate the value of machine learning-based multi-omic subtyping in cancer, and the need for more effective and practically usable algorithms. As more data becomes available, the predictive power of CIMLR and related approaches will continue to increase. We expect that subtyping will be useful in stratifying patients for prediction of outcomes and drug response to improve personalized treatment. In addition, our work can be used as a resource for future studies aimed at understanding the biology and evolution of these cancers.

## Methods

### Data preprocessing

We considered all the 32 cancer types studied by TCGA and collected, for each of them, multi-omic data comprising somatic point mutations (as TCGA Mutation Annotation Format files and converted to binary values, 0 to report absence of a mutation in a gene and 1 to report its presence), copy number alterations (log_2_ ratios between tumor and normal tissue), methylation (beta-values, i.e. continuous values between 0 and 1), and expression (*z*-scores normalized to normal tissue or to tumors with diploid genomes). For the TARGET data, we considered four pediatric tumors: acute myeloid leukemia, Wilms tumor, neuroblastoma, and osteosarcoma. For each of them we collected multi-omic data comprising copy number alterations (log_2_ ratios between tumor and normal tissue), methylation, and RNA expression.

Moreover, we removed extreme values for both copy number log_2_ ratios and expression *z*-scores by setting values greater than 10 to 10 and values lower than −10 to −10. We refer to TCGA guidelines for a detailed description of the data obtained from the consortium at the following Website: https://wiki.nci.nih.gov/display/TCGA. All the considered data were within the Open Access Data Tier.

Each data type was modeled as a matrix *N* × *M*, where *N* represents the samples, i.e., the patients, and *M* a set of genes. Each data matrix was normalized so that values ranged between 0 and 1.

### CIMLR

We extended the original implementation of SIMLR^11^ to use multi-omic data. The version of SIMLR adopted here is the default version rather than the large-scale version which leaves out the similarity enhancement by diffusion step.

The original method^11^ takes as input a dataset where rows are samples and columns are genes, and constructs a set of Gaussian kernels for the dataset by fitting multiple hyperparameters. Gaussian kernels are defined as follows:

1 $$K\left( {x_i,x_j} \right) = \frac{1}{{{\it{\epsilon }}_{ij}\sqrt {2\pi } }}{\mathrm{exp}}\left( { - \frac{{\left\Vert {x_i - x_j} \right\Vert_2^2}}{{2{\it{\epsilon }}_{ij}^2}}} \right)$$

where *x*_*i*_ and *x*_*j*_ denote the *i*th and *j*th rows (i.e., samples) of the input data and ${\it{\epsilon }}_{ij}^2$ is the variance.

For CIMLR, we represented each of the data types as a patient × gene matrix. We then performed the above procedure for each data type independently, to obtain a set of 55 gaussian kernels with different variance per data type. The number of 55 kernels per data type was empirically derived (Supplementary Table 4, Supplementary Fig. 37).

Then, we solved the same optimization problem described in SIMLR^11^, but considering the Gaussian kernels for all the data types together to build one patient × patient similarity matrix. This optimization problem is defined as follows:

2 $$\begin{array}{l}{\mathrm{minimize}}_{S,L,w} - \mathop{\sum}\limits_{i,j,l} {w_lK_l} \left( {x_i,x_j} \right)S_{ij} + \beta\left\Vert {S} \right\Vert_{\mathrm{F}}^2 + \gamma \,tr\left( {L^T\left( {I_N - S} \right)L} \right) + \rho \mathop {\sum}\limits_l {w_l} {\mathrm{log}}\,w_l\\ {{{\rm{subject}}\,{\rm{to}}}}\,L^TL = I_C,\mathop{\sum}\limits_l {w_l = 1} ,w_l \ge 0,\mathop {\sum}\limits_j {S_{ij} = 1} ,{\mathrm {and}}\,S_{ij} \ge 0.\end{array}$$

Here, *N* is the number of patients, *C* is the number of clusters, *i* is the row (sample) index, *j* is the column (gene) index, and *l* is the kernel index which ranges from 1 to (55 ×  number of data types). In the optimization framework, we solve for *S*, i.e., the *N* × *N* similarities matrix; moreover, *w*_*l*_ represents the weight of each Gaussian kernel, *I*_*N*_ and *I*_*C*_ are *N* × *N* and *C* × *C* identity matrices, *β* and *γ* are non-negative tuning parameters, $\left\| S \right\|_F$ is the Frobenius norm of *S,* and *L* is an auxiliary low-dimensional matrix enforcing the low rank constraint on *S*.

### Number of clusters

We also extended the method to estimate the best number of clusters presented in SIMLR^11^ based on separation cost to multi-omics. For a given value of *C*, we aim at finding an indication matrix *Z*(*R*) = *XR*, with *X* being the matrix of the top eigenvectors of the similarity Laplacian and *R* a rotation matrix. Let:

3 $$[M(R)]_i = {\mathrm {max}}_j\left[ {Z\left(R \right)} \right]_{i,j}$$

Then, we can define the following cost function to be minimized:

4 $$J\left(R\right) = \mathop {\sum}\limits_{i,j} {\frac{{[Z(R)]_{i,j}^2}}{{[M(R)]_i^2}}}$$

The best number of clusters is the one for which we obtain the largest drop in the value of *J(R)* over the set of values we consider for *C*.

We considered 2–15 clusters for the cancer types where we had at least 150 samples, or a maximum of *N*/10 clusters (where *N* is the number of samples) for smaller datasets.

### Survival analysis

We used four outcome metrics provided by TCGA: overall survival (OS), disease-specific survival (DSS), progression-free interval (PFI) and disease-free interval (DFI), over a time interval of 10 years. For OS, we censored data points corresponding to patients who died within 30 days or were over the age of 80 at the beginning of the observation period. For TARGET, we only considered OS data, censored in the same way as for TCGA. Clusters with only one sample were removed prior to survival analysis. Associations between subtypes and outcome were then calculated by Kaplan–Meier analysis using a log-rank test.

Cox regression analysis was performed to estimate hazard ratios associated with individual clusters and to test whether significant associations between clusters and survival outcomes remained after adjusting for common clinical features. Univariate Cox regression was used to select significant (two-sided Wald test *p* < 0.1) clinical features which were then included along with CIMLR clusters in a multivariate Cox regression model. Patient age, gender, race, ethnicity, tumor stage, and grade were taken into account where data was available. For prostate cancer, Gleason score was taken into account. Five cancers with an insufficient number of events to fit the Cox regression model were excluded from this analysis.

Survival analysis was carried out using the survival 2.41–3R package.

### Significant feature selection

Molecular features significantly enriched in each cluster were selected as follows. For each cluster, we carried out a hypergeometric test for enrichment of point mutations in each gene. We selected point mutations with an FDR-adjusted *p*-value of less than 0.05.

To select genes significantly enriched for copy number alterations, we obtained GISTIC thresholded copy number data for each sample from TCGA. We considered a value ≥1 to represent gain of the gene and ≤−1 to be loss of the gene. For each cluster, we used a hypergeometric test to assess whether the cluster was significantly enriched for either loss or gain of the gene, and selected genes with an FDR-adjusted *p*-value less than 0.05. For additional stringency and to select the features that were most representative of an individual cluster, we further selected only those genes that were altered in at least 2/3 of the samples in the cluster and <1/3 of the samples in at least one other cluster.

To select expression changes that were significantly enriched within a cluster, we considered a gene to be over-expressed when the *z*-score was ≥1, and under-expressed if the *z*-score was ≤−1. For each cluster, we selected enriched genes using the same criteria as for copy number.

For methylation, we considered a gene to be highly methylated when the beta-value was ≥0.75 and unmethylated when the beta-value was ≤0.25. For each cluster, we selected genes enriched for high or low methylation using the same criteria as for copy number.

### Classification of unseen data

To classify previously unseen samples into the CIMLR clusters, we used random forest classifiers. Features were ranked on the basis of the hypergeometric test described above and the threshold for selecting the most significant features was tuned to obtain high (>80%) out-of-bag classification accuracy on the discovery set. We used the ranger version 0.9.0 and caret version 6.0-79 R packages to train random forests and classify unseen samples. For all cancers other than prostate cancer, all four input data types were used for classification. For prostate cancer, only expression and copy number data were available for the validation set.

### Pathway analysis and immune cell infiltration

GSEA was performed on each cluster using the method of Segal et al.^39^. Gene sets (GO, Cancer Hallmarks, KEGG, Reactome) were obtained from mSigDB^40^. PROGENy pathway activity scores for 11 signaling pathways in TCGA patients were obtained from Schubert et al.^16^. Estimates of tumor immune infiltration were obtained from Li et al.^19^. All statistical analyses were carried out in R version 3.3.3.

### Code availability

 CIMLR is available for download at https://github.com/danro9685/CIMLR. Both R and Matlab implementations are available. The Matlab version was used in this paper.

## Electronic supplementary material

Supplementary Information

Description of Additional Supplementary Files

Supplementary Data 1

Supplementary Data 2

Supplementary Data 3

Supplementary Data 4

Supplementary Data 5

Supplementary Data 6

Supplementary Data 7

Supplementary Data 8

Reporting Summary

## Data Availability

The authors confirm that all relevant data generated in this study are included in the article and/or its supplementary information files.
