# Supplementary material for: Multi-omic tumor data reveal diversity of molecular mechanisms that correlate with survival
Source: Nat Commun. 2018 Oct 26;9:4453. doi: 10.1038/s41467-018-06921-8 (PMC6203719; doi:10.1038/s41467-018-06921-8)
Supplement: Supplementary file 1 — Supplementary Information [file 41467_2018_6921_MOESM1_ESM.pdf]

Supplementary information

Multi-omic tumor data reveal diversity of molecular mechanisms that correlate with survival

Ramazzotti et al.

Supplementary Note 1: Prognostic value of CIMLR clusters.

In order to ask whether multi-omic subtyping results in prognostic value beyond clinical variables commonly employed to predict survival, we evaluated the prognostic value of the clusters using Cox proportional hazard regression.

For each of the 23 TCGA cancers for which the CIMLR clusters showed significant association with survival by the log-rank test, we calculated the hazard ratio and associated 95% confidence intervals and p-values for each cluster, as well as the Concordance Index (CI) associated with the clusters. We also calculated these statistics for standard clinical variables provided by TCGA, such as patient age, gender, race, ethnicity, tumor stage and grade, and found that in several cancers, (e.g. pleural mesothelioma, cutaneous melanomas, head and neck squamous cell carcinomas) the CI of CIMLR clusters exceeded that of all the tested clinical variables.

We then constructed a multivariable Cox regression model for each cancer, including the CIMLR clusters as well as all the clinical variables that were significantly (Wald test $p<0.1$) associated with survival in single-variable Cox regression. In 11 cancers, we found that CIMLR clusters were associated with significant hazard even after adjusting for all tested significant clinical variables (Supplementary Data 7).

We also performed the same analysis in the 5 datasets used for external validation of our results. For all comparisons, the CI was similar in the training and test datasets (Table 2). Moreover, for lower-grade gliomas, cutaneous melanomas, and breast cancer, the stratification of the unseen patients on the basis of CIMLR clusters was significantly associated with survival after adjusting for clinical variables (Supplementary Data 8). These results provide strong evidence that multi-omic subtyping using CIMLR offers significant prognostic value beyond that of commonly used clinical features.

Supplementary Note 2: Thymoma

We used CIMLR to identify subtypes of 116 thymomas. These tumors are normally classified on the basis of histology. However, we found no significant difference in survival between histological types in our data. Instead, CIMLR finds 7 clusters (Supplementary Figure 1A, Supplementary Figure 1B) with a significant difference in overall survival (Supplementary Figure 1C) and pathway activity (Supplementary Figure 1D), each containing a mix of histological types (Supplementary Figure 1E).

Clusters 1 and 2 have high DNA methylation and few mutations or copy number alterations (Supplementary Figure 1G). Cluster 2 is associated with high expression of Myc and E2F targets as well as genes for RNA metabolism, telomere maintenance and DNA synthesis, and low expression of genes for nucleotide excision repair, proteasome and p53 signaling. Clusters 3, 4, and 5 are associated with point mutations in the transcription factor GTF2I (Supplementary Figure 1F), which controls cellular proliferation and has been associated with indolent thymomas[1].

Clusters 6 and 7 have significantly worse overall (log-rank $p=2.2 \times 10^{-3}$) and disease-specific (log-rank $p=2.9 \times 10^{-3}$) survival than the rest of the thymomas. Patients in cluster 6 have a gain on chromosome 1q (65% samples) including cancer-associated genes *SMYD3, PYGO2, ADAM15, UBE2Q1* and *HAX1* (all of these also show increased expression), as well as genes involved in steroid metabolism and phospholipid biosynthesis. 65% also have a loss on 6p including several genes involved in chromatin organization.

Cluster 7 is a mix of histological types, but contains 8 of the 11 type C tumors in the dataset. These tumors share the 1q gain seen in cluster 6; however, only 50% of samples share the 6p loss. In addition, 50% have a loss on 16q, including the tumor suppressor gene *CYLD*, several genes for DNA repair (*POLR2C, TK2*) and chromatin organization (*BRD7, CHMP1A, CTCF*). *POLR2C, TK2, BRD7* and *CTCF* also show reduced expression in the same samples. This cluster is also associated with increased expression of genes for glycolysis and mTORC1 signaling.

Supplementary Figure 1: TCGA - Thymoma

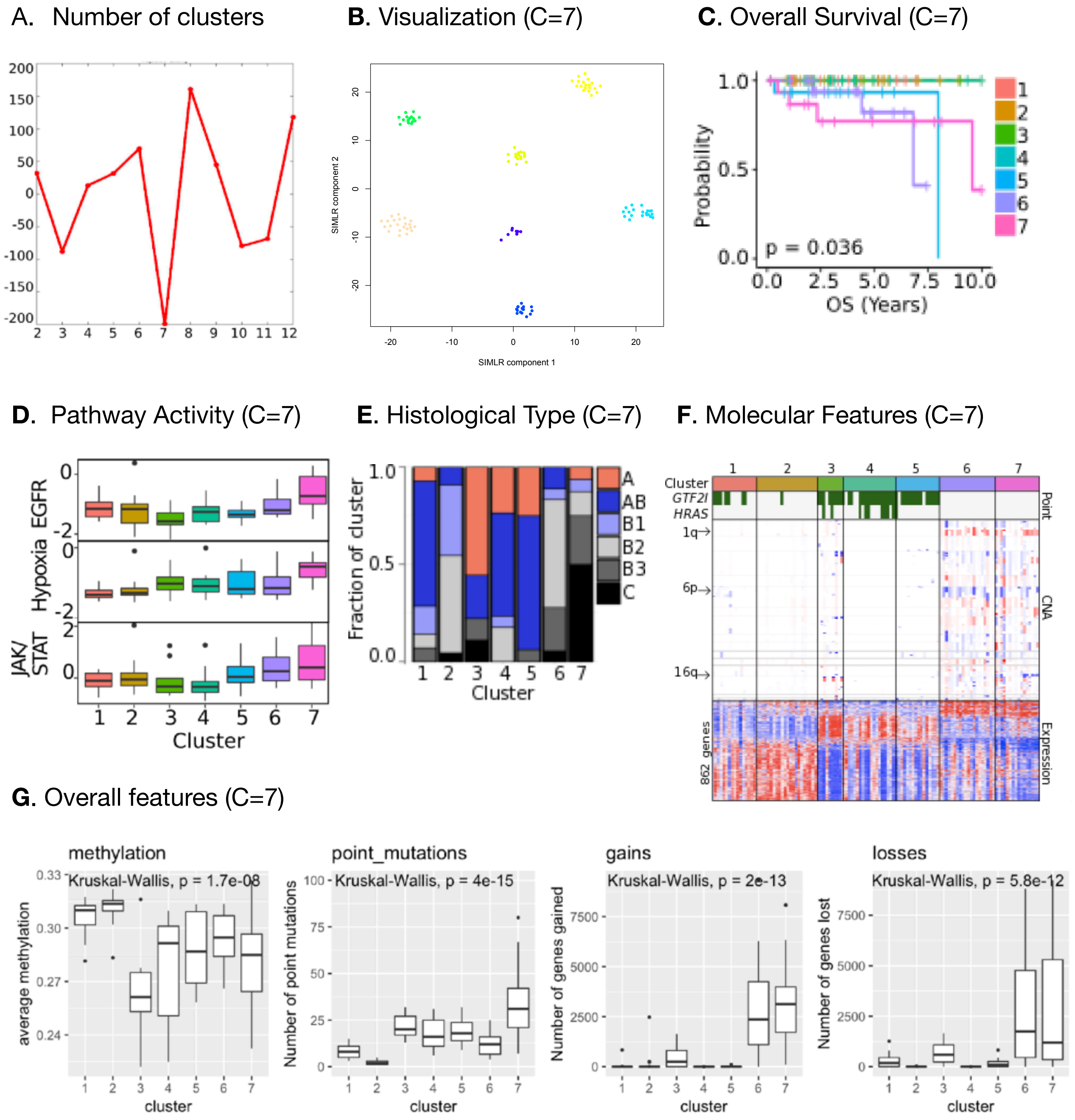

Supplementary Figure 1: TCGA - Thymoma. A. Separation cost[2] (y-axis) for different numbers of clusters (x-axis). A lower y-axis value indicates a number of clusters that fits the data better than the previous number. **B**. 2-D visualization of clusters. **C**. Kaplan-Meier curves showing overall survival for the clusters. Survival data was censored as described in Methods. P-value is from log-rank test. **D**. Bar plots showing distribution of histological types within clusters. **E**. Boxplots showing activity of EGFR, Hypoxia and JAK/STAT pathways in the clusters. **F**. Selected molecular features that differentiate the clusters. Copy number alterations (CNA) and RNA expression are shown along a blue (low) to red (high) spectrum. **G**. Boxplots showing average methylation beta values, number of genes with somatic coding point mutations, number of genes with copy number gain and number of genes with copy number loss, for each cluster.

Supplementary Figure 2: TCGA - Acute Myeloid Leukemia

A. Number of clusters

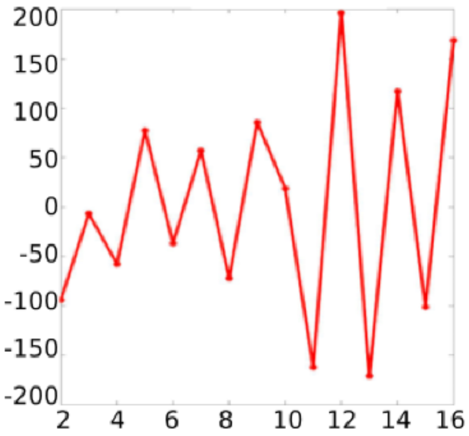

B. Visualization (C=13)

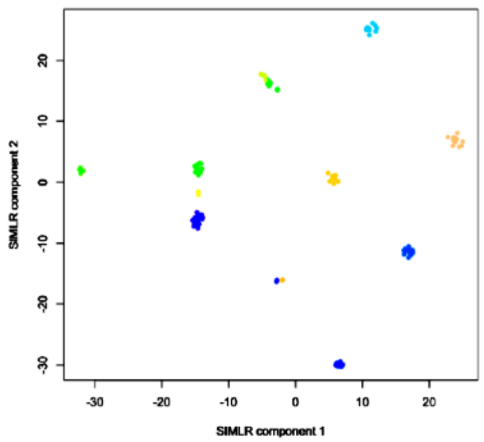

C. Overall Survival (C=13)

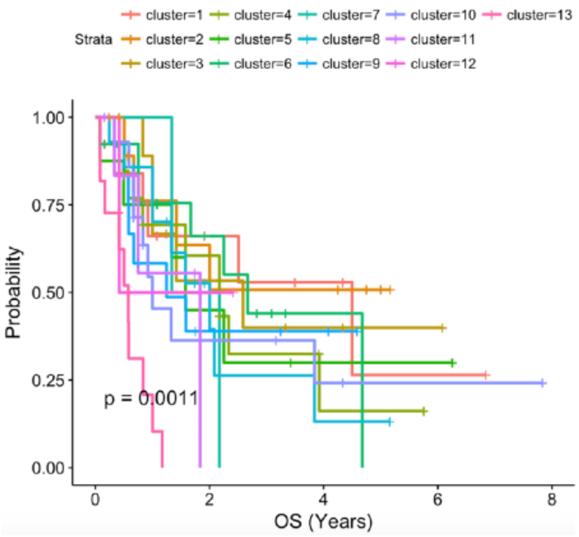

D. Features (C=13)

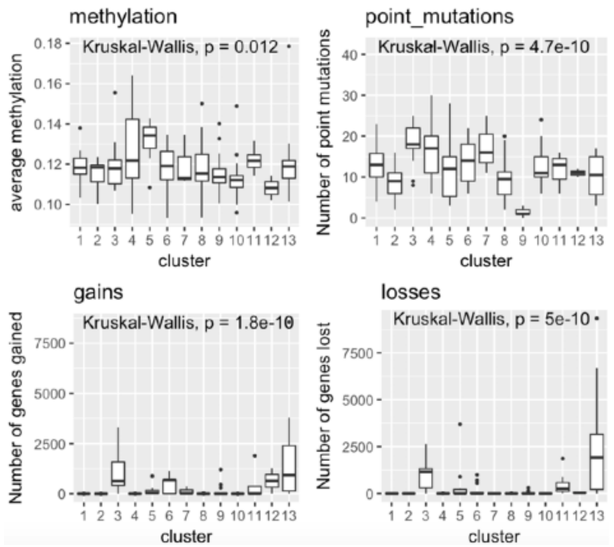

Supplementary Figure 2: TCGA - Acute Myeloid Leukemia. A. Separation cost[2] (y-axis) for different numbers of clusters (x-axis). A lower y-axis value indicates a number of clusters that fits the data better than the previous number. **B.** 2-D visualization of clusters. **C.** Kaplan-Meier curves showing overall survival for the clusters. Survival data was censored as described in Methods. P-value is from log-rank test. **D.** Boxplots showing average methylation beta values, number of genes with somatic coding point mutations, number of genes with copy number gain and number of genes with copy number loss, for each cluster.

Supplementary Figure 3: TCGA - Adrenocortical carcinoma

A. Number of clusters

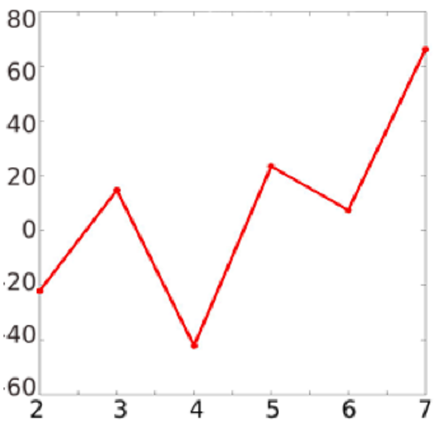

B. Visualization (C=6)

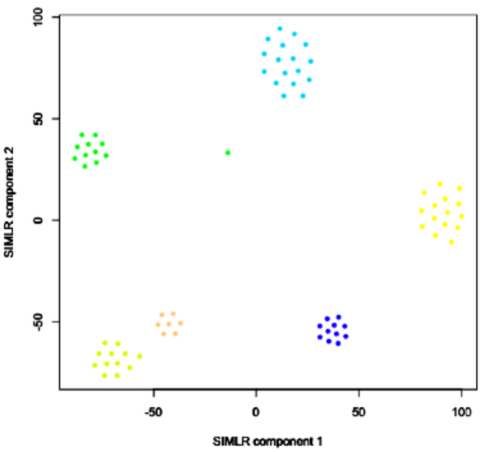

C. Overall Survival (C=6)

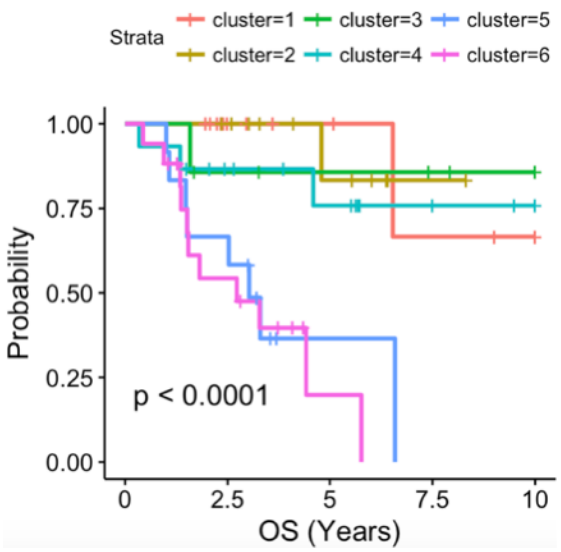

D. Features (C=6)

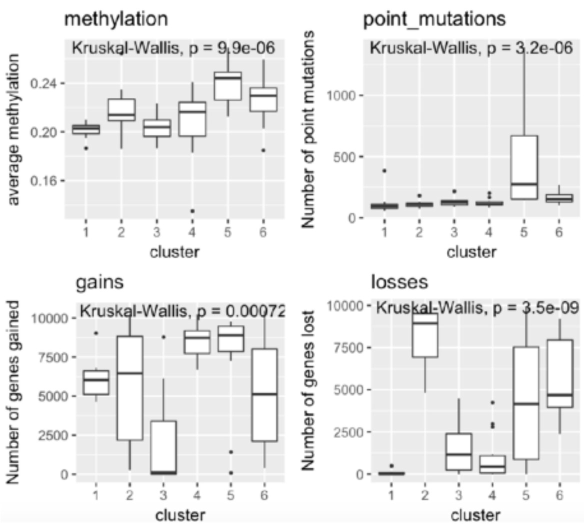

Supplementary Figure 3: TCGA - Adrenocortical carcinoma. A. Separation cost (y-axis) for different numbers of clusters (x-axis). A lower y-axis value indicates a number of clusters that fits the data better than the previous number. **B**. 2-D visualization of clusters. **C**. Kaplan-Meier curves showing overall survival for the clusters. Survival data was censored as described in Methods. P-value is from log-rank test. **D**. Boxplots showing average methylation beta values, number of genes with somatic coding point mutations, number of genes with copy number gain and number of genes with copy number loss, for each cluster.

Supplementary Figure 4: TCGA - Breast cancer

A. Number of clusters

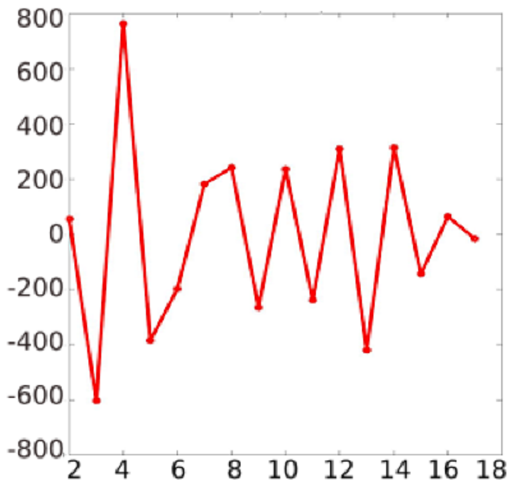

B. Visualization (C=13)

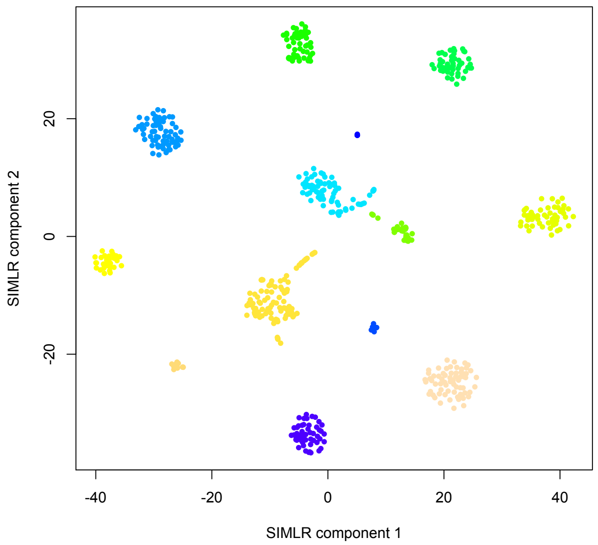

C. Features (C=13)

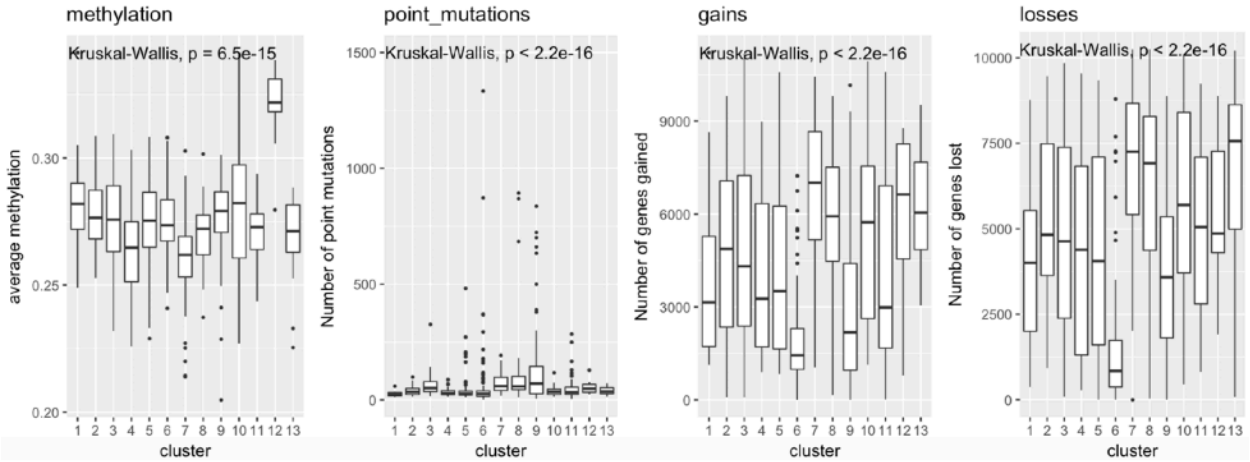

Supplementary Figure 4: TCGA - Breast Cancer. A. Separation cost (y-axis) for different numbers of clusters (x-axis). A lower y-axis value indicates a number of clusters that fits the data better than the previous number. **B**. 2-D visualization of clusters. **C**. Boxplots showing average methylation beta values, number of genes with somatic coding point mutations, number of genes with copy number gain and number of genes with copy number loss, for each cluster.

Supplementary Figure 5: TCGA - Cervical cancer

A. Number of clusters

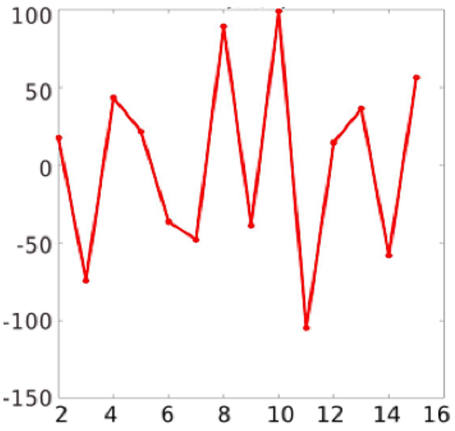

B. Visualization (C=11)

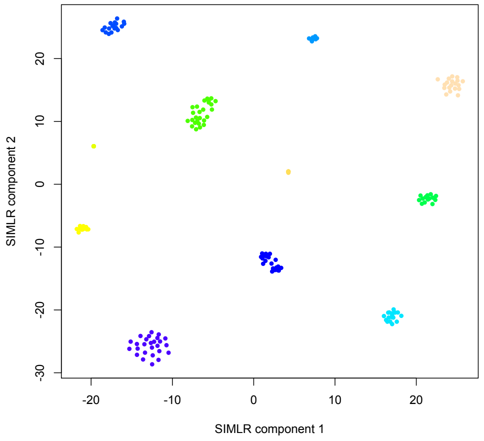

C. Overall Survival (C=11)

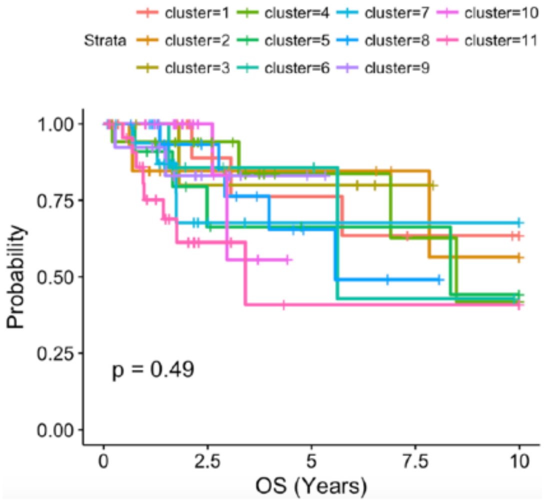

D. Features (C=11)

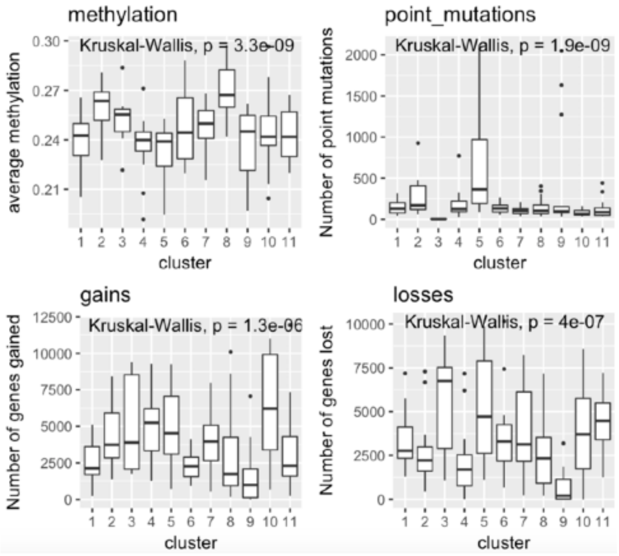

Supplementary Figure 5: TCGA - Cervical cancer. A. Separation cost (y-axis) for different numbers of clusters (x-axis). A lower y-axis value indicates a number of clusters that fits the data better than the previous number. **B**. 2-D visualization of clusters. **C**. Kaplan-Meier curves showing overall survival for the clusters. Survival data was censored as described in Methods. P-value is from log-rank test. **D**. Boxplots showing average methylation beta values, number of genes with somatic coding point mutations, number of genes with copy number gain and number of genes with copy number loss, for each cluster.

Supplementary Figure 6: TCGA - Cholangiocarcinoma

A. Number of clusters

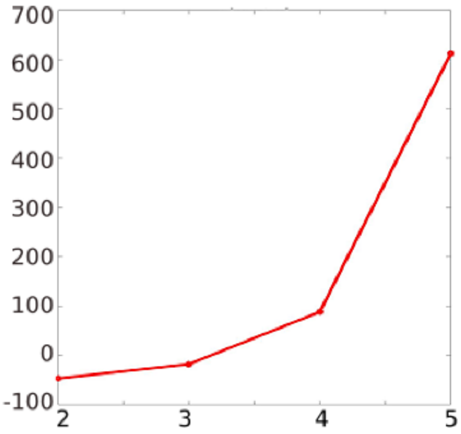

B. Visualization (C=3)

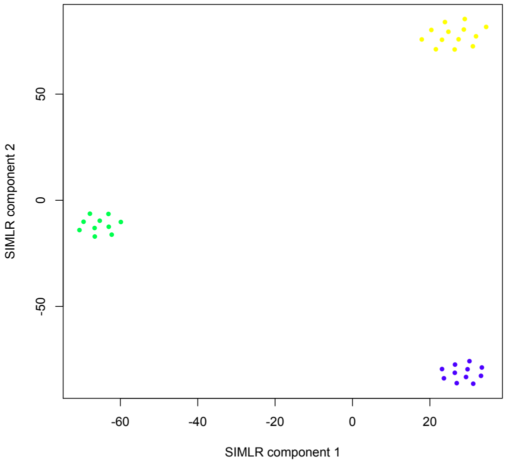

C. Overall survival (C=3)

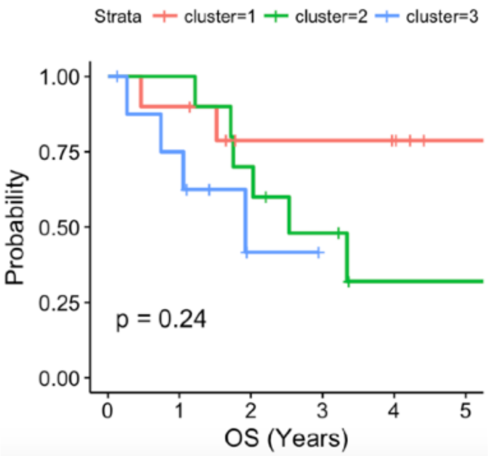

D. Features (C=3)

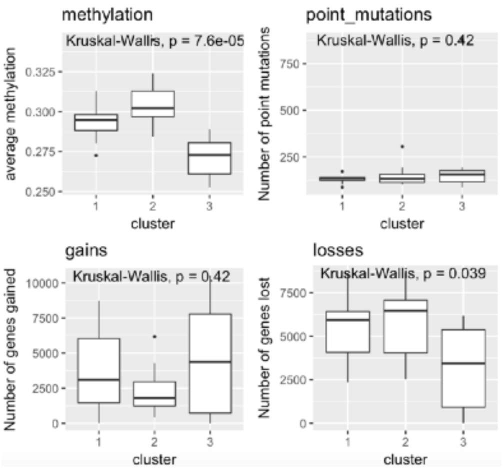

Supplementary Figure 6: TCGA - Cholangiocarcinoma. A. Separation cost (y-axis) for different numbers of clusters (x-axis). A lower y-axis value indicates a number of clusters that fits the data better than the previous number. **B**. 2-D visualization of clusters. **C**. Kaplan-Meier curves showing overall survival for the clusters. Survival data was censored as described in Methods. P-value is from log-rank test. **D**. Boxplots showing average methylation beta values, number of genes with somatic coding point mutations, number of genes with copy number gain and number of genes with copy number loss, for each cluster.

Supplementary Figure 7: TCGA - Chromophobe Renal Cell Carcinoma

A. Number of clusters

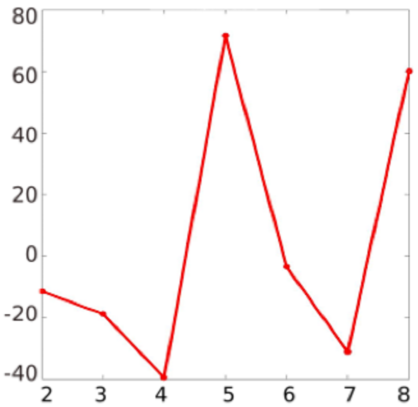

B. Visualization (C=7)

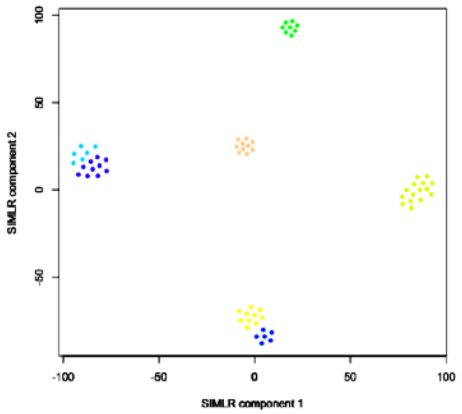

C. Overall survival (C=7)

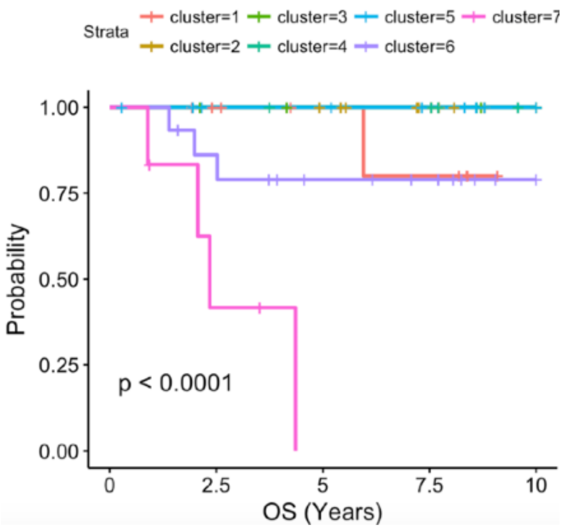

D. Features (C=7)

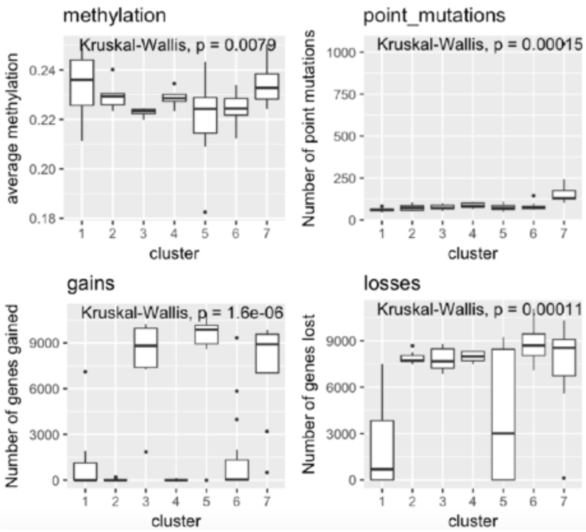

Supplementary Figure 7: TCGA - Chromophobe Renal Cell Carcinoma. A. Separation cost (y-axis) for different numbers of clusters (x-axis). A lower y-axis value indicates a number of clusters that fits the data better than the previous number. **B**. 2-D visualization of clusters. **C.** Kaplan-Meier curves showing overall survival for the clusters. Survival data was censored as described in Methods. P-value is from log-rank test. **D**. Boxplots showing average methylation beta values, number of genes with somatic coding point mutations, number of genes with copy number gain and number of genes with copy number loss, for each cluster.

Supplementary Figure 8: TCGA - Clear Cell Renal Cell Carcinoma

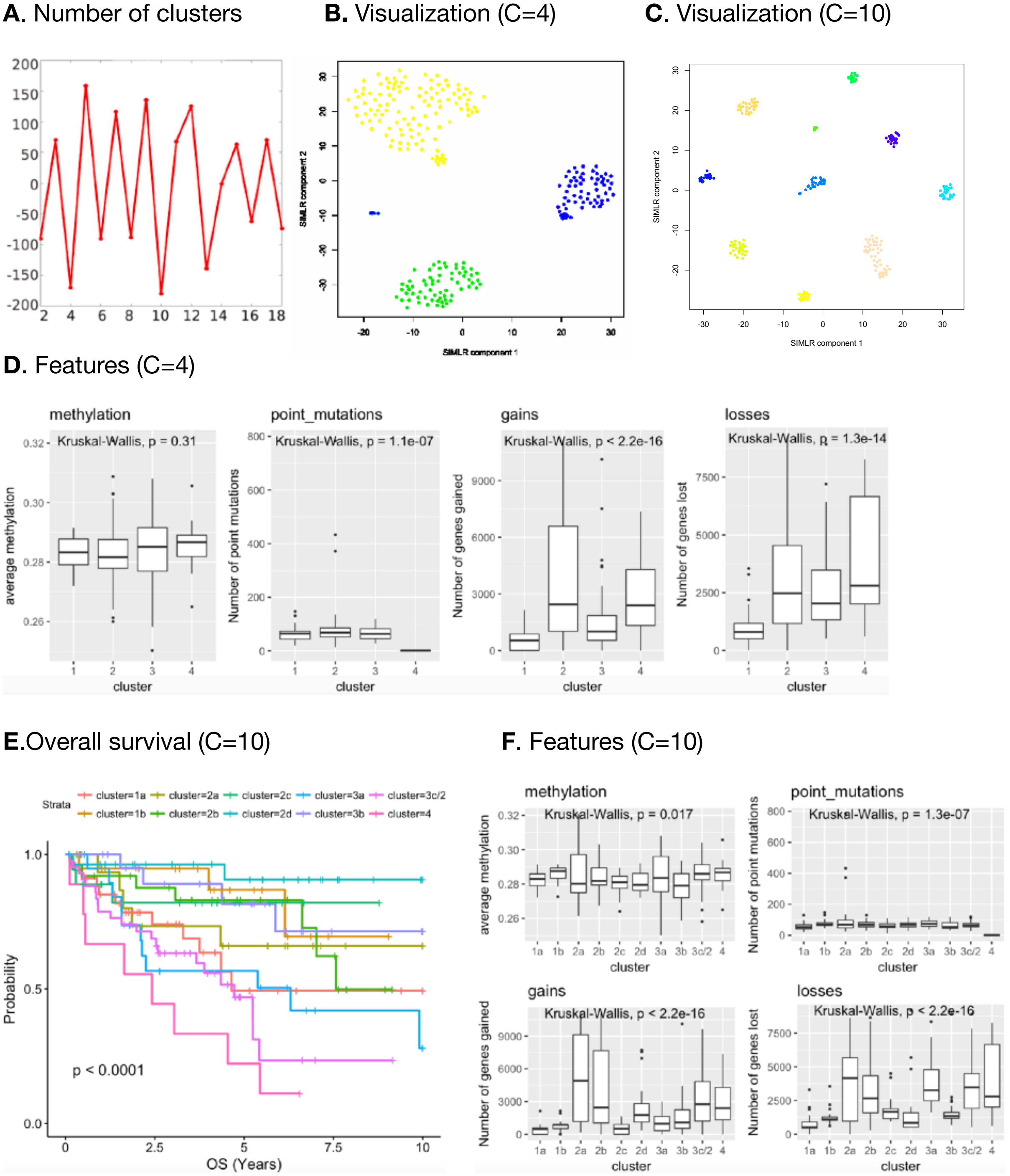

Supplementary Figure 8: TCGA - Clear Cell Renal Cell Carcinoma. A. Separation cost (y-axis) for different numbers of clusters (x-axis). A lower y-axis value indicates a number of clusters that fits the data better than the previous number. **B**. 2-D visualization of 4 clusters. **C**. 2-D visualization of 10 clusters. **D**. Boxplots showing average methylation beta values, number of genes with somatic coding point mutations, number of genes with copy number gain and number of genes with copy number loss, for each of 4 clusters. **E**. Kaplan-Meier curves showing overall survival for the 10 clusters. Survival data was censored as described in Methods. P-value is from log-rank test. **F**. Boxplots showing average methylation beta values, number of genes with somatic coding point mutations, number of genes with copy number gain and number of genes with copy number loss, for each of the 10 clusters.

Supplementary Figure 9: TCGA - Colon/Rectal cancer

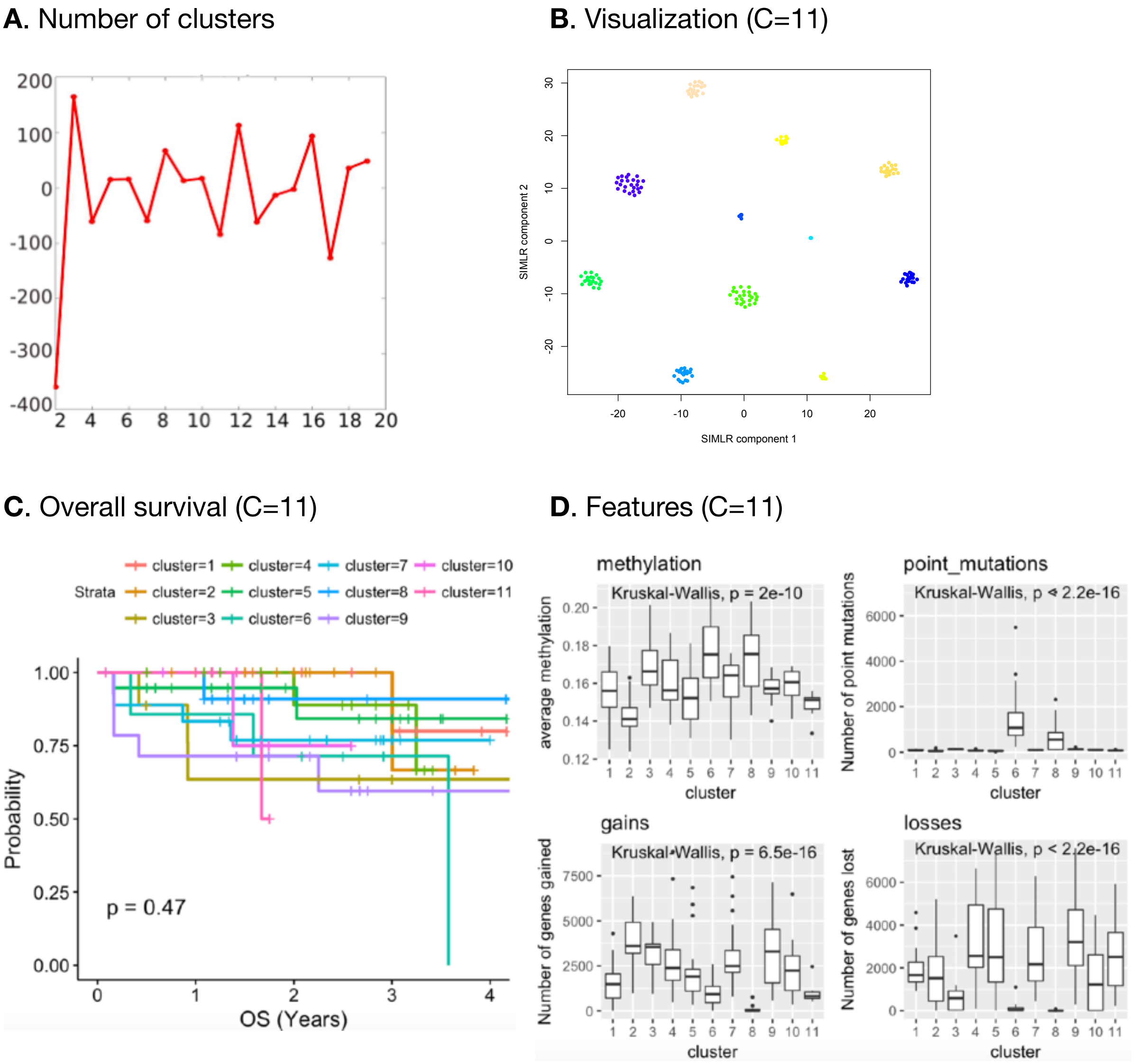

Supplementary Figure 9: TCGA - Colon/Rectal cancer. A. Separation cost (y-axis) for different numbers of clusters (x-axis). A lower y-axis value indicates a number of clusters that fits the data better than the previous number. **B**. 2-D visualization of clusters. **C**. Kaplan-Meier curves showing overall survival for the clusters. Survival data was censored as described in Methods. P-value is from log-rank test. **D**. Boxplots showing average methylation beta values, number of genes with somatic coding point mutations, number of genes with copy number gain and number of genes with copy number loss, for each cluster.

Supplementary Figure 10: TCGA - Cutaneous Melanoma

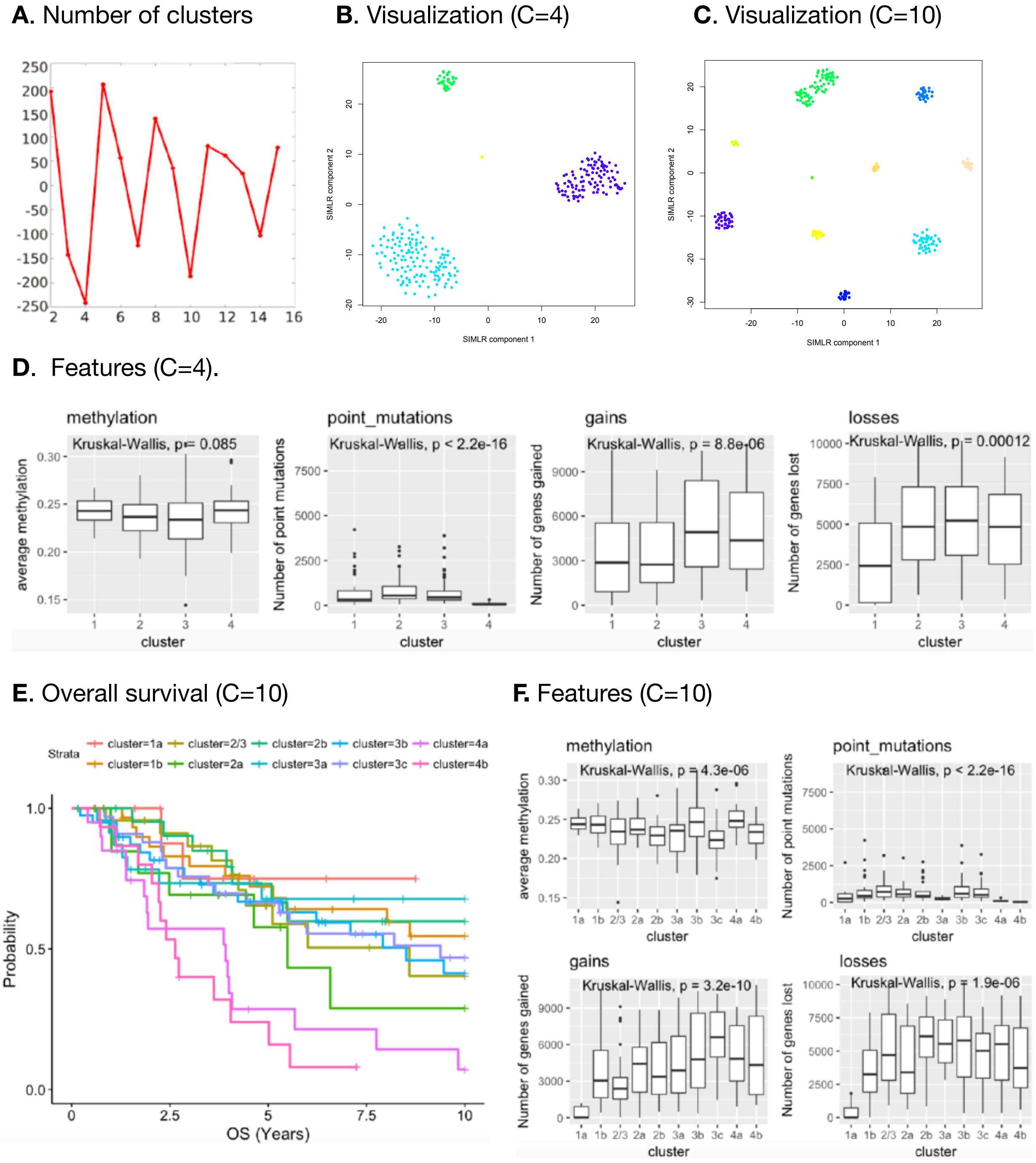

Supplementary Figure 10: TCGA - Cutaneous Melanoma. A. Separation cost (y-axis) for different numbers of clusters (x-axis). A lower y-axis value indicates a number of clusters that fits the data better than the previous number. **B**. 2-D visualization of 4 clusters. **C**. 2-D visualization of 10 clusters. **D**. Boxplots showing average methylation beta values, number of genes with somatic coding point mutations, number of genes with copy number gain and number of genes with copy number loss, for each of 4 clusters. **E**. Kaplan-Meier curves showing overall survival for the 10 clusters. Survival data was censored as described in Methods. P-value is from log-rank test. **F**. Boxplots showing average methylation beta values, number of genes with somatic coding point mutations, number of genes with copy number gain and number of genes with copy number loss, for each of the 10 clusters.

Supplementary Figure 11: TCGA - Endometrial carcinoma

A. Number of clusters

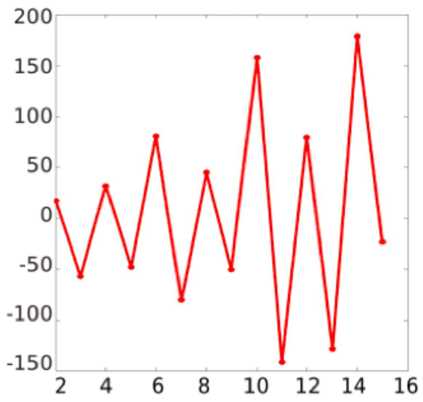

B. Visualization (C=11)

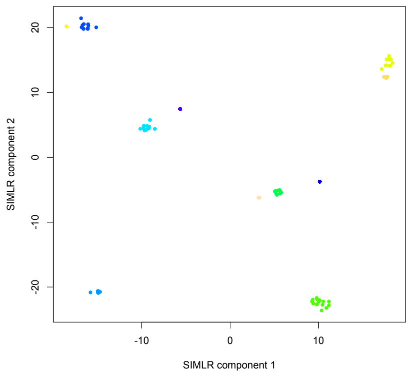

C. Overall survival (C=11)

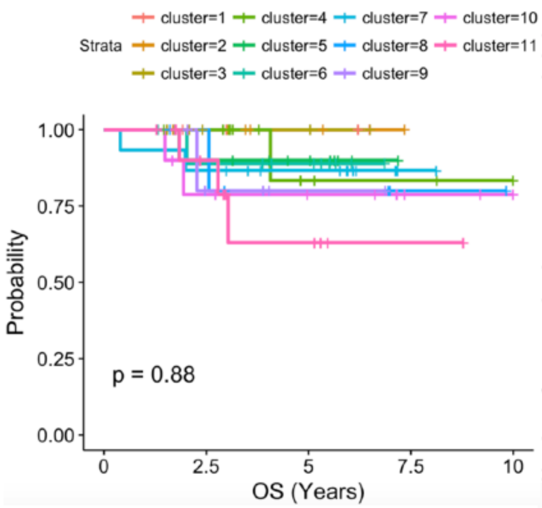

D. Features (C=11)

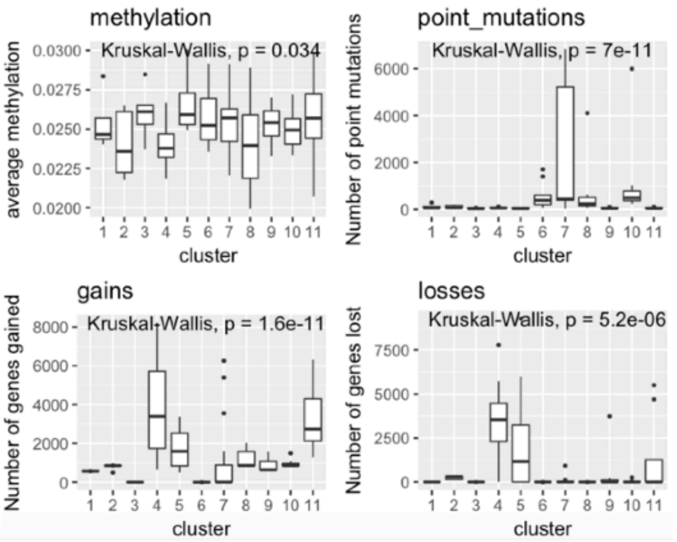

Supplementary Figure 11: TCGA - Endometrial carcinoma. A. Separation cost (y-axis) for different numbers of clusters (x-axis). A lower y-axis value indicates a number of clusters that fits the data better than the previous number. **B**. 2-D visualization of clusters. **C**. Kaplan-Meier curves showing overall survival for the clusters. Survival data was censored as described in Methods. P-value is from log-rank test. **D**. Boxplots showing average methylation beta values, number of genes with somatic coding point mutations, number of genes with copy number gain and number of genes with copy number loss, for each cluster.

Supplementary Figure 12: TCGA - Gastric Adenocarcinoma

A. Number of clusters

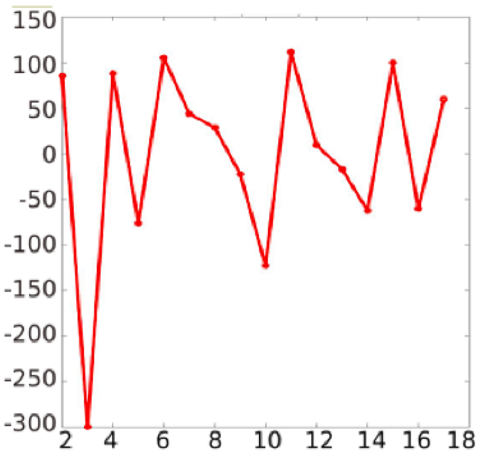

B. Visualization

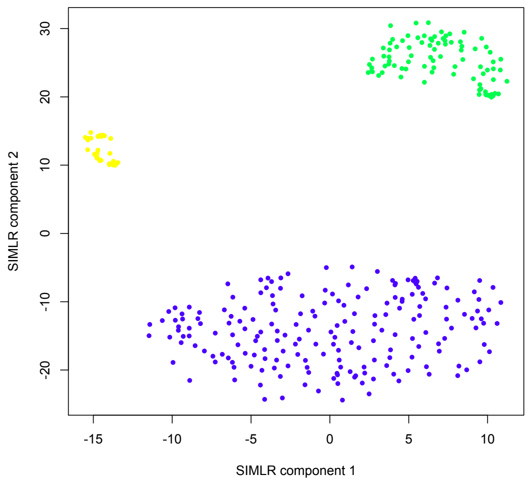

C. Overall survival

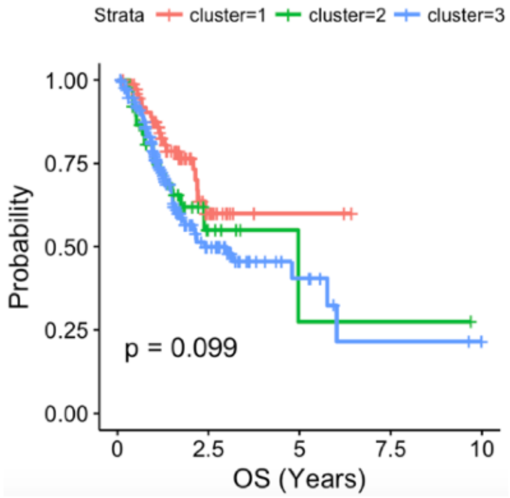

D. Features

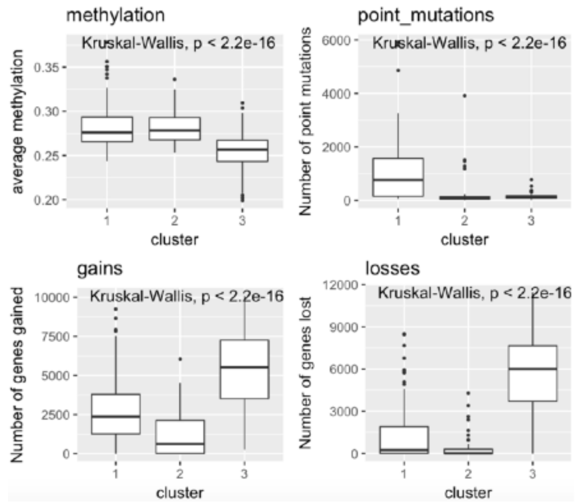

Supplementary Figure 12: TCGA - Gastric Adenocarcinoma. **A**. Separation cost (y-axis) for different numbers of clusters (x-axis). A lower y-axis value indicates a number of clusters that fits the data better than the previous number. **B**. 2-D visualization of clusters. **C**. Kaplan-Meier curves showing overall survival for the clusters. Survival data was censored as described in Methods. P-value is from log-rank test. **D**. Boxplots showing average methylation beta values, number of genes with somatic coding point mutations, number of genes with copy number gain and number of genes with copy number loss, for each cluster.

Supplementary Figure 13: TCGA - Glioblastoma

A. Number of clusters

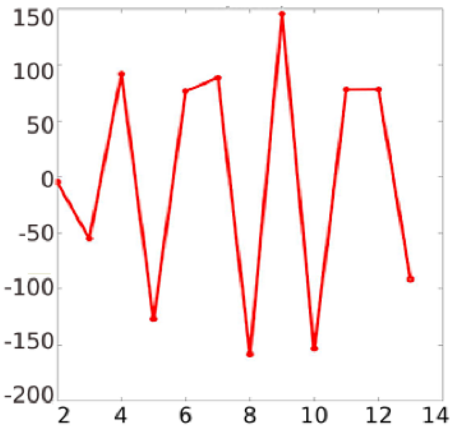

B. Visualization (C=8)

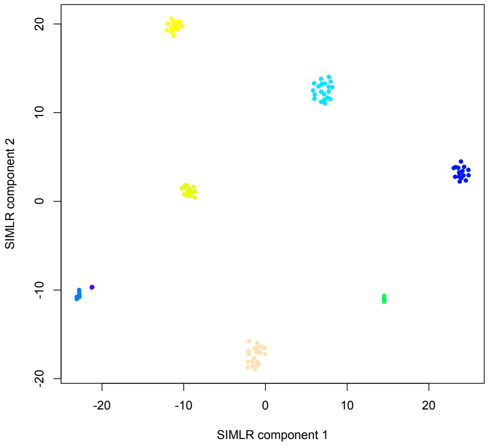

C. Overall survival (C=8)

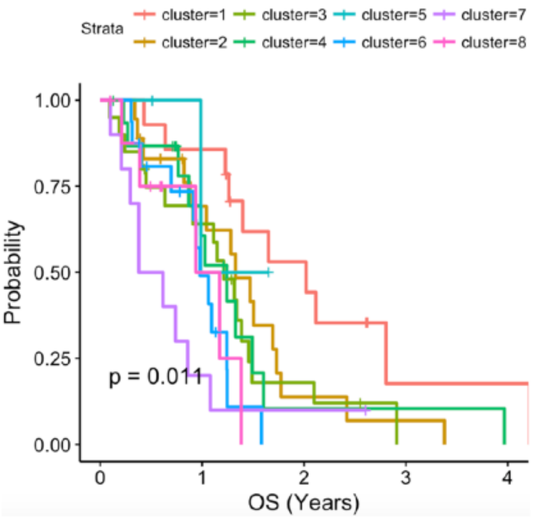

D. Features (C=8)

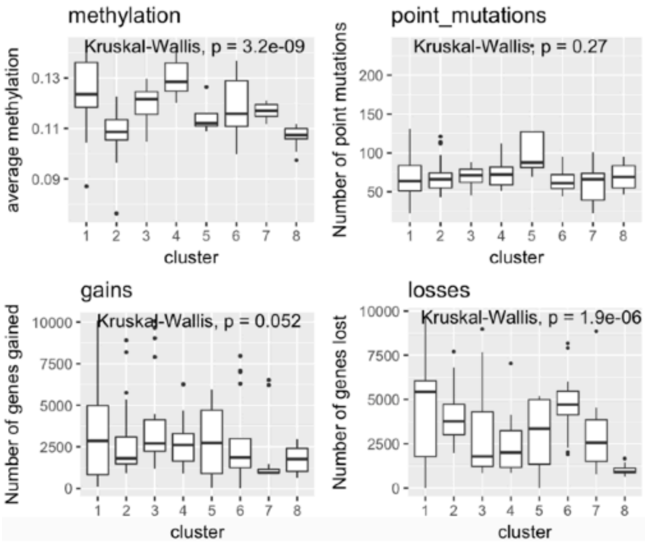

Supplementary Figure 13: TCGA - Glioblastoma. A. Separation cost (y-axis) for different numbers of clusters (x-axis). A lower y-axis value indicates a number of clusters that fits the data better than the previous number. **B**. 2-D visualization of clusters. **C**. Kaplan-Meier curves showing overall survival for the clusters. Survival data was censored as described in Methods. P-value is from log-rank test. **D**. Boxplots showing average methylation beta values, number of genes with somatic coding point mutations, number of genes with copy number gain and number of genes with copy number loss, for each cluster.

Supplementary Figure 14: TCGA - Head and Neck Squamous Cell Carcinoma

A. Number of clusters

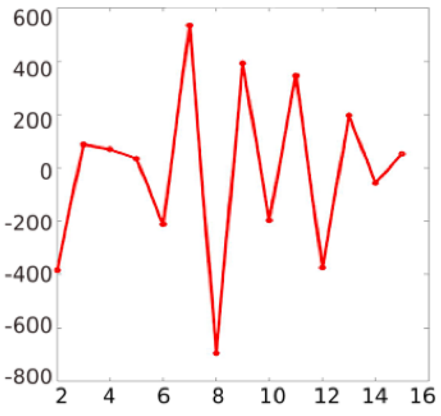

B. Visualization (C=8)

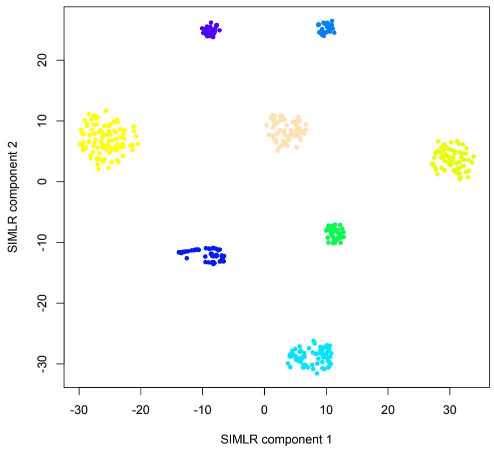

C. Features (C=8)

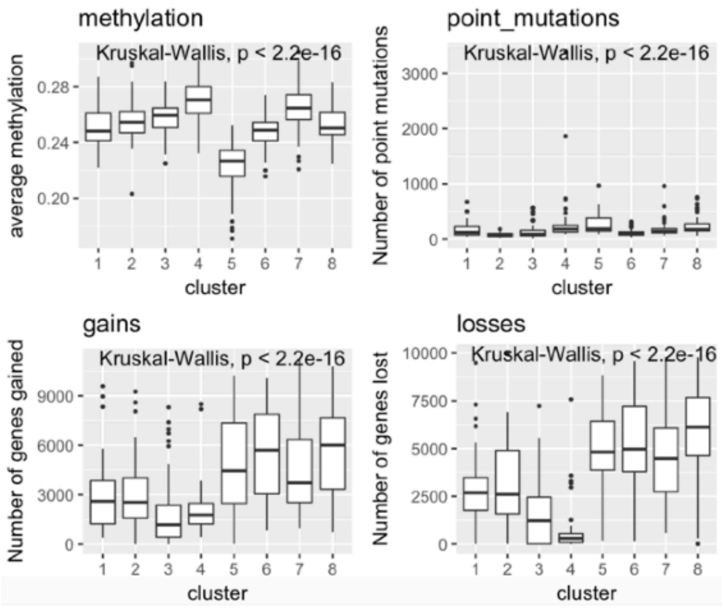

Supplementary Figure 14: TCGA - Head and Neck Squamous Cell Carcinoma. A. Separation cost (y-axis) for different numbers of clusters (x-axis). A lower y-axis value indicates a number of clusters that fits the data better than the previous number. **B**. 2-D visualization of clusters. **C**. Boxplots showing average methylation beta values, number of genes with somatic coding point mutations, number of genes with copy number gain and number of genes with copy number loss, for each cluster.

Supplementary Figure 15: TCGA - Liver Hepatocellular Carcinoma

A. Number of clusters

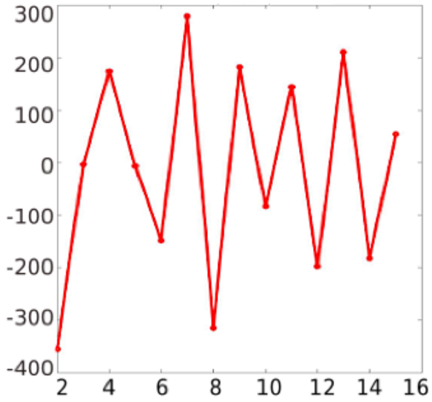

B. Visualization (C=8)

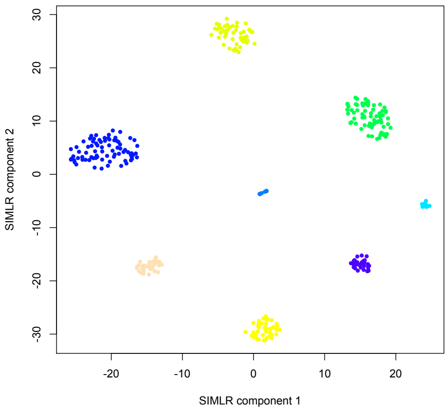

C. Features (C=8)

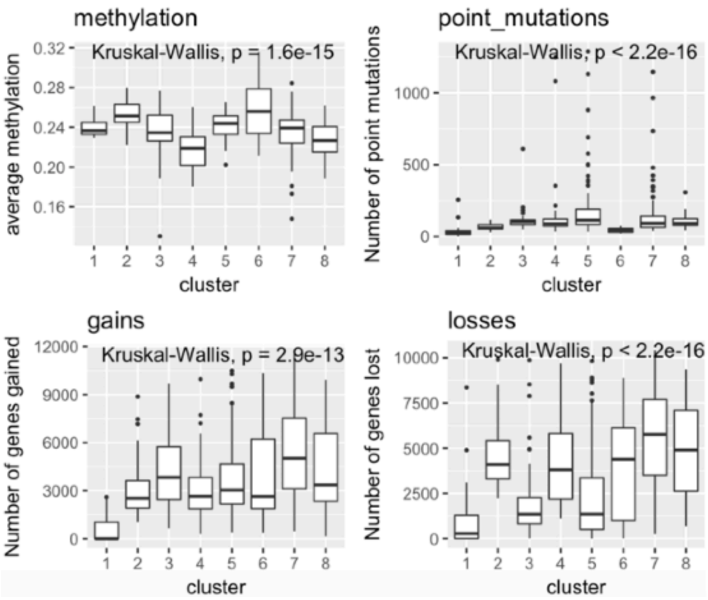

Supplementary Figure 15: TCGA - Liver Hepatocellular Carcinoma. A. Separation cost (y-axis) for different numbers of clusters (x-axis). A lower y-axis value indicates a number of clusters that fits the data better than the previous number. **B**. 2-D visualization of clusters. **C**. Boxplots showing average methylation beta values, number of genes with somatic coding point mutations, number of genes with copy number gain and number of genes with copy number loss, for each cluster.

Supplementary Figure 16: TCGA - Lower grade glioma

A. Features (C=3)

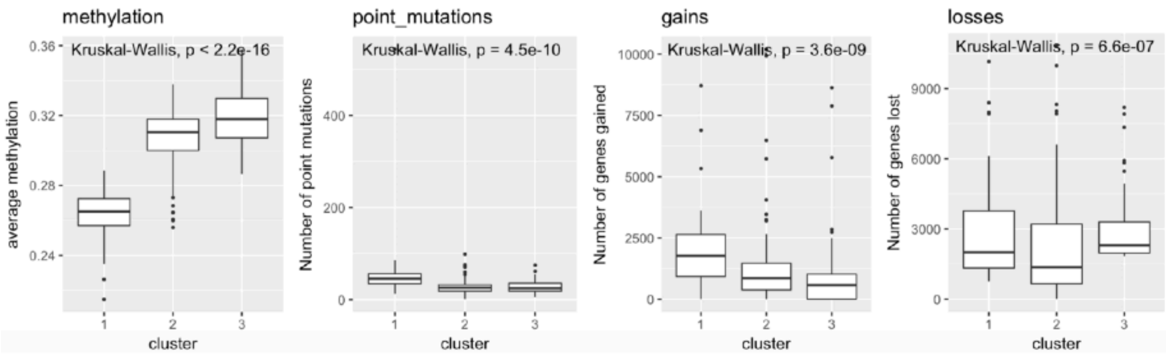

B. Visualization (C=7)

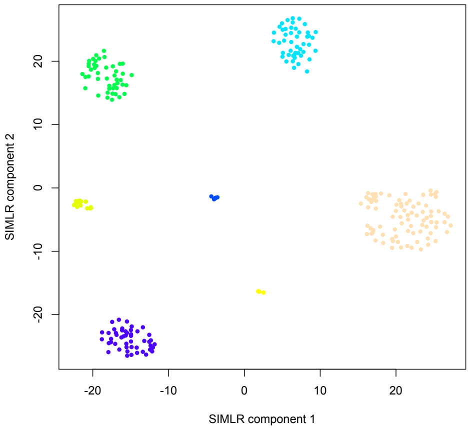

C. Overall survival (C=7)

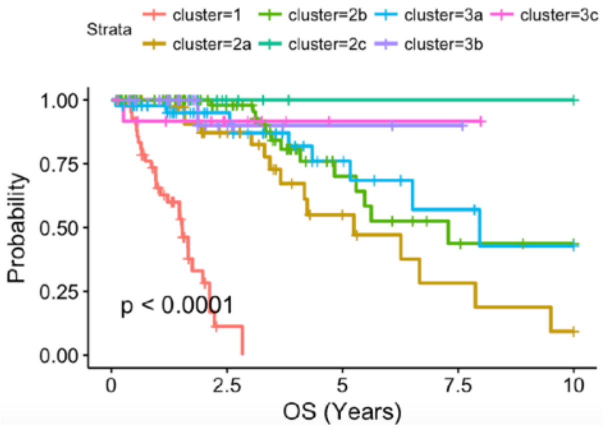

D. Features (C=7)

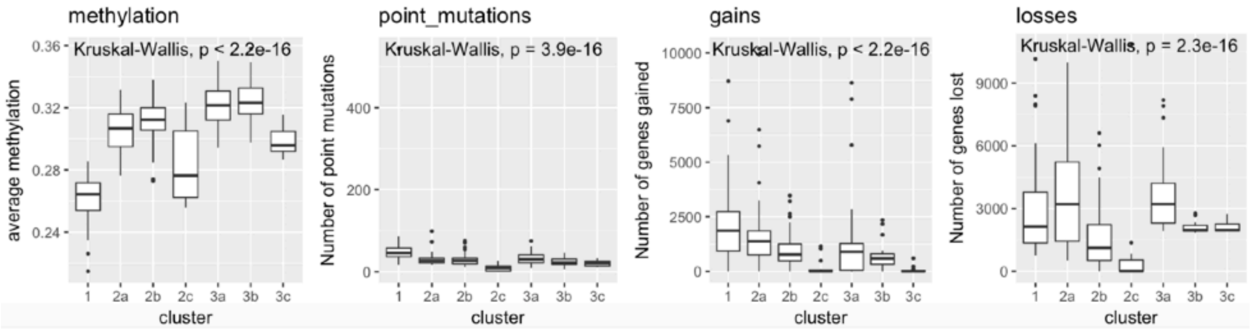

Supplementary Figure 16: TCGA - Lower grade glioma. A. Boxplots showing average methylation beta values, number of genes with somatic coding point mutations, number of genes with copy number gain and number of genes with copy number loss, for each of 3 clusters. **B**. 2-D visualization of 7 subclusters. **C**. Kaplan-Meier curves showing overall survival for the 7 subclusters. Survival data was censored as described in Methods. P-value is from log-rank test. **D**. Boxplots showing average methylation beta values, number of genes with somatic coding point mutations, number of genes with copy number gain and number of genes with copy number loss, for each of the 7 subclusters.

Supplementary Figure 17: TCGA - Lung Adenocarcinoma

A. Number of clusters.

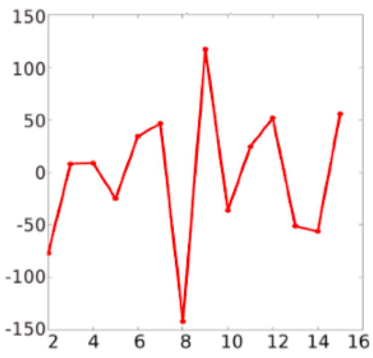

B. Visualization (C=8)

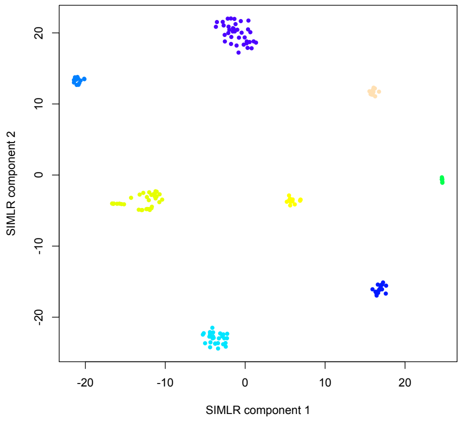

C. Features (C=8)

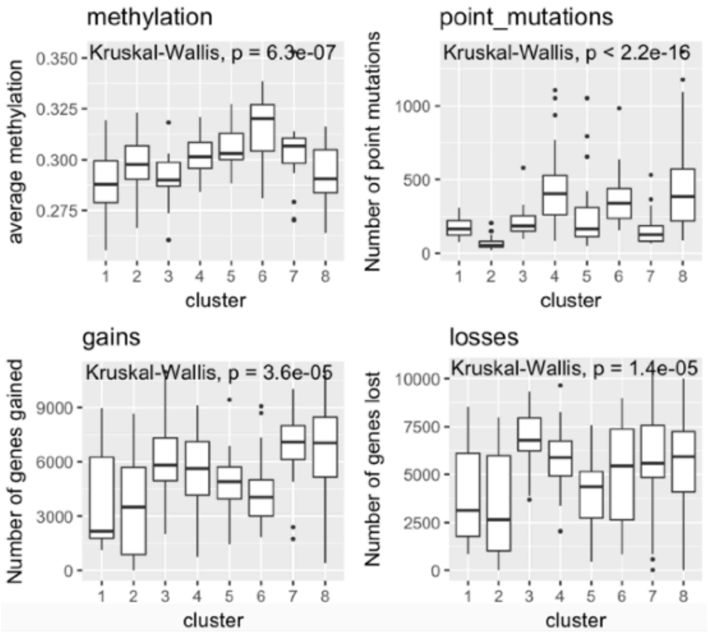

Supplementary Figure 17: TCGA - Lung Adenocarcinoma. A. Separation cost (y-axis) for different numbers of clusters (x-axis). A lower y-axis value indicates a number of clusters that fits the data better than the previous number. **B**. 2-D visualization of clusters. **C**. Boxplots showing average methylation beta values, number of genes with somatic coding point mutations, number of genes with copy number gain and number of genes with copy number loss, for each cluster.

Supplementary Figure 18: TCGA - Lymphoid Neoplasm Diffuse Large B cell Lymphoma

A. Number of clusters

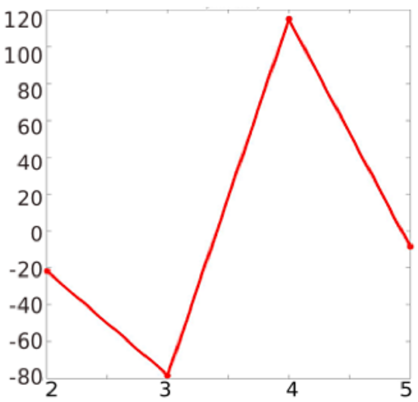

B. Visualization (C=3)

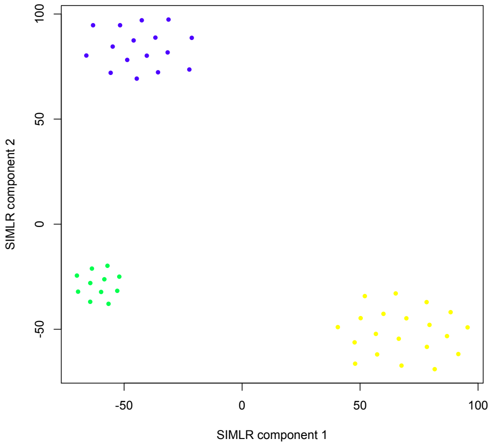

C. Overall survival (C=3)

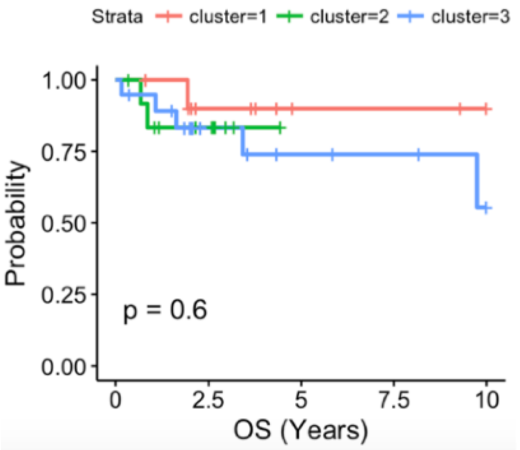

D. Features (C=3)

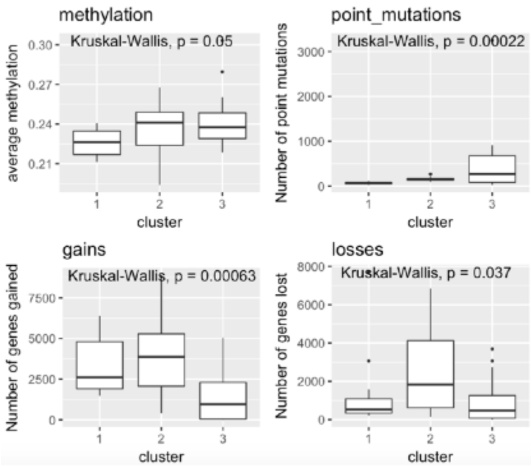

Supplementary Figure 18: TCGA - Lymphoid Neoplasm Diffuse Large B cell Lymphoma. **A.** Separation cost (y-axis) for different numbers of clusters (x-axis). A lower y-axis value indicates a number of clusters that fits the data better than the previous number. **B.** 2-D visualization of clusters. **C.** Kaplan-Meier curves showing overall survival for the clusters. Survival data was censored as described in Methods. P-value is from log-rank test. **D.** Boxplots showing average methylation beta values, number of genes with somatic coding point mutations, number of genes with copy number gain and number of genes with copy number loss, for each cluster.

Supplementary Figure 19: TCGA - Oesophageal Carcinoma

A. Number of clusters

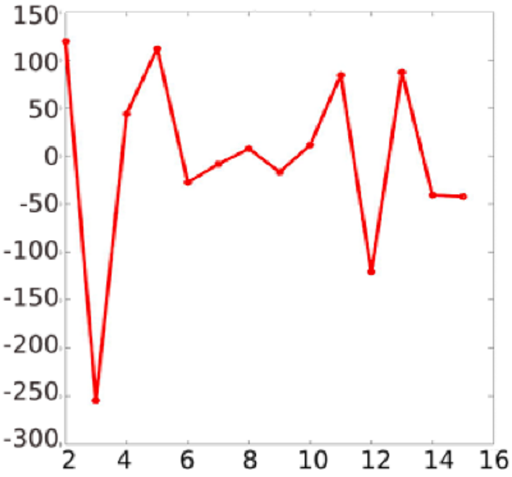

B. Visualization (C=3)

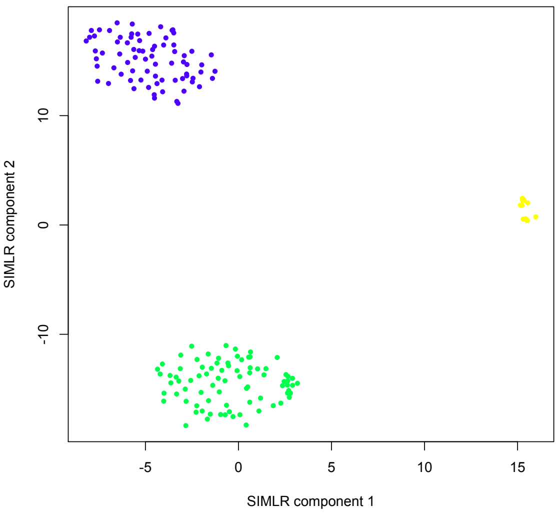

C. Overall survival (C=3)

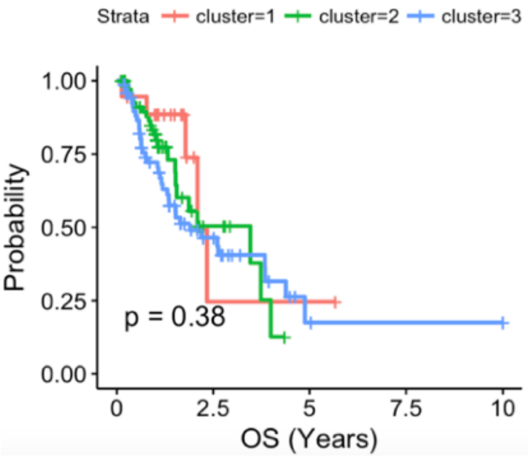

D. Features (C=3)

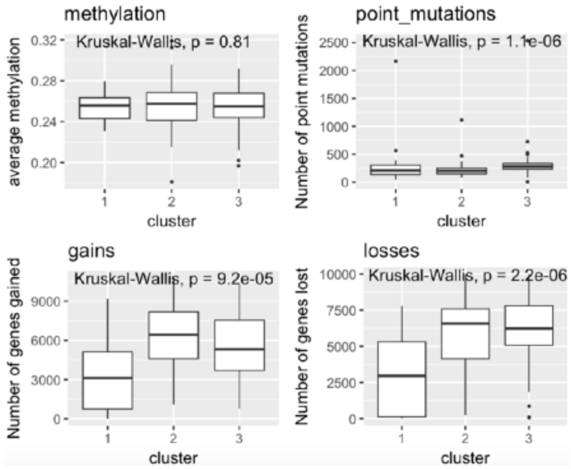

Supplementary Figure 19: TCGA - Oesophageal Carcinoma. A. Separation cost (y-axis) for different numbers of clusters (x-axis). A lower y-axis value indicates a number of clusters that fits the data better than the previous number. **B**. 2-D visualization of clusters. **C**. Kaplan-Meier curves showing overall survival for the clusters. Survival data was censored as described in Methods. P-value is from log-rank test. **D**. Boxplots showing average methylation beta values, number of genes with somatic coding point mutations, number of genes with copy number gain and number of genes with copy number loss, for each cluster.

Supplementary Figure 20: TCGA - Ovarian Carcinoma

A. Number of clusters

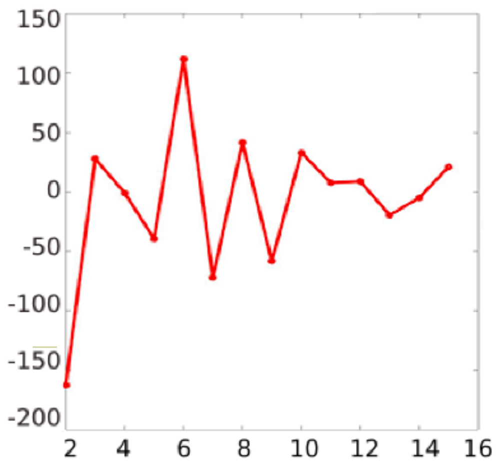

B. Visualization (C=2)

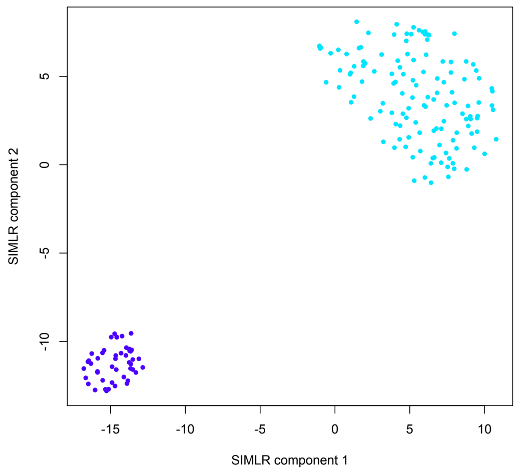

C. Overall survival (C=2)

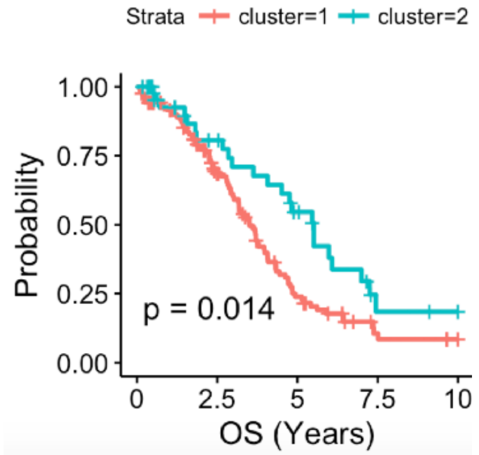

D. Features (C=2)

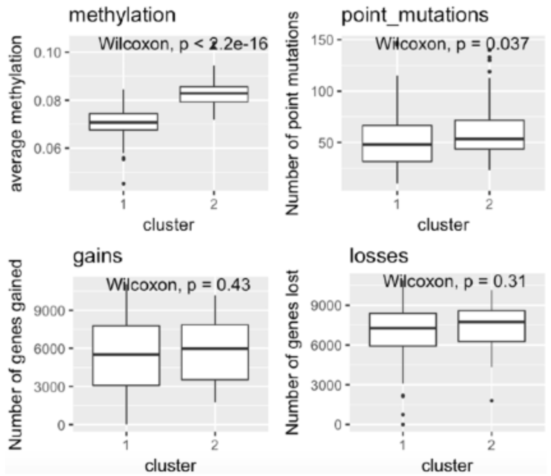

Supplementary Figure 20: TCGA - Ovarian Carcinoma. A. Separation cost (y-axis) for different numbers of clusters (x-axis). A lower y-axis value indicates a number of clusters that fits the data better than the previous number. **B**. 2-D visualization of clusters. **C**. Kaplan-Meier curves showing overall survival for the clusters. Survival data was censored as described in Methods. P-value is from log-rank test. **D**. Boxplots showing average methylation beta values, number of genes with somatic coding point mutations, number of genes with copy number gain and number of genes with copy number loss, for each cluster.

Supplementary Figure 21: TCGA - Pancreatic Adenocarcinoma

A. Number of clusters

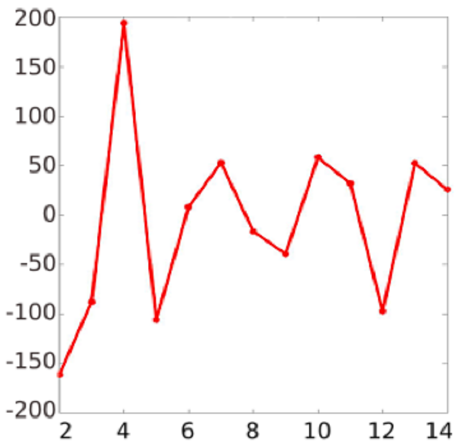

B. Visualization

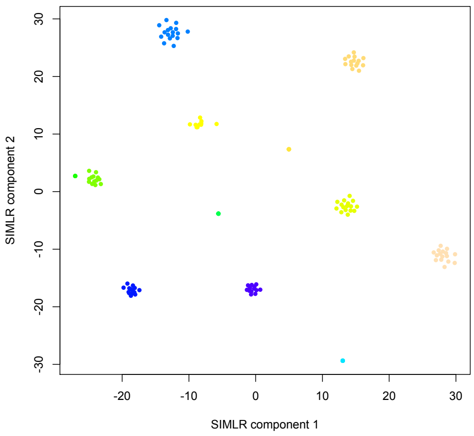

C. Overall survival

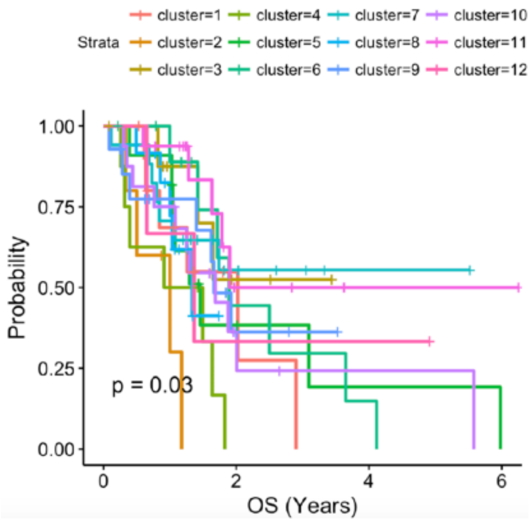

D. Features

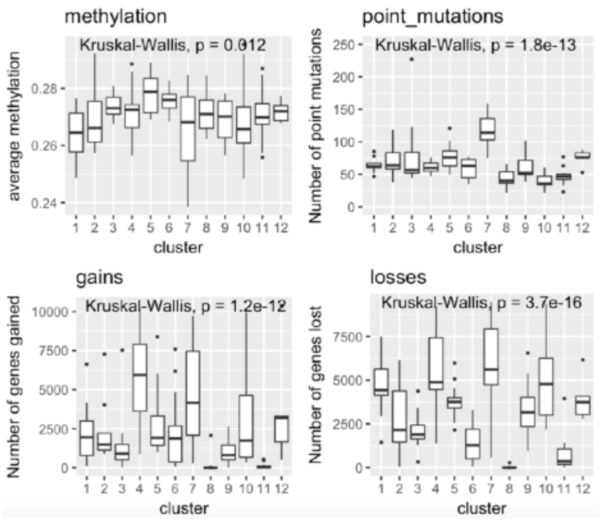

Supplementary Figure 21: TCGA - Pancreatic Adenocarcinoma. A. Separation cost (y-axis) for different numbers of clusters (x-axis). A lower y-axis value indicates a number of clusters that fits the data better than the previous number. **B**. 2-D visualization of clusters. **C**. Kaplan-Meier curves showing overall survival for the clusters. Survival data was censored as described in Methods. P-value is from log-rank test. **D**. Boxplots showing average methylation beta values, number of genes with somatic coding point mutations, number of genes with copy number gain and number of genes with copy number loss, for each cluster.

Supplementary Figure 22: TCGA - Papillary Renal Cell Carcinoma

A. Number of clusters **B.** Visualization (C=8)

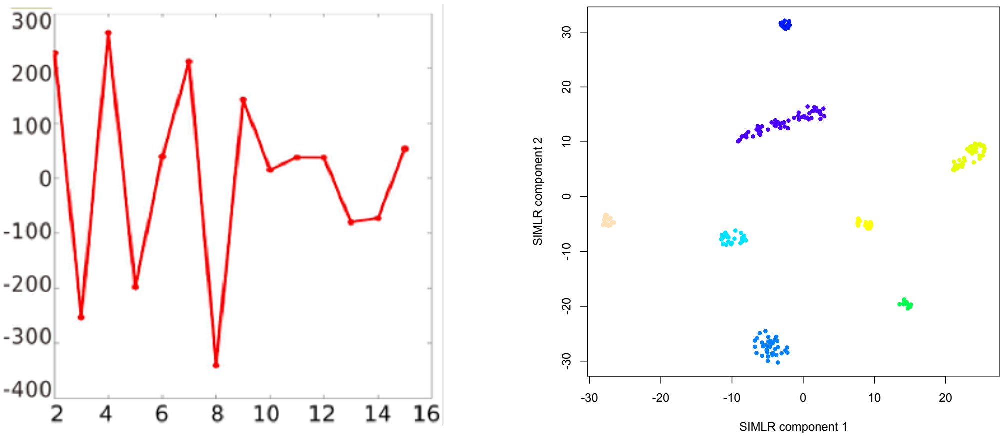

C. Overall survival (C=8) **D.** Features (C=8)

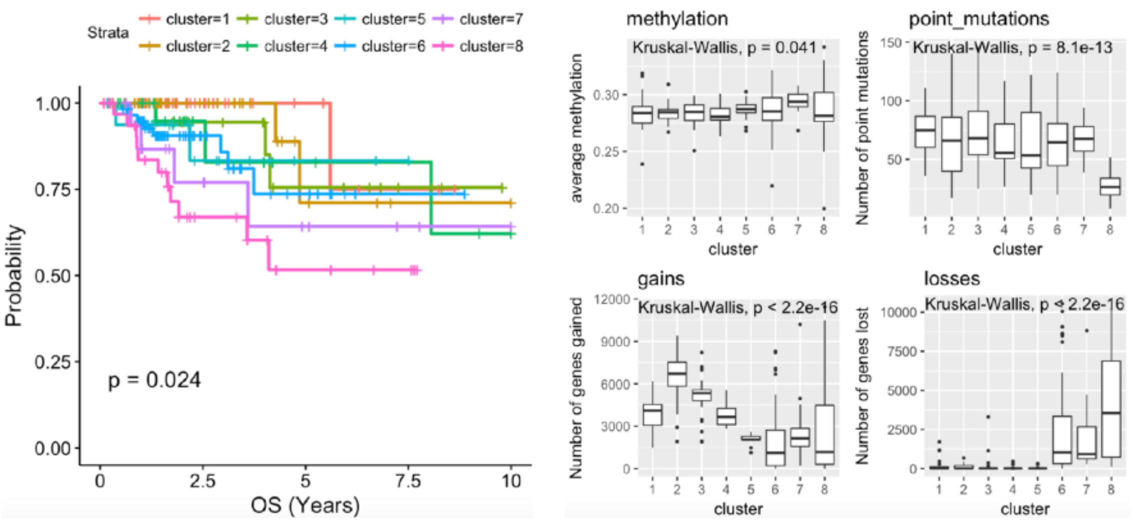

Supplementary Figure 22: TCGA - Papillary Renal Cell Carcinoma. A. Separation cost (y-axis) for different numbers of clusters (x-axis). A lower y-axis value indicates a number of clusters that fits the data better than the previous number. **B.** 2-D visualization of clusters. **C.** Kaplan-Meier curves showing overall survival for the clusters. Survival data was censored as described in Methods. P-value is from log-rank test. **D.** Boxplots showing average methylation beta values, number of genes with somatic coding point mutations, number of genes with copy number gain and number of genes with copy number loss, for each cluster.

Supplementary Figure 23: TCGA - Papillary Thyroid Carcinoma

A. Number of clusters

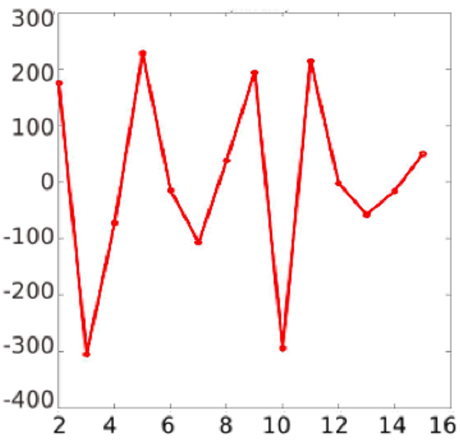

B. Visualization (C=10)

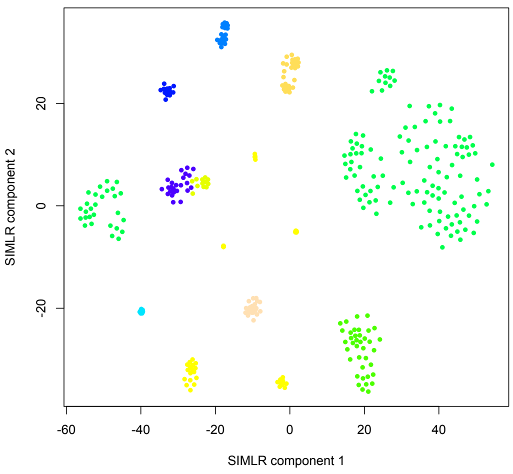

C. Overall survival (C=10)

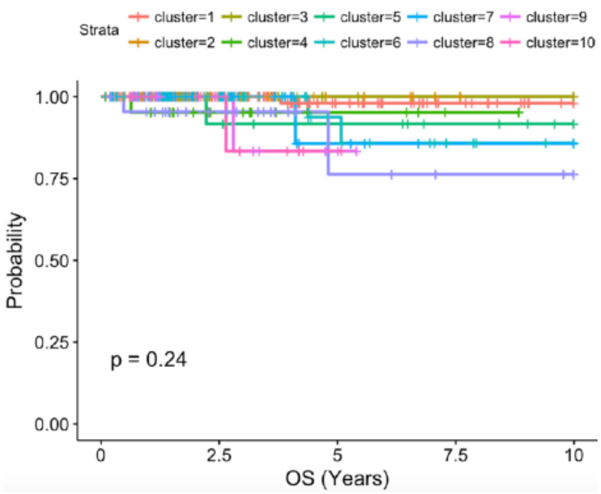

D. Features (C=10)

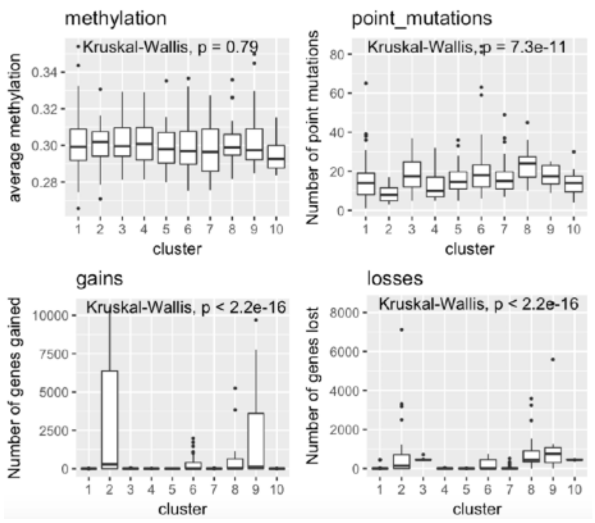

Supplementary Figure 23: TCGA - Papillary Thyroid Carcinoma. A. Separation cost (y-axis) for different numbers of clusters (x-axis). A lower y-axis value indicates a number of clusters that fits the data better than the previous number. **B**. 2-D visualization of clusters. **C**. Kaplan-Meier curves showing overall survival for the clusters. Survival data was censored as described in Methods. P-value is from log-rank test. **D**. Boxplots showing average methylation beta values, number of genes with somatic coding point mutations, number of genes with copy number gain and number of genes with copy number loss, for each cluster.

Supplementary Figure 24: TCGA - Pheochromocytoma and Paraganglioma

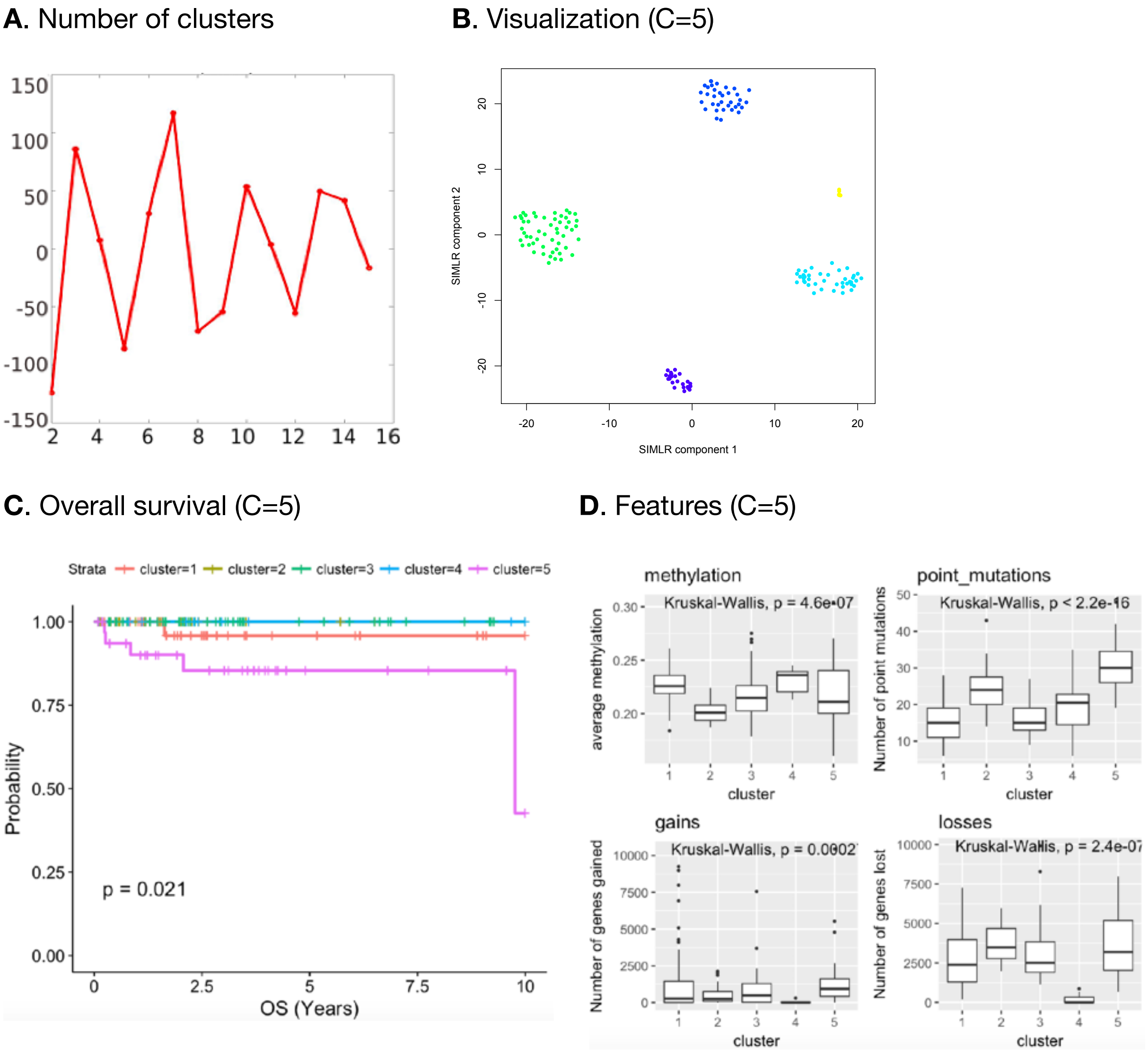

Supplementary Figure 24: TCGA - Pheochromocytoma and Paraganglioma. A. Separation cost (y-axis) for different numbers of clusters (x-axis). A lower y-axis value indicates a number of clusters that fits the data better than the previous number. **B**. 2-D visualization of clusters. **C**. Kaplan-Meier curves showing overall survival for the clusters. Survival data was censored as described in Methods. P-value is from log-rank test. **D**. Boxplots showing average methylation beta values, number of genes with somatic coding point mutations, number of genes with copy number gain and number of genes with copy number loss, for each cluster.

Supplementary Figure 25: TCGA - Pleural Mesothelioma

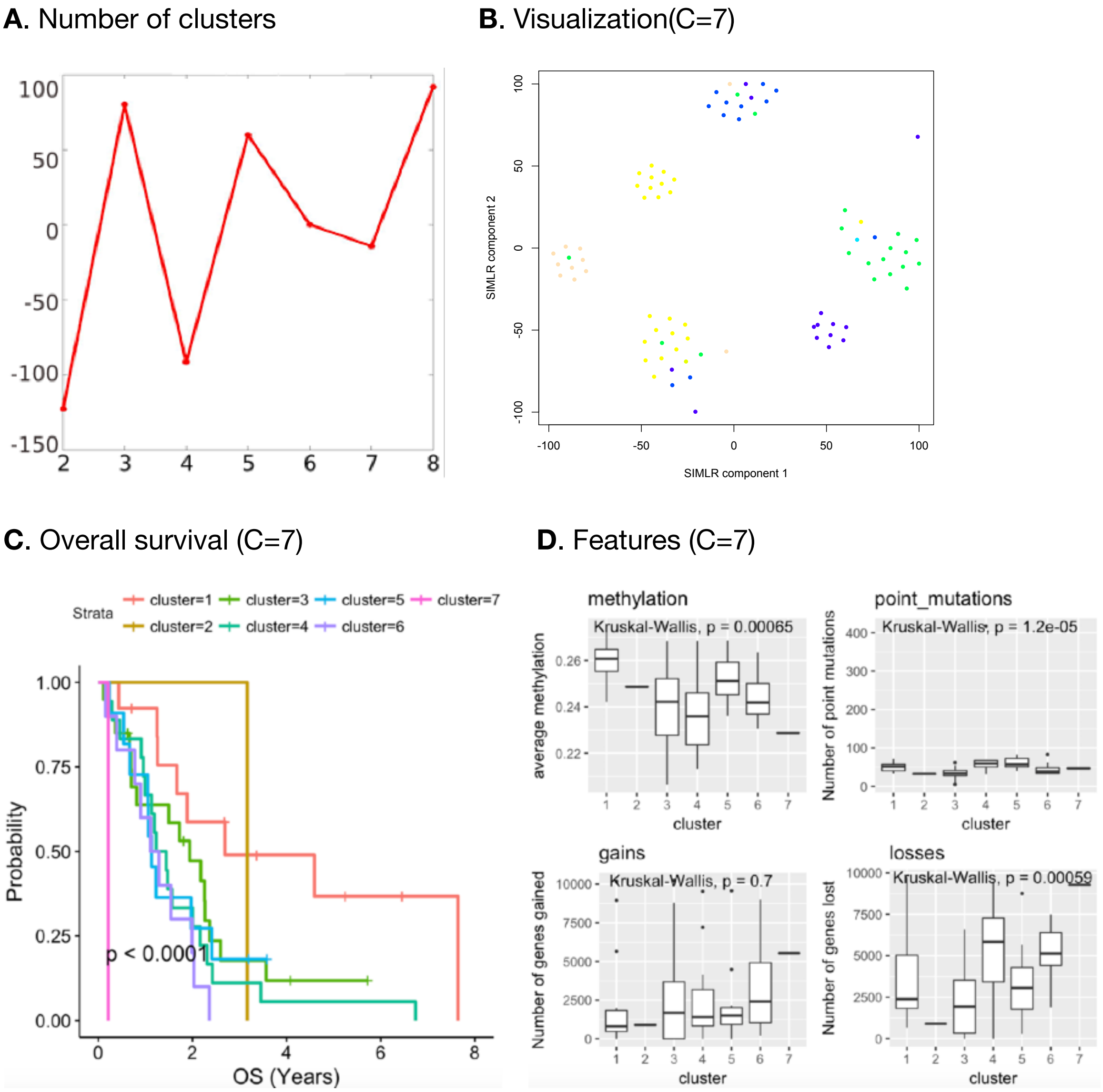

Supplementary Figure 25: TCGA - Pleural Mesothelioma. A. Separation cost (y-axis) for different numbers of clusters (x-axis). A lower y-axis value indicates a number of clusters that fits the data better than the previous number. **B**. 2-D visualization of clusters. **C**. Kaplan-Meier curves showing overall survival for the clusters. Survival data was censored as described in Methods. P-value is from log-rank test. **D**. Boxplots showing average methylation beta values, number of genes with somatic coding point mutations, number of genes with copy number gain and number of genes with copy number loss, for each cluster.

Supplementary Figure 26: TCGA - Prostate Cancer

A. Number of clusters

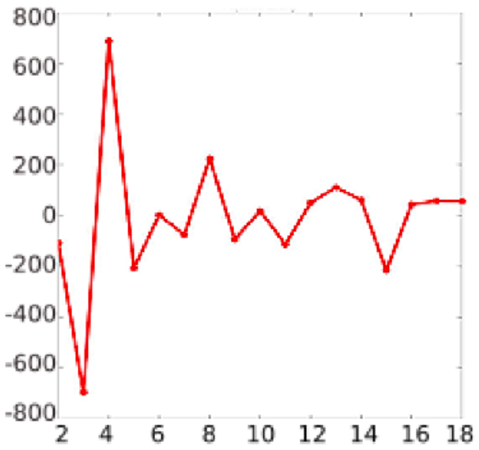

B. Visualization (C=3)

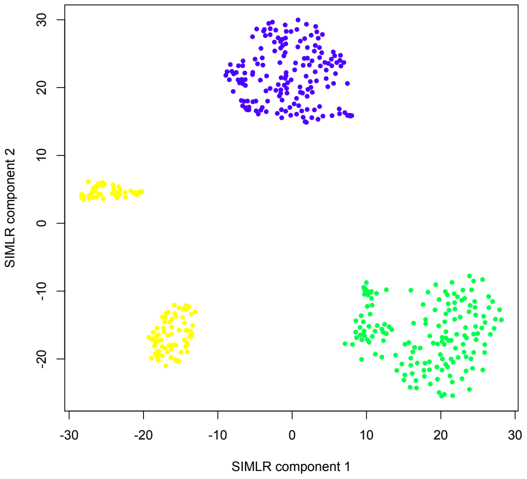

C. Features (C=3)

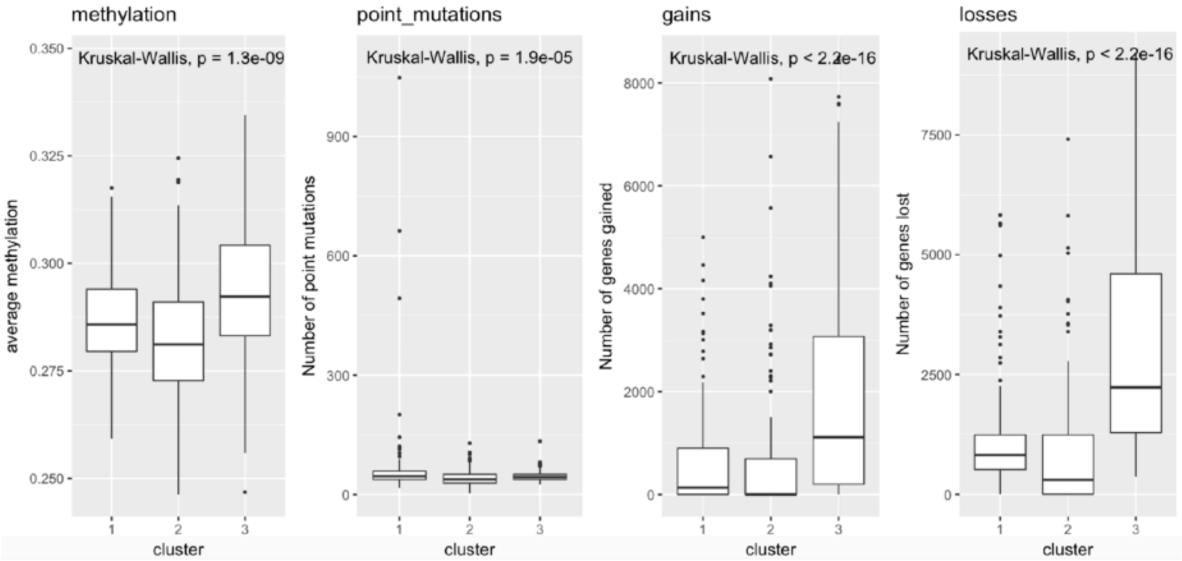

Supplementary Figure 26: TCGA - Prostate Cancer. A. Separation cost (y-axis) for different numbers of clusters (x-axis). A lower y-axis value indicates a number of clusters that fits the data better than the previous number. **B**. 2-D visualization of clusters. **C**. Boxplots showing average methylation beta values, number of genes with somatic coding point mutations, number of genes with copy number gain and number of genes with copy number loss, for each cluster.

Supplementary Figure 27: TCGA - Sarcoma

A. Number of clusters

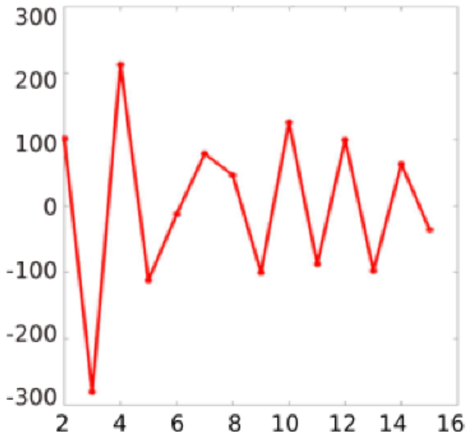

B. Visualization (C=5)

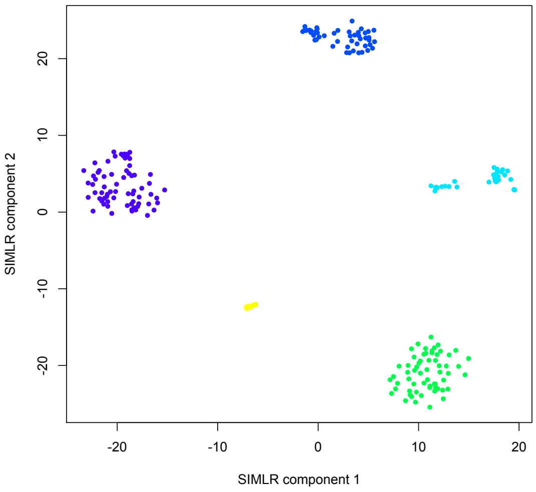

C. Overall survival (C=5)

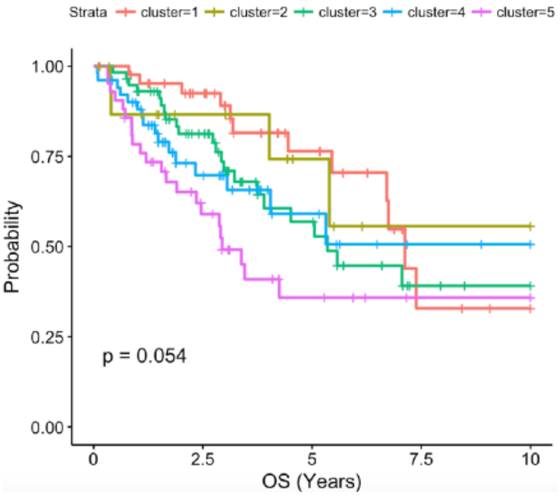

D. Features (C=5)

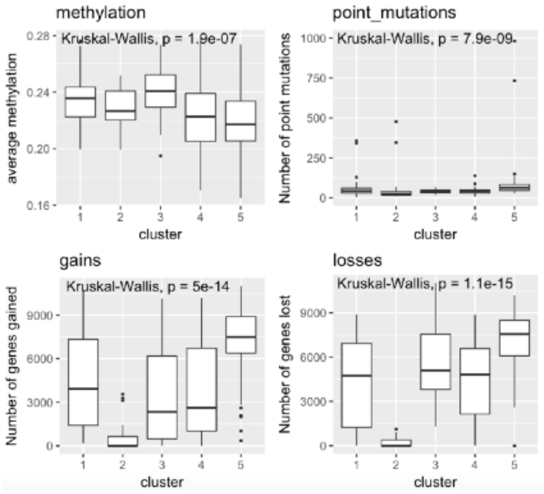

Supplementary Figure 27: TCGA - Sarcoma. A. Separation cost (y-axis) for different numbers of clusters (x-axis). A lower y-axis value indicates a number of clusters that fits the data better than the previous number. **B**. 2-D visualization of clusters. **C**. Kaplan-Meier curves showing overall survival for the clusters. Survival data was censored as described in Methods. P-value is from log-rank test. **D**. Boxplots showing average methylation beta values, number of genes with somatic coding point mutations, number of genes with copy number gain and number of genes with copy number loss, for each cluster.

Supplementary Figure 28: TCGA - Squamous Cell Lung Cancer

A. Number of clusters

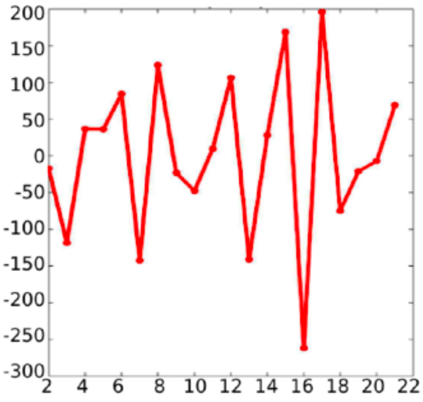

B. Visualization (C=7)

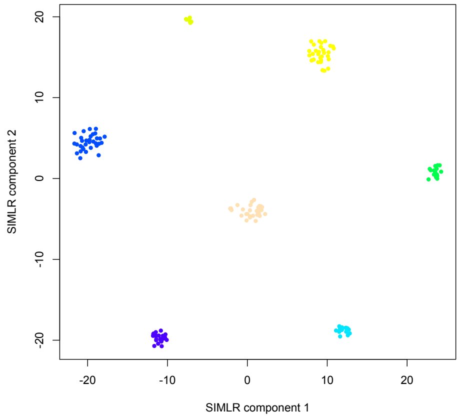

C. Overall survival (C=7)

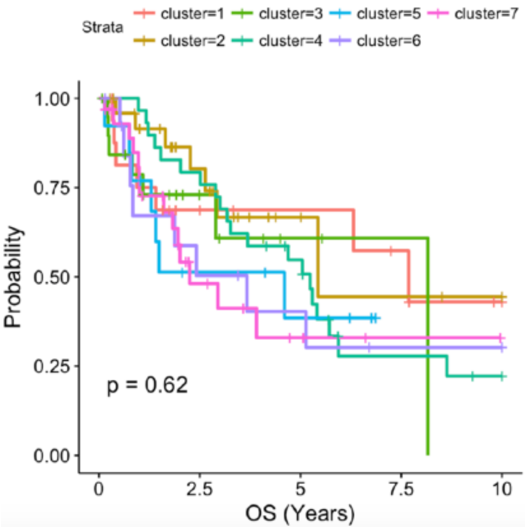

D. Features (C=7)

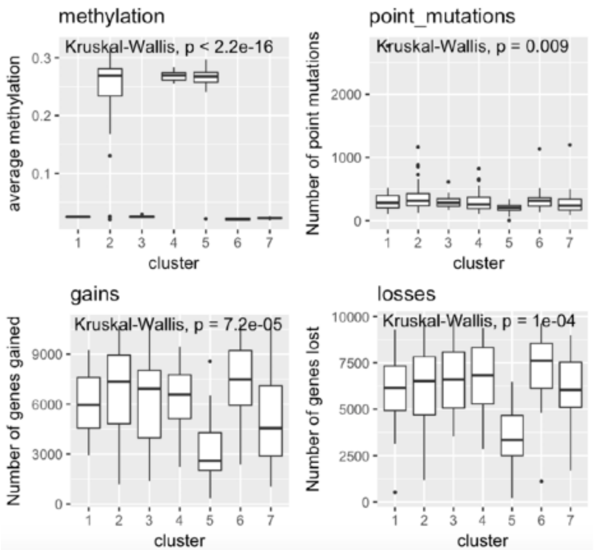

Supplementary Figure 28: TCGA - Squamous Cell Lung Cancer. A. Separation cost (y-axis) for different numbers of clusters (x-axis). A lower y-axis value indicates a number of clusters that fits the data better than the previous number. **B**. 2-D visualization of clusters. **C**. Kaplan-Meier curves showing overall survival for the clusters. Survival data was censored as described in Methods. P-value is from log-rank test. **D**. Boxplots showing average methylation beta values, number of genes with somatic coding point mutations, number of genes with copy number gain and number of genes with copy number loss, for each cluster.

Supplementary Figure 29: TCGA - Testicular Germ Cell Cancer

A. Number of clusters

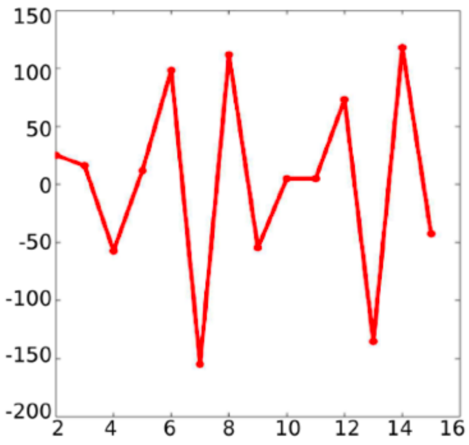

B. Visualization (C=7)

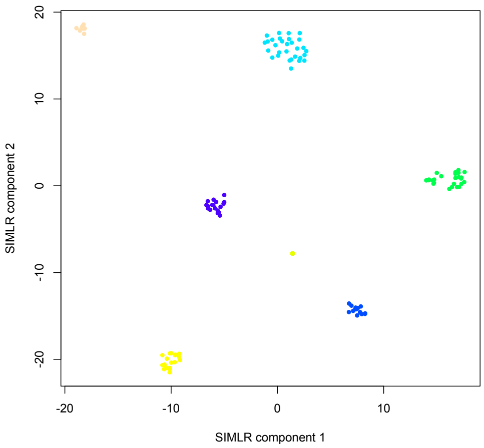

C. Overall survival (C=7)

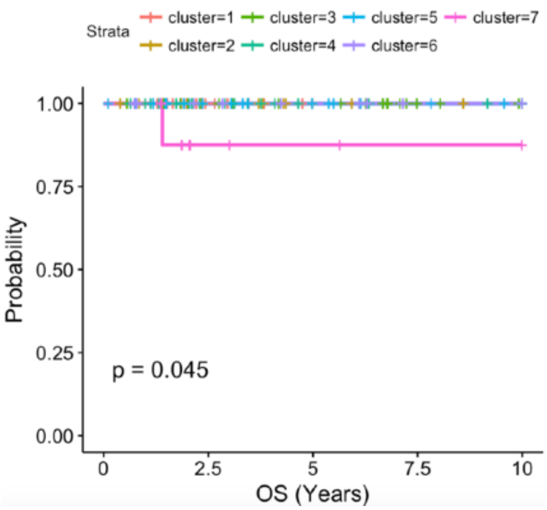

D. Features (C=7)

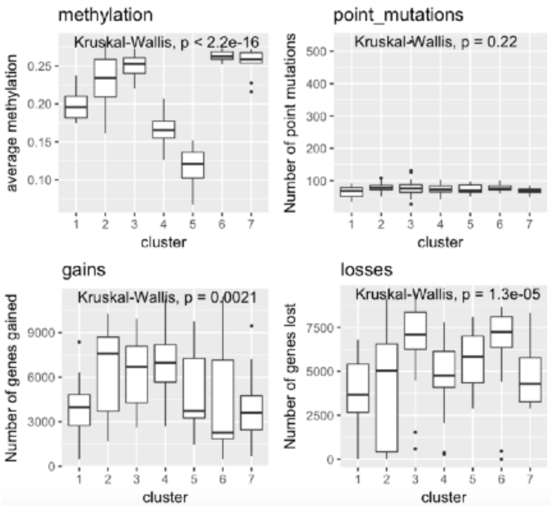

Supplementary Figure 29: TCGA - Testicular Germ Cell Cancer. A. Separation cost (y-axis) for different numbers of clusters (x-axis). A lower y-axis value indicates a number of clusters that fits the data better than the previous number. **B**. 2-D visualization of clusters. **C**. Kaplan-Meier curves showing overall survival for the clusters. Survival data was censored as described in Methods. P-value is from log-rank test. **D**. Boxplots showing average methylation beta values, number of genes with somatic coding point mutations, number of genes with copy number gain and number of genes with copy number loss, for each cluster.

Supplementary Figure 30: TCGA - Urothelial Bladder Carcinoma

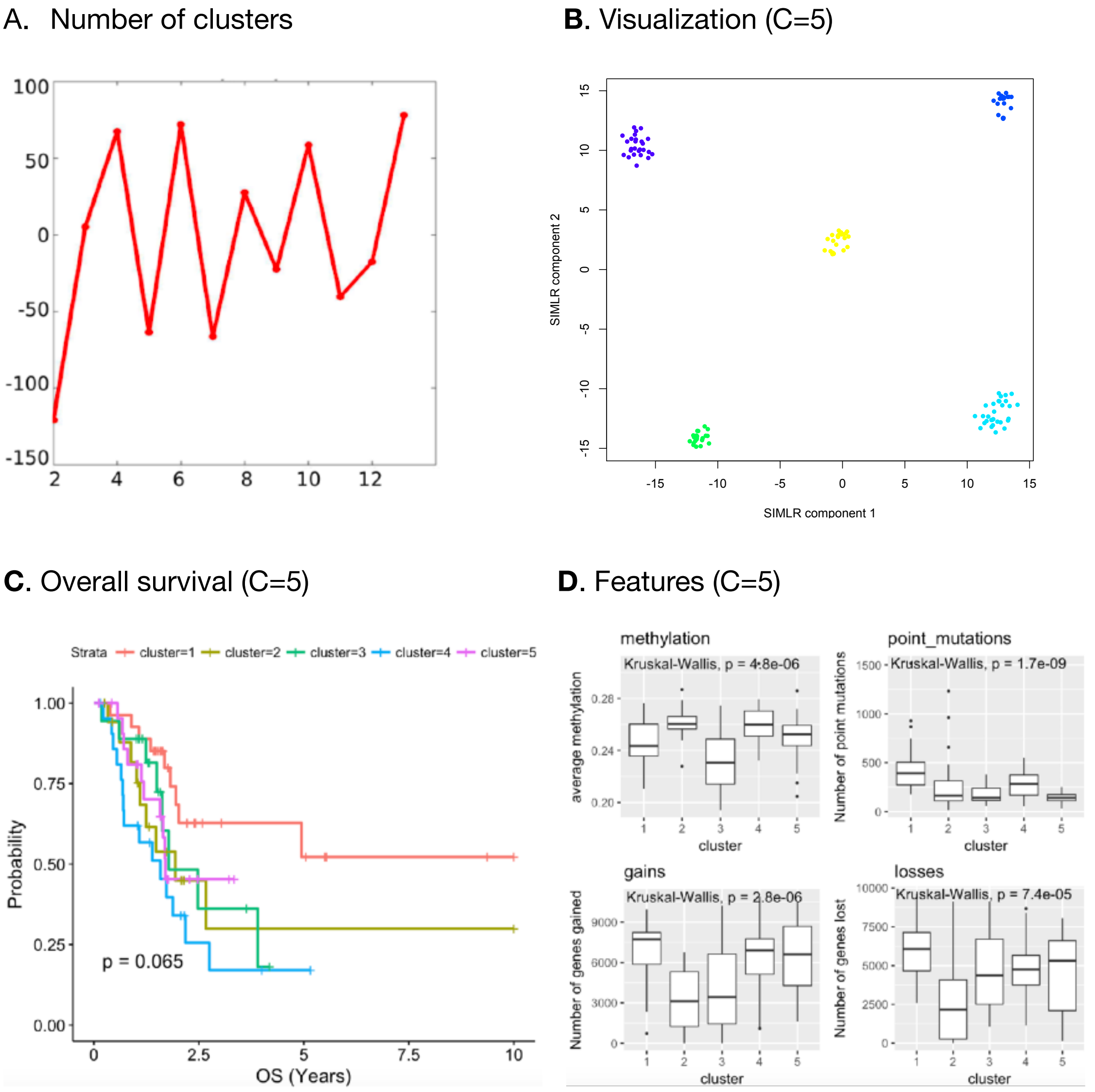

Supplementary Figure 30: TCGA - Urothelial Bladder Carcinoma. A. Separation cost (y-axis) for different numbers of clusters (x-axis). A lower y-axis value indicates a number of clusters that fits the data better than the previous number. **B**. 2-D visualization of clusters. **C.** Kaplan-Meier curves showing overall survival for the clusters. Survival data was censored as described in Methods. P-value is from log-rank test. **D**. Boxplots showing average methylation beta values, number of genes with somatic coding point mutations, number of genes with copy number gain and number of genes with copy number loss, for each cluster.

Supplementary Figure 31: TCGA - Uterine Carcinosarcoma

A. Number of clusters

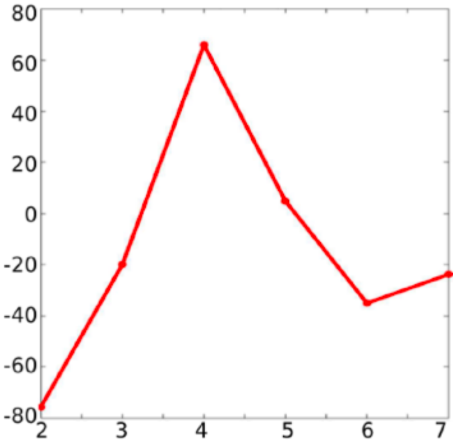

B. Visualization (C=2)

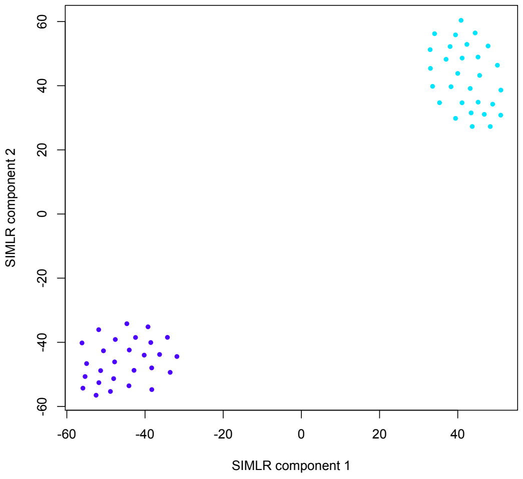

C. Overall survival (C=2)

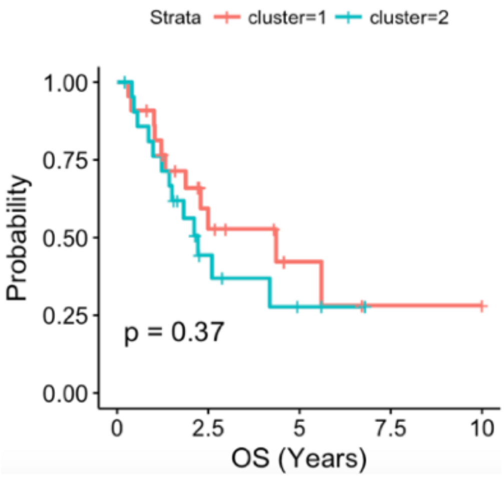

D. Features (C=2)

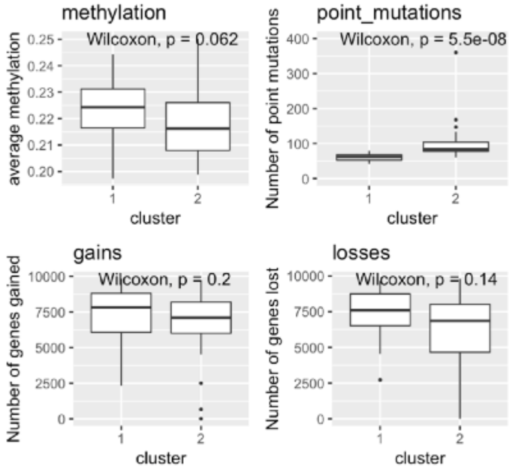

Supplementary Figure 31: TCGA - Uterine Carcinosarcoma. A. Separation cost (y-axis) for different numbers of clusters (x-axis). A lower y-axis value indicates a number of clusters that fits the data better than the previous number. **B**. 2-D visualization of clusters. **C**. Kaplan-Meier curves showing overall survival for the clusters. Survival data was censored as described in Methods. P-value is from log-rank test. **D**. Boxplots showing average methylation beta values, number of genes with somatic coding point mutations, number of genes with copy number gain and number of genes with copy number loss, for each cluster.

Supplementary Figure 32: TCGA - Uveal Melanoma

A. Number of clusters

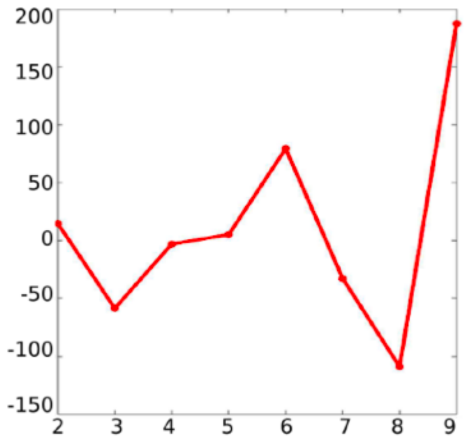

B. Visualization (C=3)

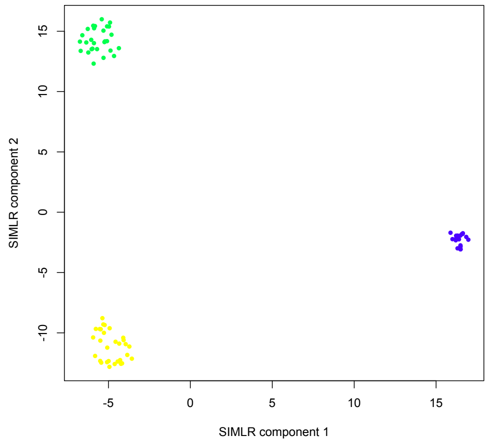

C. Overall survival (C=3)

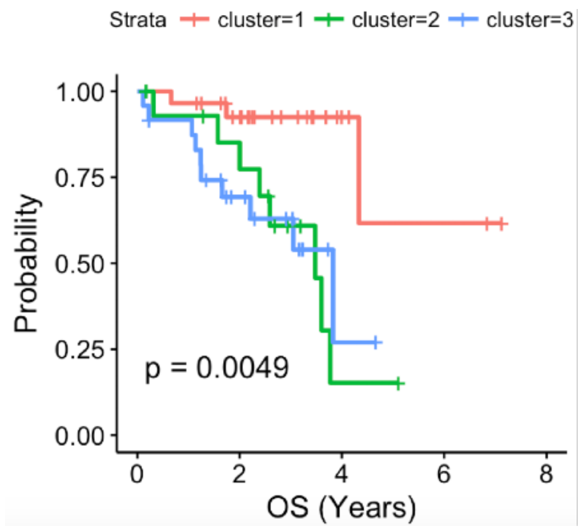

D. Features (C=3)

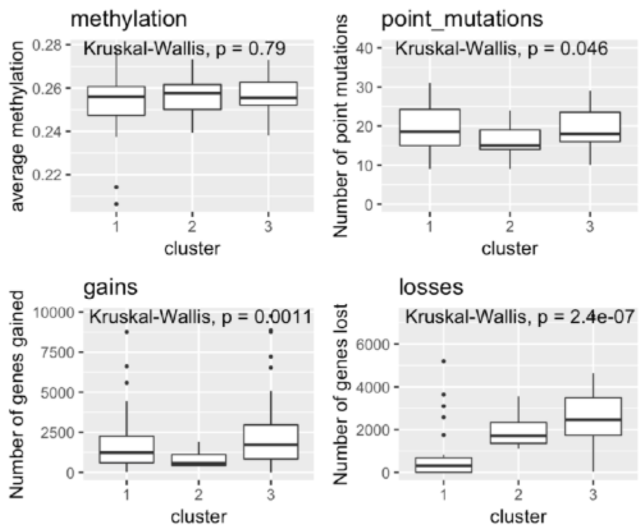

Supplementary Figure 32: TCGA - Uveal Melanoma. A. Separation cost (y-axis) for different numbers of clusters (x-axis). A lower y-axis value indicates a number of clusters that fits the data better than the previous number. **B**. 2-D visualization of clusters. **C**. Kaplan-Meier curves showing overall survival for the clusters. Survival data was censored as described in Methods. P-value is from log-rank test. **D**. Boxplots showing average methylation beta values, number of genes with somatic coding point mutations, number of genes with copy number gain and number of genes with copy number loss, for each cluster.

Supplementary Figure 33: TARGET - Acute Myeloid Leukemia

A. Number of clusters

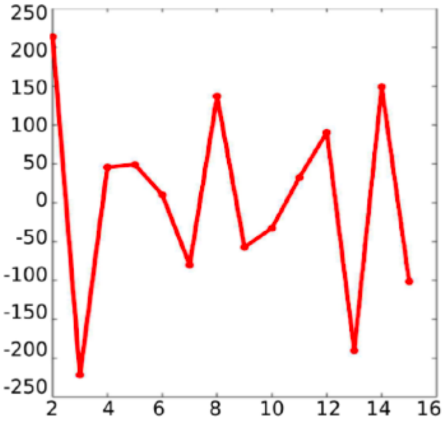

B. Visualization (C=3)

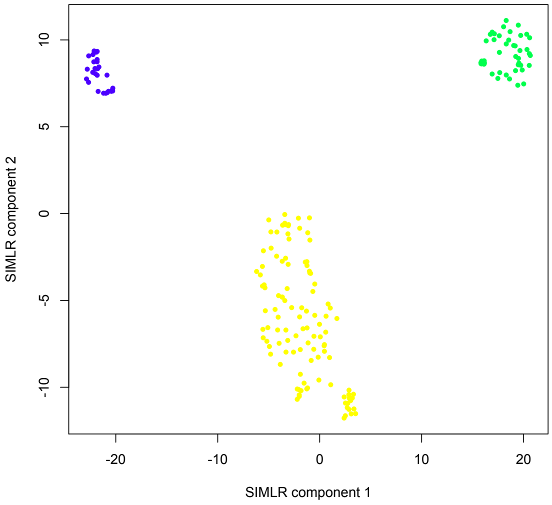

C. Overall Survival (C=3)

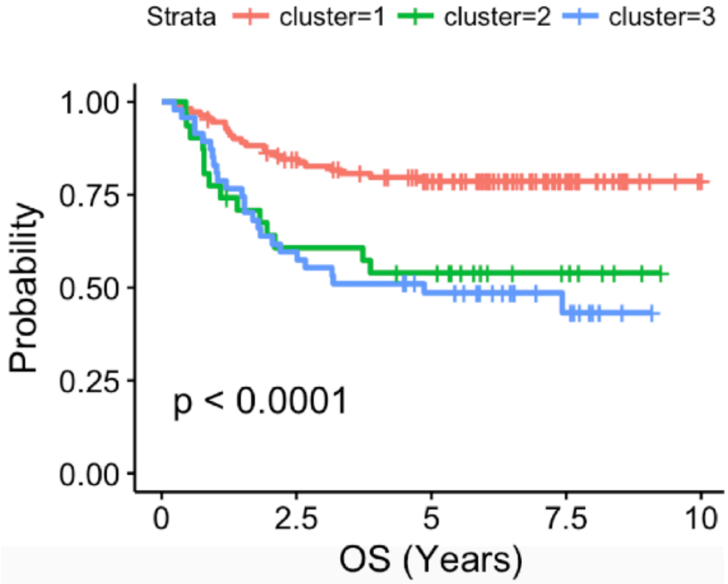

Supplementary Figure 33: TARGET - Acute Myeloid Leukemia. **A.** Separation cost (y-axis) for different numbers of clusters (x-axis). A lower y-axis value indicates a number of clusters that fits the data better than the previous number. **B**. 2-D visualization of clusters. **C**. Kaplan-Meier curves showing overall survival for the clusters. Survival data was censored as described in Methods. P-value is from log-rank test.

Supplementary Figure 34: TARGET - Kidney Wilms Tumor

A. Number of clusters

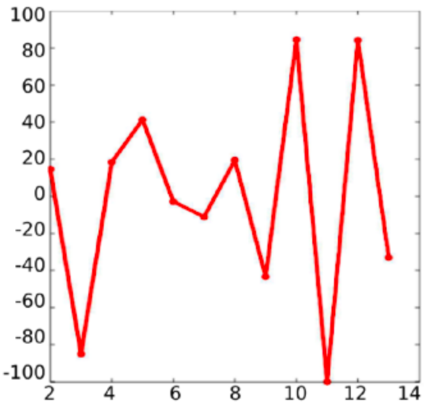

B. Visualization (C=11)

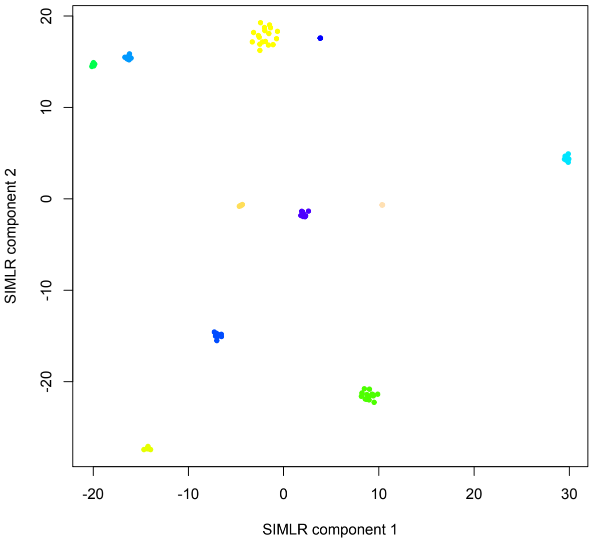

C. Overall Survival (C=11)

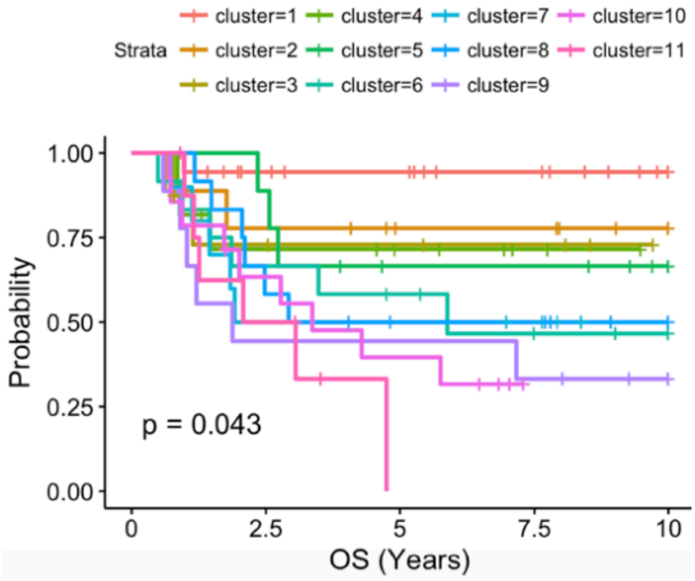

Supplementary Figure 34: TARGET - Kidney Wilms Tumor. A. Separation cost (y-axis) for different numbers of clusters (x-axis). A lower y-axis value indicates a number of clusters that fits the data better than the previous number. **B**. 2-D visualization of clusters. **C**. Kaplan-Meier curves showing overall survival for the clusters. Survival data was censored as described in Methods. P-value is from log-rank test.

Supplementary Figure 35: TARGET - Neuroblastoma

A. Number of clusters

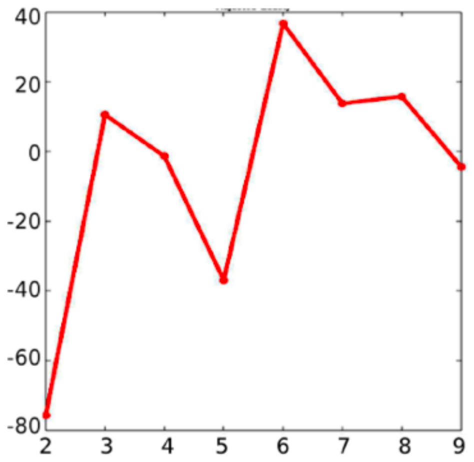

B. Visualization

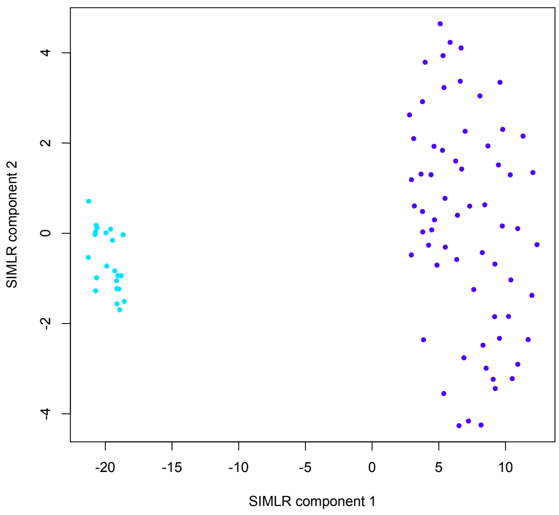

C. Overall Survival

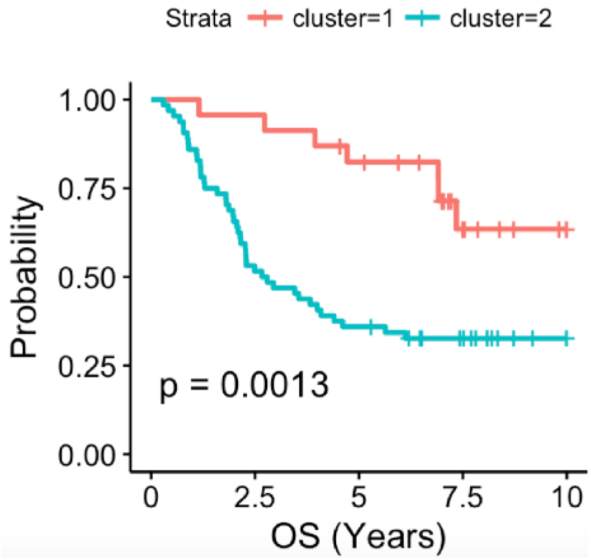

Supplementary Figure 35: TARGET - Neuroblastoma. A. Separation cost (y-axis) for different numbers of clusters (x-axis). A lower y-axis value indicates a number of clusters that fits the data better than the previous number. **B**. 2-D visualization of clusters. **C**. Kaplan-Meier curves showing overall survival for the clusters. Survival data was censored as described in Methods. P-value is from log-rank test.

Supplementary Figure 36: TARGET - Osteosarcoma

A. Number of clusters

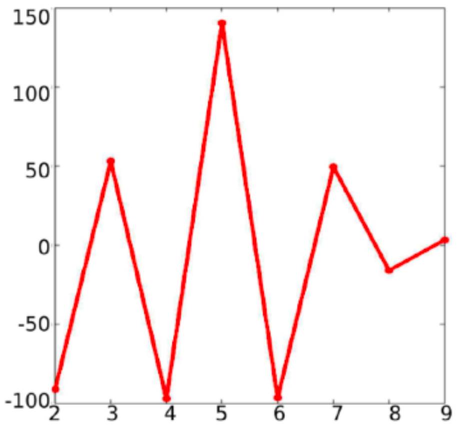

B. Visualization (C=6)

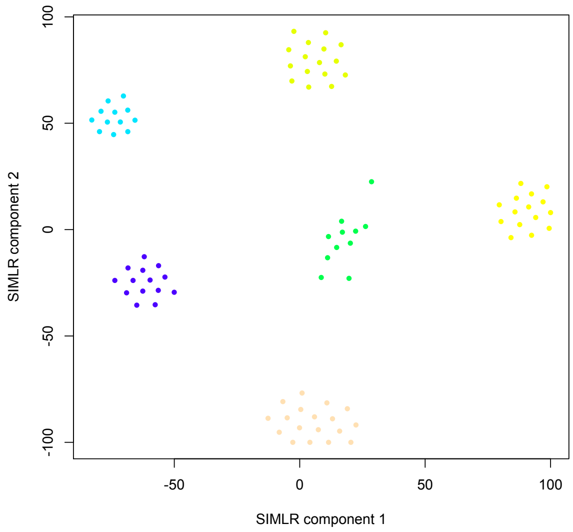

C. Overall Survival (C=6)

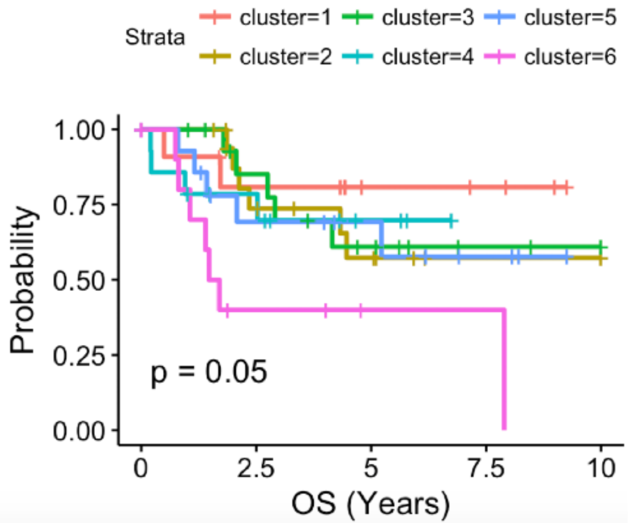

Supplementary Figure 36: TARGET - Osteosarcoma. A. Separation cost (y-axis) for different numbers of clusters (x-axis). A lower y-axis value indicates a number of clusters that fits the data better than the previous number. **B**. 2-D visualization of clusters. **C**. Kaplan-Meier curves showing overall survival for the clusters. Survival data was censored as described in Methods. P-value is from log-rank test.

Supplementary Figure 37: Kernel weight distribution for lower-grade glioma

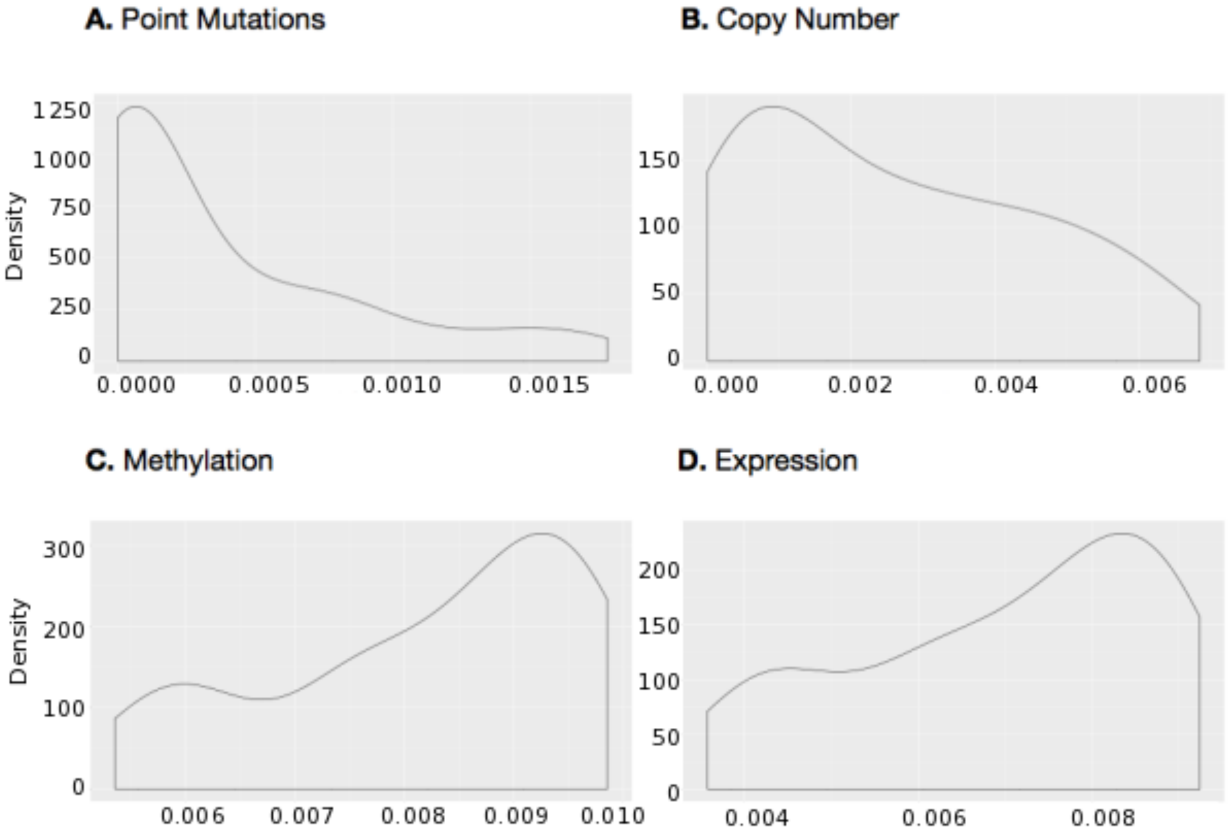

Supplementary Figure 37: Kernel weight distribution for lower-grade glioma. Distribution of the weights of the 55 kernels for lower-grade gliomas, for kernels based on **A**. Point mutations **B**. Copy number **C**. Methylation **D**. Expression

Supplementary Table 1: Survival analysis and clustering quality for subtypes discovered by CIMLR in 32 cancer types from TCGA.

CANCER	SAMPLES	CLUSTERS	OS P-VALUE	DSS P-VALUE	DFI P-VALUE	PFI P-VALUE	Silhouette	Stability
acute_myeloid_leukemia	160	13	1.12E-03	NA	NA	NA	0.87	0.92
adrenocortical_carcinoma	74	6	6.77E-06	8.73E-06	3.90E-04	3.95E-10	0.79	0.89
breast_cancer	663	13	2.53E-03	2.12E-03	5.45E-03	3.57E-02	0.76	0.92
cervical_cancer	190	11	4.86E-01	8.59E-01	3.8E-01	7.57E-01	0.92	0.92
cholangiocarcinoma	34	3	2.43E-01	2.16E-01	5.63E-01	9.19E-02	0.94	0.85
chromophobe_renal_cell_carcinoma	65	7	5.03E-06	3.39E-09	7.01E-01	2.47E-04	0.67	0.88
clear_cell_renal_cell_carcinoma	260	10	1.92E-06	1.03E-07	4.91E-01	4.98E-06	0.87	0.92
colon_rectal_cancer	189	11	4.66E-01	2.43E-01	1.3E-01	3.39E-01	0.92	0.92
cutaneous_melanoma	262	4	4.74E-08	7.05E-08	NA	8.63E-03	0.89	0.90
endometrial_carcinoma	106	11	8.76E-01	6.01E-01	2.07E-01	4.75E-01	0.85	0.91
gastric_adenocarcinoma	328	3	9.93E-02	2.2E-01	3.99E-01	3.27E-02	0.74	0.86
glioblastoma	118	8	1.07E-02	5.59E-03	NA	1.46E-01	0.93	0.91
head_neck_squamous_cell_carcinoma	495	8	8.56E-03	6.20E-03	2.69E-01	8.42E-03	0.86	0.90
liver_hepatocellular_carcinoma	359	8	2.70E-04	4.70E-03	7.68E-02	1.89E-02	0.87	0.90
lower_grade_glioma	282	3	1.79E-24	1.22E-24	2.09E-01	6.12E-23	0.77	0.89
lung_adenocarcinoma	188	8	2.14E-03	6.90E-03	4.68E-01	3.95E-02	0.91	0.91
lymphoid_neoplasm_diffuse_large_b_cell_lymphoma	47	3	6.02E-01	3.93E-01	3.98E-01	9.8E-01	0.83	0.90
oesophageal_carcinoma	182	3	3.78E-01	1.55E-01	9.57E-02	1.45E-01	0.87	0.90
ovarian_carcinoma	183	2	1.44E-02	5.34E-03	2.86E-01	2.44E-01	0.83	1.00
pancreatic_adenocarcinoma	148	12	3.03E-02	3.64E-04	9.98E-02	3.72E-02	0.90	0.92
papillary_renal_cell_carcinoma	264	8	2.36E-02	8.49E-06	2.54E-02	1.21E-05	0.85	0.90
papillary_thyroid_carcinoma	388	10	2.37E-01	4.69E-02	3.31E-01	2.71E-01	0.26	0.72
pheochromocytoma_paraganglioma	162	5	2.07E-02	2.62E-02	4.72E-02	1.41E-02	0.84	0.91
pleural_mesothelioma	81	7	2.89E-02	2.62E-02	5.18E-02	7.07E-06	-0.20	0.53
prostate_cancer	490	3	5.56E-01	1.79E-01	2.04E-03	7.38E-05	0.72	1.00
sarcoma	240	5	5.44E-02	7.48E-02	6.52E-01	6.9E-01	0.84	0.91
squamous_cell_lung_cancer	176	7	6.23E-01	9.14E-01	1.10E-02	8.00E-01	0.93	0.90
testicular_germ_cell_cancer	132	7	4.51E-02	3.1E-01	1.57E-01	1.9E-01	0.92	0.90
thymoma	118	7	3.57E-02	1.43E-01	NA	4.43E-01	0.90	0.91
urothelial_bladder_carcinoma	126	5	6.55E-02	1.22E-01	1.97E-01	5.64E-01	0.93	0.87
uterine_carcinosarcoma	56	2	3.75E-01	2.82E-01	4.36E-01	3.19E-01	0.89	1.00
uveal_melanoma	79	3	4.94E-03	2.65E-03	NA	6.81E-04	0.94	0.89
TOTAL SIGNIFICANT (P<0.05)			19	17	6	16		
TOTAL SURVIVAL SIGNIFICANT (any metric)	23							

Supplementary Table 1: Survival analysis and clustering quality for subtypes discovered by CIMLR in 32 cancer types from TCGA. Survival analysis was done using four outcome metrics: Overall Survival (OS), Disease-Specific Survival (DSS), Progression Free Interval (PFI) and Disease Free Interval (DFI), over a time interval of 10 years. For Overall Survival (OS), we censored data points corresponding to patients who died within 30 days or were over the age of 80 at the beginning of the observation period. Associations between subtypes and outcome were then calculated by Kaplan-Meier analysis using a log-rank test. CIMLR subtypes were found to be significantly associated with Overall Survival in 19 cancer types, with DSS in 17 cancer types, with DFI in 6 cancer types, and with PFI in 16 cancer types. CIMLR subtypes were significantly associated with at least one outcome metric in 23 of 32 cancer types. Stability is measured as the normalized mutual information of the results over 100 new independent runs of k-means with respect to the original results.

Supplementary Table 2: Association of CIMLR clusters with pathway activity

CANCER	EGFR	Hypoxia	JAK-STAT	MAPK	NFkB	PI3K	TGFb	TNFa	Trail	VEGF	p53
acute_myeloid_leukemia	1.64E-01	1.94E-02	1.59E-01	3.36E-01	1.52E-01	2.01E-01	1.94E-02	1.59E-01	1.59E-01	1.04E-01	1.09E-01
adrenocortical_carcinoma	1.47E-01	8.41E-01	2.87E-01	4.71E-03	4.71E-03	4.75E-02	1.1E-01	1.1E-01	2.58E-03	1.47E-01	4.71E-03
breast_tumours	4.39E-19	9.18E-22	2.37E-13	7.56E-22	8.14E-42	1.77E-43	1.13E-08	8.14E-42	5.30E-20	6.97E-12	7.89E-31
cervical_cancer	5.80E-06	7.15E-04	4.37E-05	1.12E-03	4.76E-05	2.03E-09	2.17E-01	1.39E-05	4.22E-07	4.95E-03	1.34E-11
cholangiocarcinoma	7.32E-01	3.33E-01	4.58E-01	7.32E-01	2.9E-01	3.43E-01	7.32E-01	2.9E-01	3.33E-01	7.32E-01	7.32E-01
chromophobe_renal_cell_carcinoma	5.07E-01	3.83E-01	3.83E-01	6.48E-01	5.07E-01	6.48E-01	6.48E-01	6.48E-01	3.83E-01	3.83E-01	4.11E-03
clear_cell_renal_cell_carcinoma	4.01E-04	4.93E-03	5.57E-03	2.08E-02	8.70E-07	5.85E-02	1.94E-03	1.01E-08	4.01E-04	2.11E-02	1.05E-01
gastric_adenocarcinoma	1.42E-01	3.54E-01	1.47E-04	2.11E-02	3.42E-04	1.29E-03	1.39E-06	1.40E-02	4.22E-09	4.50E-06	2.85E-03
glioblastoma	1.13E-03	1.17E-02	2.46E-01	1.35E-04	1.35E-04	5.79E-03	1.03E-02	1.35E-04	1.60E-02	1.73E-04	8.50E-04
head_neck_squamous_cell_carcinoma	1.38E-32	6.81E-06	5.51E-24	9.03E-26	2.31E-14	3.92E-16	1.25E-13	6.18E-15	1.82E-17	2.86E-34	3.63E-12
liver_hepatocellular_carcinoma	5.13E-21	2.04E-16	5.45E-12	5.28E-21	2.90E-19	1.23E-19	3.42E-17	6.46E-19	3.11E-14	2.05E-06	6.92E-15
lower_grade_glioma	7.80E-10	3.93E-01	4.55E-13	2.86E-22	3.90E-16	4.56E-01	1.73E-05	3.05E-15	3.19E-04	9.12E-03	6.61E-24
lung_adenocarcinoma	7.49E-03	4.80E-05	4.43E-08	3.43E-03	1.40E-08	6.36E-04	1.73E-04	3.84E-08	3.84E-08	7.93E-07	3.64E-07
lymphoid_neoplasm_diffuse_large_b_cell_lymphoma	2.26E-01	4.25E-01	1.88E-01	4.86E-01	4.25E-01	1.09E-01	5.88E-01	1.5E-01	3.20E-02	4.95E-01	3.21E-02
oesophageal_carcinoma	6.87E-01	8.32E-15	2.21E-01	8.1E-01	5.50E-02	5.00E-01	1.14E-02	1.51E-01	3.04E-02	7.23E-02	1.10E-11
ovarian_carcinoma	5.32E-03	3.39E-01	9.83E-01	8.48E-02	3.63E-01	3.72E-01	1.78E-01	2.46E-01	1.88E-02	3.72E-01	9.83E-01
pancreatic_adenocarcinoma	1.38E-05	1.85E-04	4.22E-02	1.45E-06	1.49E-03	1.85E-04	1.85E-04	1.62E-02	3.57E-10	1.85E-04	3.77E-04
papillary_renal_cell_carcinoma	9.29E-04	5.45E-04	9.62E-02	1.55E-06	1.55E-01	1.73E-03	4.14E-02	1.17E-01	3.39E-04	3.39E-04	6.09E-04
pheochromocytoma_paraganglioma	8.04E-01	3.20E-06	2.20E-02	8.04E-01	4.88E-01	2.10E-02	2.10E-02	2.32E-01	3.62E-01	5.21E-01	1.05E-01
prostate_cancer	3.64E-04	1.87E-04	1.96E-02	1.87E-04	9.19E-05	8.82E-02	9.19E-05	7.55E-05	3.61E-08	1.45E-05	6.94E-19
sarcoma	9.24E-16	4.96E-08	5.43E-08	1.09E-17	1.70E-21	5.63E-06	6.97E-01	1.21E-21	2.17E-09	4.26E-10	4.57E-11
squamous_cell_lung_cancer	3.83E-04	7.14E-01	8.38E-03	3.85E-03	3.18E-07	2.12E-01	8.97E-05	1.77E-07	4.73E-07	1.21E-01	8.39E-07
testicular_germ_cell_cancer	1.51E-16	1.15E-06	2.43E-07	1.07E-13	1.29E-10	1.81E-09	1.51E-16	3.67E-12	2.56E-12	3.06E-02	7.87E-15
thymoma	5.02E-04	1.94E-05	2.10E-04	4.89E-04	2.10E-04	8.24E-04	1.45E-07	1.09E-04	9.98E-09	1.15E-06	9.98E-09
urothelial_bladder_carcinoma	6.76E-05	1.14E-03	7.85E-05	4.50E-05	3.80E-08	2.62E-03	7.85E-05	1.64E-08	1.34E-07	9.43E-02	6.76E-05
uterine_carcinosarcoma	9.54E-01	9.54E-01	1.18E-02	9.54E-01	9.54E-01	9.54E-01	9.54E-01	9.54E-01	9.4E-01	9.54E-01	9.54E-01
uveal_melanoma	2.64E-02	4.81E-01	1.59E-04	9.41E-03	6.06E-05	9.54E-04	8.09E-02	6.06E-05	2.42E-01	4.87E-01	9.54E-04
TOTAL SIGNIFICANT	18	17	18	19	18	16	18	17	21	15	21
TOTAL SIGNIFICANT (ANY PATHWAY)	26										

Supplementary Table 2: Association of CIMLR clusters with pathway activity. P-values (Kruskal-Wallis test) for difference in pathway activity between the clusters found by CIMLR, for 11 cancer-associated pathways, based on pathway activity values calculated by PROGENy[3]. PROGENy data was available for 27 TCGA cancer types.

Supplementary Table 3: Survival analysis for subtypes discovered by CIMLR in 4 cancer types from TARGET.

CANCER	SAMPLES	CLUSTERS	OS P-VALUE
acute_myeloid_leukemia	189	3	8.47E-05
kidney_wilms_tumor	121	11	4.30E-02
neuroblastoma	87	2	1.30E-03
osteosarcoma	86	6	5E-02
TOTAL SIGNIFICANT			4

Supplementary Table 3: Survival analysis for subtypes discovered by CIMLR in 4 cancer types from TARGET. Results of survival analysis for clusters discovered by CIMLR on 4 cancer types from TARGET. Only Overall Survival (OS) was used as an outcome. Analysis was limited to a time interval of 10 years. We censored data points corresponding to patients who died within 30 days. Associations between subtypes and outcome were then calculated by Kaplan-Meier analysis using a log-rank test. CIMLR subtypes were significantly associated with survival for all 4 cancer types.

Supplementary Table 4: Selecting the number of kernels for CIMLR

	Value for Sigma	Value for k	Number of kernels	NMI
VARYING SIGMA				
	2	11	22	0.7134321
	3	11	33	0.7134321
	5	11	55	1
	6	11	66	1
	11	21	231	1
VARYING K				
	5	3	15	0.96167861
	5	5	25	0.98084178
	5	8	45	0.94737495
	5	11	55	1
	11	21	231	1

Supplementary Table 4: Selecting number of kernels for CIMLR. We applied CIMLR to the multi-omic dataset of 282 lower-grade gliomas from TCGA and assessed the variability of the resulting clusters in terms of normalized mutual information for a variable number of kernels per data type; 55 kernels represent the point where a plateau is reached. The total number of kernels was varied both by varying sigma and by varying K; see the original description of SIMLR[2] for details of these parameters.

Supplementary References

1. Feng, Y. *et al.* GTF2I mutation frequently occurs in more indolent thymic epithelial tumors and predicts better prognosis. *Lung Cancer* **110,** 48–52 (2017).
2. Wang, B., Zhu, J., Pierson, E., Ramazzotti, D. & Batzoglou, S. Visualization and analysis of single-cell RNA-seq data by kernel-based similarity learning. *Nature Methods* **14,** 414–416 (2017).
3. Schubert, M. *et al.* Perturbation-response genes reveal signaling footprints in cancer gene expression. *Nature Communications* **9,** (2018).
