# Supplementary material for: Multi-omic tumor data reveal diversity of molecular mechanisms that correlate with survival
Source: Nat Commun. 2018 Oct 26;9:4453. doi: 10.1038/s41467-018-06921-8 (PMC6203719; doi:10.1038/s41467-018-06921-8)
Supplement: Supplementary file 2 — Description of Additional Supplementary Files [file 41467_2018_6921_MOESM2_ESM.docx]

**Description of Additional Supplementary Files**

File Name: Supplementary Movie

Description:

File Name: Supplementary Data 1

Description: Survival analysis and clustering quality for subtypes discovered by CIMLR using multi-omic and single-omic data in 32 cancer types from TCGA. Survival analysis was done using four outcome metrics: Overall Survival (OS), Disease-Specific Survival (DSS), Progression Free Interval (PFI) and Disease Free Interval (DFI), over a time interval of 10 years. For Overall Survival (OS), we censored data points corresponding to patients who died within 30 days or were over the age of 80 at the beginning of the observation period. Associations between subtypes and outcome were then calculated by KaplanMeier analysis using a log-rank test. Stability is measured as the normalized mutual information of the results over 100 new independent runs of k-means with respect to the original results.

File Name: Supplementary Data 2

Description: Survival analysis and clustering quality for subtypes discovered by CIMLR and other methods in 32 cancer types from TCGA. Survival analysis was done using four outcome metrics: Overall Survival (OS), Disease-Specific Survival (DSS), Progression Free Interval (PFI) and Disease Free Interval (DFI), over a time interval of 10 years. For Overall Survival (OS), we censored data points corresponding to patients who died within 30 days or were over the age of 80 at the beginning of the observation period. Associations between subtypes and outcome were then calculated by Kaplan-Meier analysis using a log-rank test. Stability is measured as the normalized mutual information of the results over 100 new independent runs of k-means with respect to the original results (for methods that use kmeans clustering).

File Name: Supplementary Data 3

Description: Survival analysis and clustering quality for subtypes discovered by CIMLR using multi-omic and single-omic data in 4 cancer types from TARGET. Survival analysis was done using Overall Survival (OS), over a time interval of 10 years. We censored data points corresponding to patients who died within 30 days. Associations between subtypes and outcome were then calculated by Kaplan-Meier analysis using a log-rank test. Stability is measured as the normalized mutual information of the results over 100 new independent runs of k-means with respect to the original results.

File Name: Supplementary Data 4

Description: Survival analysis and clustering quality for subtypes discovered by CIMLR and other methods in 4 cancer types from TARGET. Survival analysis was done using Overall Survival (OS), over a time interval of 10 years. We censored data points corresponding to patients who died within 30 days. Associations between subtypes and outcome were then calculated by Kaplan-Meier analysis using a log-rank test. Stability is measured as the normalized mutual information of the results over 100 new independent runs of k-means with respect to the original results (for methods that use k-means clustering).

File Name: Supplementary Data 5

Description: Results of Cox proportional hazards analysis for 27 cancers from TCGA. CIMLR clusters as well as clinical features were evaluated using univariate Cox regression. The Hazard Ratio for each variable in the regression model is given along with the corresponding 95% confidence intervals and p-values based on the two-sided Wald test. The Concordance Index (CI) and its standard error (SE (CI)) are also given for each regression model. Based on the univariate models, we selected significant (Wald test p<0.1) clinical features, which were then included along with CIMLR clusters in a multivariate Cox regression model. Patient age, gender, race, ethnicity, tumor stage and grade were taken into account where data was available. For prostate cancer, Gleason score was taken into account.

File Name: Supplementary Data 6

Description: Results of Cox proportional hazards analysis in 5 validation datasets. Samples in the validation datasets were assigned to specific high-risk or low-risk groups based on CIMLR clusters, using random forest models. These groups, as well as clinical features, were evaluated using univariate Cox regression. The Hazard Ratio for each variable in the regression model is given along with the corresponding 95% confidence intervals and p-values based on the two-sided Wald test. The Concordance Index (CI) and its standard error (SE (CI)) are also given for each regression model. Based on the univariate models, we selected significant (Wald test p<0.1) clinical features, which were then included along with CIMLR clusters in a multivariate Cox regression model. Patient age, gender, race, ethnicity, tumor stage and grade were taken into account where data was available. For prostate cancer, Gleason score was taken into account.

File Name: Supplementary Data 7

Description: CIMLR cluster assignments for all samples belonging to 32 cancer types from TCGA.

File Name: Supplementary Data 8

Description: CIMLR cluster assignments for all samples belonging to 4 cancer types from TARGET.
